# Novel Thiosemicarbazone Derivatives: In Vitro and In Silico Evaluation as Potential MAO-B Inhibitors

**DOI:** 10.3390/molecules26216640

**Published:** 2021-11-02

**Authors:** Derya Osmaniye, Berkant Kurban, Begüm Nurpelin Sağlık, Serkan Levent, Yusuf Özkay, Zafer Asım Kaplancıklı

**Affiliations:** 1 Department of Pharmaceutical Chemistry, Faculty of Pharmacy, Anadolu University, 26470 Eskişehir, Turkey; berkantkurban@hotmail.com (B.K.); bnsaglik@anadolu.edu.tr (B.N.S.); serkanlevent@anadolu.edu.tr (S.L.); yozkay@anadolu.edu.tr (Y.Ö.); zakaplan@anadolu.edu.tr (Z.A.K.); 2 Doping and Narcotic Compounds Analysis Laboratory, Faculty of Pharmacy, Anadolu University, 26470 Eskişehir, Turkey

**Keywords:** thiosemicarbazone, benzofuran, benzothiophene, *h*MAO enzymes, molecular docking

## Abstract

MAO-B inhibitors are frequently used in the treatment of neurodegenerative diseases such as Parkinson’s and Alzheimer’s. Due to the limited number of compounds available in this field, there is a need to develop new compounds. In the recent works, it was shown that various thiosemicarbazone derivatives show *h*MAO inhibitory activity in the range of micromolar concentration. It is thought that benzofuran and benzothiophene structures may mimic structures such as indane and indanone, which are frequently found in the structures of such inhibitors. Based on this view, new benzofuran/benzothiophene and thiosemicarbazone hybrid compounds were synthesized, characterized and screened for their *h*MAO-A and *h*MAO-B inhibitory activity by an in vitro fluorometric method. The compounds including methoxyethyl substituent (**2b** and **2h**) were found to be the most effective agents in the series against MAO-B enzyme with the IC_50_ value of 0.042 ± 0.002 µM and 0.056 ± 0.002 µM, respectively. The mechanism of *h*MAO-B inhibition of compounds **2b** and **2h** was investigated by Lineweaver–Burk graphics. Compounds **2b** and **2h** were reversible and non-competitive inhibitors with similar inhibition features as the substrates. The K_i_ values of compounds **2b** and **2h** were calculated as 0.035 µM and 0.046 µM, respectively, with the help of secondary plots. The docking study of compound **2b** and **2h** revealed that there is a strong interaction between the active sites of *h*MAO-B and analyzed compound.

## 1. Introduction

Neuropsychiatric diseases such as Parkinson’s disease (PD), Alzheimer’s disease (AD) and depression, which have all become a serious health problem in the world, still do not have an effective treatment for these diseases, mainly due to insufficient understanding of the multi-component pathogenesis [1,2]. Among the well-studied etiologies, the irregular expression of mitochondrial enzyme monoamine oxidases (MAO, EC 1.4.3.4) has been recognized as a main cause. This depends on the fact that a neurodegenerative disorder is the result of unnecessary monoamine metabolites produced by the overexpression of MAO. The neurotransmitter-catabolizing MAOs are classified into the A and B isoforms [3,4,5].

The MAO enzyme, which catalyzes the oxidative deamination of various monoamines, both endogenous and exogenous, has important roles in metabolizing released neurotransmitters and detoxifying a wide variety of endogenous and exogenous amines. Mainly involved in the breakdown of norepinephrine, melatonin, serotonin, and epinephrine, MAO-A is expressed almost everywhere in the human body. MAO-B, which degrades benzylamine and phenethylamine, is highly expressed in the central nervous system (CNS). Tryptamine, tyramine, and dopamine can be metabolized by both isoforms with individual metabolic activity for each substrate [6,7,8]. In light of all this information, the development of new MAO inhibitors (MAOIs) for the treatment of numerous neurological and psychiatric disorders represents a very important and useful approach [9,10]. The disadvantages of non-reversible or non-selective inhibitors have seriously reduced the use of these inhibitors in therapy. Selective MAO-A inhibitors are used in the treatment of mental disorders and have found use in therapy as antidepressants and anxiolytics [11,12], while selective MAO-B inhibitors are used as adjuvants for the treatment of Alzheimer’s disease, Parkinson’s disease, and Huntington’s disease [13,14,15,16]. The design of such reversible and selective inhibitors of MAO-A and MAO-B to identify suitable drug candidates for the treatment of neurological diseases has become a highly studied area [17].

Some MAO-B inhibitors have been evaluated in clinical trials for the treatment of AD (e.g., Rasagiline, Lazabemide, Safinamide, and Selegiline, and these have shown positive effects on memory patterns and progression of disease [10,17,18,19]. A clinical trial with the selective MAO-A inhibitors, Moclobemide and Clorgyline, has shown improvement in symptoms of depression and cognition in elderly patients with memory impairment [20,21].

A literature survey has revealed many important observations about structure-activity relationships of MAO inhibitors [3,15,22,23,24,25,26,27,28,29].

Our understanding of where and how substrates (or inhibitors) enter the active sites of MAO-A and MAO-B is invaluable for rational use of design strategies. The structural data indicate that the ligands MAO-A and MAO-B follow the same binding pathways as the target protein [30]. In addition, it was also revealed that the cavity-shaping loop (residues 210–216 in MAO-A) took on a helical conformation, which was conserved in all MAO-A structures except in hMAO-A complexed with Clorgyline. Generally, the MAO-B isoform, which is of a hydrophobic nature, contains a small, highly conserved hydrophilic region in the inlet cavity. [31]. The cavity may contain a small inhibitor (e.g., Isatin) or a cavity-filling ligand (e.g., Rasagiline [32]) reliant on the conformation of the Ile199 gating residue. It may contain a small inhibitor because the active site is limited to a smaller cavity separated from an entrance cavity space. On the contrary, binding of inhibitors, such as Rasagiline or Selegiline [30] can induce a mid-span cavity type because these ligands are large enough to push the Ile199 gating residue into the open conformation. It should be noted that this cavity plasticity in MAO-B is probably determined by the subtle conformational changes [3].

**Figure 1:**
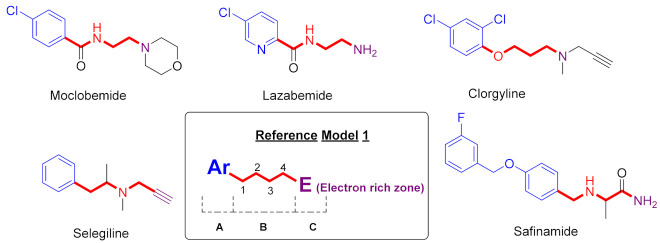
MAO-inhibitors (Moclobemide, Selegiline, Clorgyline, Lazabemide, and Safinamide) and their common structure features.

Studies from some research groups show that MAO inhibitors Moclobemide, Selegiline, Clorgyline, Lazabemide, and Safinamide have identified some common structural features attributed to the presence of (A) an aryl ring, (B) a chain to four atoms containing nitrogen, (C) an electron-rich area due to an amino group or a triple bond (Figure 1) [24,25,27].

**Figure 2:**
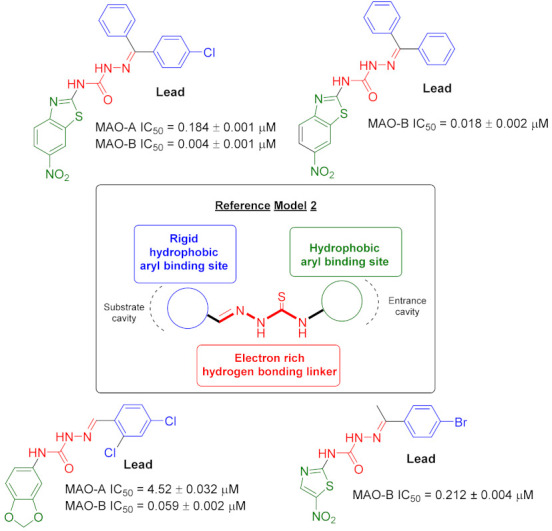
Some novel MAO inhibitor agents and reference model of target compounds.

By analysis of experimental and computer data, the following significant pharmacophoric elements influencing the binding affinity and potency of some major MAO-B inhibitors (semicarbazone derivatives) were identified: (1) A hydrophobic aromatic ring at the terminus of semicarbazone is essential for binding to the substrate cavity of the MAO-B active site; (2) Another hydrophobic moiety is crucial for efficient binding and stabilization in the MAO-B inlet cavity space; (3) A flexible linker having hydrogen bonding regions is also essential to guide the optimal orientation of the two hydrophobic aryl residues in their respective binding pockets within the active site of MAO-B (Figure 2) [15,22,28].

**Figure 3:**
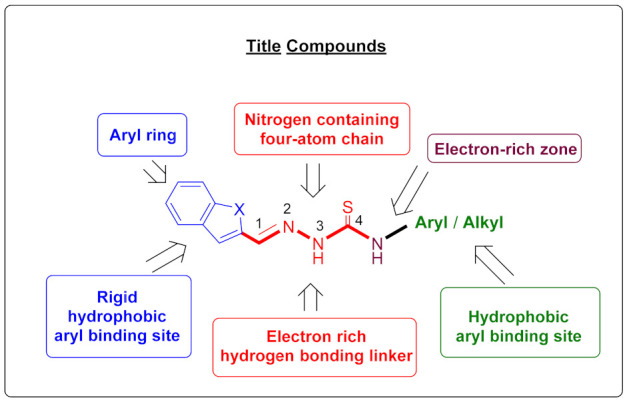
Design of obtained compounds (**2a**–**2l**).

The data from these two SAR studies described above encouraged us to design new compounds. In this study, we report the synthesis and in vitro evaluation of a novel series of thiosemicarbazone derivatives which include both reference models on the same structure. In vitro evaluation includes the inhibition of the MAO-A, MAO-B enzymes in direct assays. Molecular docking and molecular simulation (MD simulation) studies were also performed to elucidate the interaction of potent compounds with the active site of enzymes (Figure 3).

## 2. Results and Discussion

### 2.1. Chemistry

**Scheme 1:**
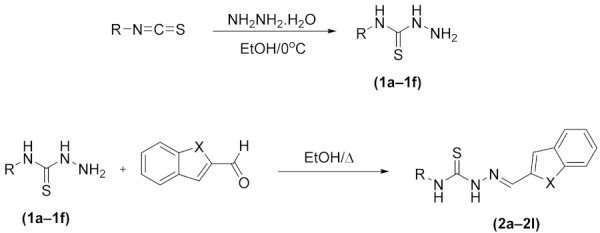
The synthetic route for target compounds (**2a**–**2l**).

The compounds **2a**–**2l** were obtained as presented in Scheme 1. Firstly, thiosemicarbazide derivatives (**1a**–**1f**) were obtained by means of reaction between isothiocyanate derivatives and hydrazine hydrate using an ice bath. Finally, the resulting thiosemicarbazide derivatives (**1a**–**1f**) and the appropriate benzaldehydes were reacted to synthesize the target compounds. ^1^H-NMR, ^13^C-NMR, APCI-MS, and HRMS are used as spectroscopic methods (supplementary material).

In benzofuran containing compounds (**2a**–**2f**), benzofuran was recorded between 7.26 ppm and 7.30 ppm as doublet of triplet with 1H integration value. Other benzofuran hydrogens were recorded between 7.35 ppm and 7.42 ppm as multiplet with 2H integration value, 7.52 ppm and 7.61 ppm as double doublet with 1H integration value, 7.68 ppm and 7.71 ppm as double doublet with 1H integration value. In benzothiophene containing compounds (**2g**–**2l**), benzothiophene was recorded between 7.36 ppm and 7.43 ppm as multiplet with 2H integration value. Other benzothiophene hydrogens were recorded between 7.75 ppm and 7.77 ppm as singlet with 1H integration value, 7.82 ppm and 7.86 ppm as multiplet with 1H integration value, and 7.93 ppm and 7.97 ppm as multiplet with 1H integration value. Methylene protons were recorded between 8.10 ppm and 8.49 ppm as singlet. Additionally, APCI-MS and HRMS results were also well-suited with theoretical m/z values. Moreover, in the purity studies using the PDA detector, it is seen that all of the compounds have a purity value above 95%.

### 2.2. MAO Inhibition

The inhibition power on MAO isoenzymes of the synthesized compounds was evaluated by using the in vitro fluorometric method declared previously by our research group [33,34,35,36,37,38,39]. The results of the first step of enzyme activity assay were given in Table 1; that of the second step was presented in Table 2. The second step was performed with further concentrations (by serial dilutions ranging from 10^−5^ M to 10^−9^ M) of the reference drug and selected derivatives that showed more than 50% inhibitory activity at 10^−4^ M concentration. Therefore, the half maximal inhibitory concentration (IC_50_) values of the selected compounds and reference inhibitor could be calculated, and these results are given in Table 2.

It was understood by analyzing Table 1 that some of the synthesized compounds in the series showed remarkable inhibition potency at 10^−3^ M concentration; however, none of them displayed significant inhibitory activity at 10^−4^ M concentration against MAO-A enzyme. It was seen that all obtained derivatives had higher inhibition rates on MAO-B enzyme than MAO-A enzyme. So, it was concluded that all synthesized compounds showed selective MAO-B enzyme inhibitory activity. All compounds displayed more than 50% inhibitory activity at 10^−3^ M concentration, while only compounds **2b** and **2h** had more than 50% inhibitory activity at 10^−4^ M concentration for MAO-B enzyme, and thus, the second step of enzyme inhibition assay was carried out by using further dilutions of these compounds. Compounds **2b** and **2h** were found to be the most effective agents in the series with the IC_50_ value of 0.042 ± 0.002 µM and 0.056 ± 0.002 µM, respectively. Furthermore, it was noteworthy that these compounds performed a very similar degree of inhibition to reference drug Selegiline (IC_50_ value = 0.037 ± 0.001 µM). It is noteworthy that both compounds with high activity values carry methoxyethyl residues. It was thought that this residue contributed positively to the activity. When these two derivatives are compared, it is seen that the compound carrying the benzofuran ring is more active.

### 2.3. Kinetic Studies of Enzyme Inhibition

**Figure 4:**
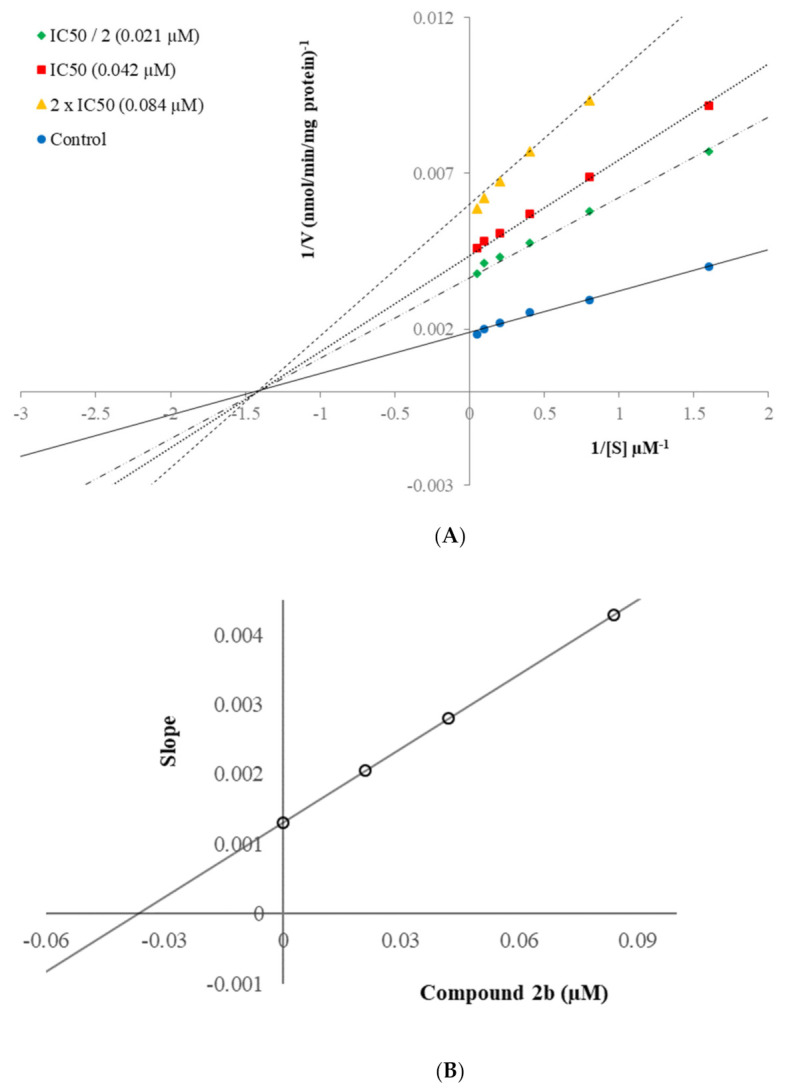
(**A**) Lineweaver–Burk plots for the inhibition of hMAO-B by compound **2b**. [S], substrate concentration (µM); V, reaction velocity (nmol/min/mg protein). Inhibitor concentrations are shown at the left; (**B**) Secondary plot for the calculation of the steady-state inhibition constant (K_i_) of compound **2b**. K_i_ was calculated as 0.035 µM.

**Figure 5:**
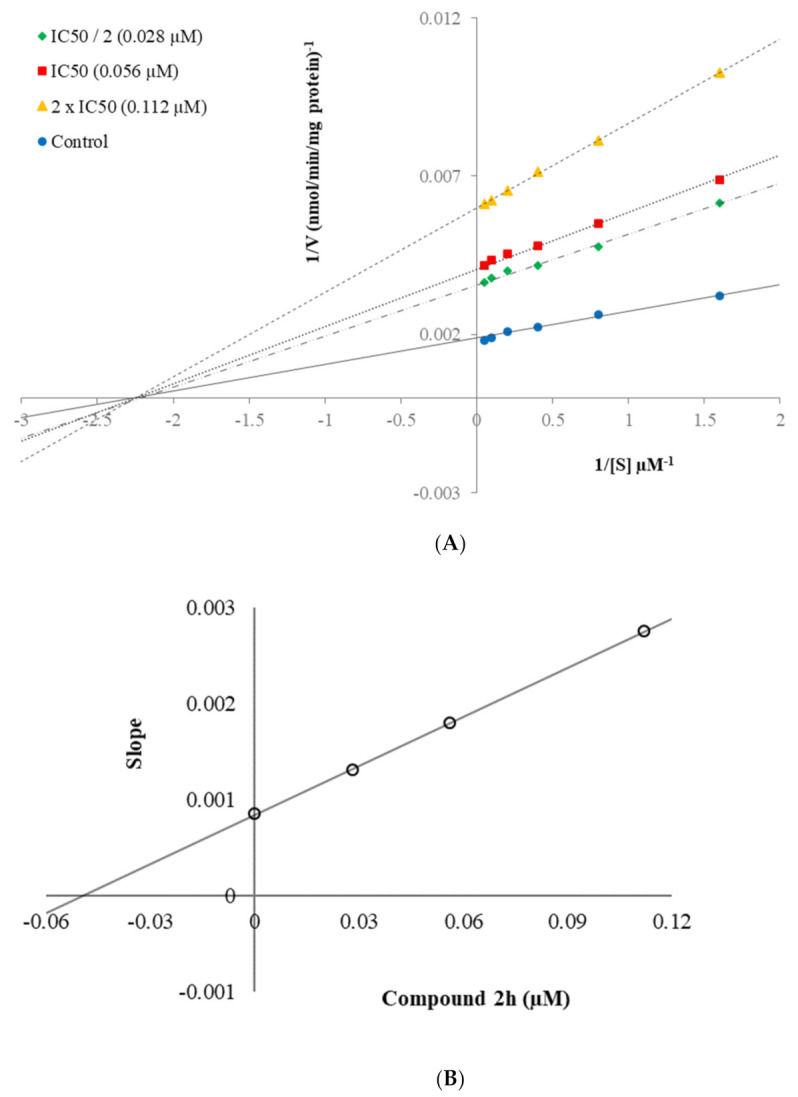
(**A**) Lineweaver–Burk plots for the inhibition of hMAO-B by compound **2h**. [S], substrate concentration (µM); V, reaction velocity (nmol/min/mg protein). Inhibitor concentrations are shown at the left. (**B**) Secondary plot for the calculation of the steady-state inhibition constant (K_i_) of compound **2h**. K_i_ was calculated as 0.046 µM.

Enzyme kinetic studies help to determine the inhibitory character of the relevant compound on the enzyme as irreversible or reversible inhibition, and to evaluate the nature of the inhibition of the relevant compound. The linear Lineweaver–Burk plots are the most commonly used method for this purpose. Compounds **2b** and **2h** which were found to be the most effective derivatives in the series, were included in the enzyme kinetic assay to evaluate the inhibition types of them on MAO-B enzyme. Enzyme kinetic assay was applied in the same way as previously mentioned [33,34,35,36,37,38,39]. The IC_50_/2, IC_50_, and 2(IC_50_) were used as the concentrations of these compounds. The rate curves of substrates were reported in the absence and presence of these compounds. In all cases, initial velocity measurements were collected at various substrate (tyramine) concentrations ranging from 20 µM to 0.625 µM. Thus, linear Lineweaver–Burk graphs of compounds **2b** and **2h** were formed as in Figure 4A and Figure 5A, respectively. The secondary plots of slope (K_m_/V_max_) versus varying concentrations (0, IC_50_/2, IC_50_, and 2(IC_50_)) (Figure 4B and Figure 5B) were formed to measure the K_i_ values of these compounds.

According to Lineweaver–Burk plots, reversible and irreversible inhibition type constitute two general categories. Mixed-type, uncompetitive, competitive, and non-competitive inhibition types are included in the reversible inhibition [33,34,35,36,37,38,39]. As seen in the Lineweaver–Burk graphs of compounds **2b** and **2h** (Figure 4 and Figure 5), the lines were intersected on the x-axis, and their slopes and y-intercepts were different. This observation indicated that compounds **2b** and **2h** were reversible and non-competitive inhibitors with similar inhibition features as the substrates. The K_i_ values of compounds **2b** and **2h** were calculated as 0.035 µM and 0.046 µM, respectively, with the help of secondary plots.

### 2.4. Cytotoxicity Assay

Compounds **2b** and **2h** exhibited potent MAO-B inhibition profile and were further tested for toxicity using the MTT assay in the NIH/3T3 cell line; the IC_50_ value of this compound is shown in Table 3. Compound **2b** and **2h** showed an IC_50_ value of 129.631 µM and 81.266 µM against NIH/3T3 cells, which was significantly higher than its IC_50_ value (0.042 µM and 0.056 µM, respectively) against MAO-B. Consequently, compounds **2b** and **2h** were found to be non-cytotoxic at its effective concentration against MAO-B. This result further increases the biological importance of this compound.

### 2.5. Molecular Docking

Docking studies were carried out on using the X-ray crystal structure of MAO-B enzyme (PDB Code: 2V5Z) [40] in order to determine the possible interactions between the enzyme active site and compounds **2b** and **2h** which were the most active derivatives among the synthesized compounds whose enzyme activities were evaluated.

**Figure 6:**
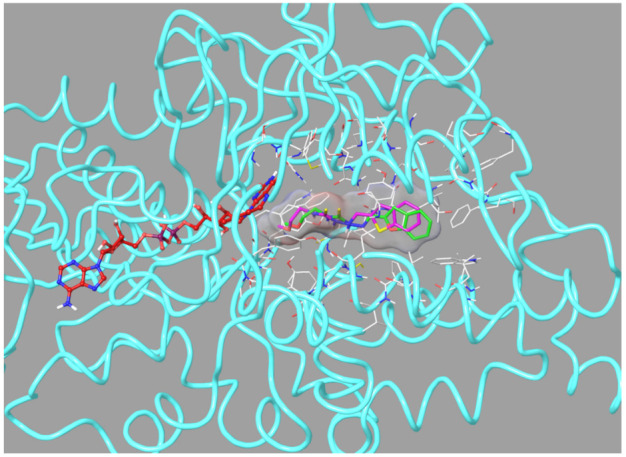
The superimposition pose of compounds **2b** and **2h** in the active region of hMAO-B (PDB ID: 2V5Z). The important residues in the active site and compounds **2b** and **2h** are presented by tube model and colored with white, green, and pink, respectively.

**Figure 7:**
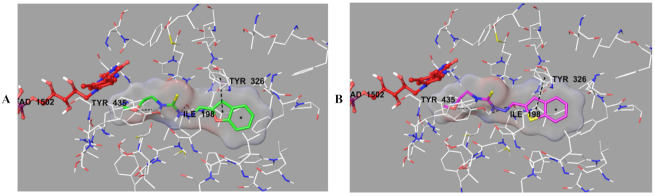
The three-dimensional interacting modes of compounds **2b** (**A**) and **2h** (**B**) in the active region of *h*MAO-B. The inhibitors and the important residues in the active site of the enzyme are presented by tube model. The FAD molecule is colored red with ball-stick model.

**Figure 8:**
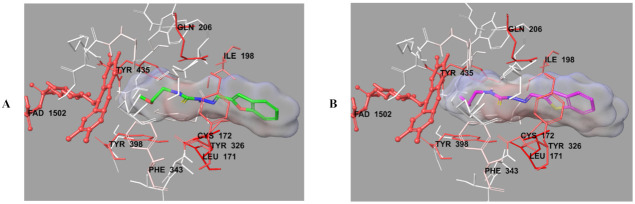
The van der Waals interactions of compounds **2b** (**A**) and **2h** (**B**) with active region of *h*MAO-B. The active ligands has a lot of favorable van der Waals interactions (red and pink).

**Figure 9:**
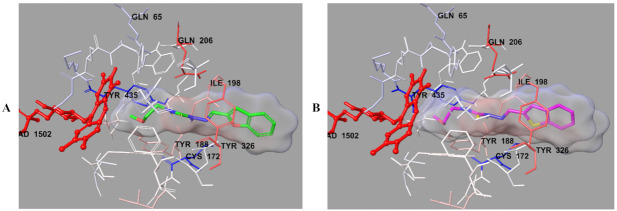
The electrostatic interactions of compounds **2b** (**A**) and **2h** (**B**) with active region of *h*MAO-B. The residues are colored (blue, red, and pink) according to the distance from ligand by Per-Residue Interaction panel.

The docking poses of these compounds were given in Figure 6, Figure 7, Figure 8 and Figure 9. When analyzed, especially Figure 6, it was understood that compounds **2b** and **2h** were positioned in a very similar position at the active site of MAO-B enzyme. These compounds showed the same interactions including a π-π interaction and two hydrogen bond formations (Figure 7). The π-π interaction was observed between benzofuran/benzothiophene ring and the phenyl of Tyr326. The hydrazide moiety was essential for polar interactions. The amino of hydrazide formed a hydrogen bond with the carbonyl of Ile198. The last hydrogen bond was detected between the oxygen atom of methoxyethyl group near to thioamide and the hydroxyl of Try435. This interaction of this residue in the enzyme active site shows the contribution of this residue to the activity.

Actually, the last mentioned interaction was an indicator to explain the difference in the enzyme inhibition profiles of the synthesized compounds and why compounds **2b** and **2h** were found to be the most effective agents. Compounds **2b** and **2h** had methoxyethyl group different from the other derivatives in the series. It was thought that the substituents, which were capable of the formation of a hydrogen bond, such as the methoxyethyl moiety, strongly contributed to binding to the active site of the enzyme. Thus, this situation could explain why compounds **2b** and **2h** exhibited stronger inhibition profiles than the other compounds, because only these compounds had such mentioned substituents, which were different from the other compounds in the series.

In order to analyze the contribution of van der Waals and electrostatic interactions in binding to the enzyme active site, docking studies were carried out, detailed by using Glide according to Per-Residue Interaction panel. Figure 8 and Figure 9 presented van der Waals and electrostatic interactions of compounds **2b** and **2h**. It was seen that these compounds had favorable van der Waals interactions (displayed with pink and red colors as described in the user guide of Glide [41]) with Leu171, Cys172, Ile198, Gln206, Tyr326, Phe343, Tyr398, Tyr435, and FAD molecule. Similarly, promising electrostatic contributions of these compounds were determined with Gln65, Cys172, Tyr188, Ile198, Gln206, Tyr326, Tyr435, and FAD molecule.

## 3. Materials and Methods

### 3.1. Chemistry

All reagents were purchased from commercial suppliers and were used without further purification. Melting points (M.p.) were determined on Mettler Toledo-MP90 Melting Point System and were uncorrected. ^1^H NMR and ^13^C NMR spectra was recorded on a Bruker Fourier 300 (Bruker Bioscience, Billerica, MA, USA), respectively, in DMSO-*d*_6_ (Bruker, Bioscience, Billerica, MA, USA), respectively. Mass spectra were recorded on an APCI-MS (Advion, New York, NY, USA) by using the at atmospheric-pressure chemical ionization (APCI) method. Mass spectra were recorded on an LCMS-IT-TOF (Shimadzu, Kyoto, Japan) equipped with a PDA detector (Supplementary Material). Silica gel 60 F254 with thin-layer chromatography (Merck KGaA, Darmstadt, Germany) was used to check the purity of compounds.

General Procedure for the Synthesis of the Compounds

General procedure for the synthesis of thiosemicarbazide derivatives (**1a**–**1f**)

Isothiocyanate derivatives (0.007 mol) were dissolved in EtOH (10 mL). Excess of hydrazine hydrate was added in reaction mixture as portions at ice-bath. After completion of the reaction, the mixture was poured into ice-water (50 mL), precipitated product was filtered, washed with cooled EtOH, and dried. Experimental melting degrees are as follows, respectively: 83.0–84.0 °C, 88.1–89.3 °C, 73.9–74.1 °C, 140.0–142.2 °C, 151.0–152.3 °C, 131.3–133.5 °C [42,43,44,45,46,47].

General procedures of target compounds (**2a**–**2l**)

Corresponding aldehyde derivatives (0.002 mol) and appropriate isothiocyanate derivative (0.002 mol) in EtOH (25 mL) were refluxed for 8 h. The mixture was cooled in an ice-bath, precipitated product was filtered, dried, and recrystallized from EtOH.

*2-(Benzofuran-2-ylmethylene)-N-ethylhydrazine-1-carbothioamide* (**2a**)

Yield: 85%, M.P.: 179.9–182.1 °C. Purity: 99.9%. ^1^H-NMR (300 MHz, DMSO-*d*_6_): δ = 1.15 (3H, t, *J* = 7.1 Hz, -NHCH_2_CH_3_), 3.60 (2H, p, *J* = 6.8 Hz, -NHCH_2_CH_3_), 7.29 (1H, td, *J*_1_ = 0.9 Hz, *J*_2_ = 7.7 Hz, Benzofuran), 7.36–7.41 (2H, m, Benzofuran), 7.60 (1H, dd, *J*_1_ = 0.8 Hz, *J*_2_ = 8.2 Hz, Benzofuran), 7.69 (1H, dd, *J*_1_ = 0.7 Hz, *J*_2_ = 7.1 Hz, Benzofuran), 8.10 (1H, s, -CH = N-), 8.42 (1H, t, *J* = 5.8 Hz, -NHCH_2_CH_3_), 11.65 (1H, s, -NH). ^13^C-NMR (75 MHz, DMSO-*d*_6_): δ = 14.96, 38.90, 109.86, 111.78, 122.24, 126.46, 128.39, 132.21, 151.72, 155.10, 177.06. APCI-MS [M + H]^+^: 248.3. HRMS (*m*/*z*): [M + H]^+^ calcd. for C_12_H_13_N_3_OS: 248.0852; found: 248.0860.

*2-(Benzofuran-2-ylmethylene)-N-(2-methoxyethyl)hydrazine-1-carbothioamide* (**2b**)

Yield: 80%, M.P.: 147.5–148.8 °C. Purity: 100.0%. ^1^H-NMR (300 MHz, DMSO-*d*_6_): δ = 3.29 (3H, s, NHCH_2_CH_3_OCH_3_), 3.53 (3H, t, *J* = 5.8 Hz, -NHCH_2_CH_2_OCH_3_), 3.76 (2H, q, *J* = 5.8 Hz, -NHCH_2_CH_3_OCH_3_), 7.29 (1H, td, *J*_1_ = 0.8 Hz, *J*_2_ = 7.7 Hz, Benzofuran), 7.36–7.42 (2H, m, Benzofuran), 7.61 (1H, dd, *J*_1_ = 0.8 Hz, *J*_2_ = 8.2 Hz, Benzofuran), 7.69 (1H, dd, *J*_1_ = 0.6 Hz, *J*_2_ = 7.7 Hz, Benzofuran), 8.12 (1H, s, -CH=N-), 8.30 (1H, t, *J* = 5.8 Hz, -NHCH_2_CH_3_OCH_3_), 11.78 (1H, s, -NH). ^13^C-NMR (75 MHz, DMSO-*d*_6_): δ = 43.48, 58.45, 70.44, 110.24, 110.28, 111.80, 122.29, 124.04, 126.56, 128.35, 132.52, 132.56, 151.55, 155.13, 177.56. APCI-MS [M + H]^+^: 278.3. HRMS (*m*/*z*): [M + H]^+^ calcd. for C_13_H_15_N_3_O_2_S: 278.0958; found: 278.0953.

*2-(Benzofuran-2-ylmethylene)-N-butylhydrazine-1-carbothioamide* (**2c**)

Yield: 79%, M.P.: 120.4–121.2 °C. Purity: 100.0%. ^1^H-NMR (300 MHz, DMSO-*d*_6_): δ = 0.91 (3H, t, *J* = 7.3 Hz, -CH_3_), 1.25–1.37 (2H, m, -CH_2_-), 1.58 (2H, p, *J* = 7.3 Hz, -CH_2_-), 3.54–3.56 (2H, m, -CH_2_-), 7.26 (1H, td, *J*_1_ = 0.8 Hz, *J*_2_ = 7.7 Hz, Benzofuran), 7.36–7.41 (2H, m, Benzofuran), 7.60 (1H, dd, *J*_1_ = 0.8 Hz, *J*_2_ = 8.2 Hz, Benzofuran), 7.69 (1H, d, *J* = 7.2 Hz, Benzofuran), 8.10 (1H, s, -CH=N-), 8.39 (1H, t, *J* = 5.9 Hz, -NH), 11.65 (1H, s, -NH). ^13^C-NMR (75 MHz, DMSO-*d*_6_): δ = 14.28, 20.04, 31.40, 43.75, 109.79, 111.78, 122.24, 124.02, 126.45, 128.39, 132.21, 151.73, 155.10, 177.26. APCI-MS [M + H]^+^: 276.3. HRMS (*m*/*z*): [M + H]^+^ calcd. for C_14_H_17_N_3_OS: 276.1165; found: 276.1158.

*2-(Benzofuran-2-ylmethylene)-N-cyclohexylhydrazine-1-carbothioamide* (**2d**)

Yield: 85%, M.P.: 191.2–192.9 °C. Purity: 99.6%. ^1^H-NMR (300 MHz, DMSO-*d*_6_): δ = 1.16–1.46 (5H, m, cyclohexyl), 1.59–1.92 (5H, m, cyclohexyl), 4.16–4.19 (1H, m, cyclohexyl), 7.28 (1H, td, *J*_1_ = 0.8 Hz, *J*_2_ = 7.7 Hz, Benzofuran), 7.36–7.41 (2H, m, Benzofuran), 7.61 (1H, dd, *J*_1_ = 0.6 Hz, *J*_2_ = 8.2 Hz, Benzofuran), 7.68 (1H, dd, *J*_1_ = 0.6 Hz, *J*_2_ = 8.2 Hz, Benzofuran), 7.92 (1H, d, *J* = 8.5 Hz, -NH), 8.11 (1H, s, -CH=N-), 11.66 (1H, s, -NH). ^13^C-NMR (75 MHz, DMSO-*d*_6_): δ = 25.37, 25.55, 32.23, 52.22, 109.92, 111.85, 122.24, 124.03, 126.48, 128.37, 132.38, 151.63, 155.13, 176.11. APCI-MS [M + H]^+^: 302.4. HRMS (*m*/*z*): [M + H]^+^ calcd. for C_16_H_19_N_3_OS: 302.1322; found: 302.1310 [48].

*2-(Benzofuran-2-ylmethylene)-N-phenylhydrazine-1-carbothioamide* (**2e**)

Yield: 88%, M.P.: 205.2–206.8 °C. Purity: 100.0%. ^1^H-NMR (300 MHz, DMSO-*d*_6_): δ = 7.21 (1H, tt, *J*_1_ = 1.1 Hz, *J*_2_ = 7.4 Hz, Benzofuran), 7.30 (1H, td, *J*_1_ = 0.8 Hz, *J*_2_ = 7.7 Hz, Benzofuran), 7.35–7.42 (3H, m, Aryl-H), 7.52 (1H, d, *J* = 0.8 Hz, Benzofuran), 7.59–7.62 (3H, m, Aryl-H), 7.70 (1H, dd, *J*_1_ = 0.8 Hz, *J*_2_ = 7.1 Hz, Benzofuran), 8.21 (1H, s, -CH=N-), 10.04 (1H, s, -NH), 12.06 (1H, s, -NH). ^13^C-NMR (75 MHz, DMSO-*d_6_*): δ = 109.86, 111.86, 122.33, 124.07, 125.81, 125.92, 126.56, 128.38, 128.61, 133.01, 139.34, 151.69, 155.20, 176.42. APCI-MS [M + H]^+^: 296.3. HRMS (*m*/*z*): [M + H]^+^ calcd. for C_16_H_13_N_3_OS: 296.0852; found: 296.0864 [48].

*2-(Benzofuran-2-ylmethylene)-N-(2-chlorophenyl)hydrazine-1-carbothioamide* (**2f**)

Yield: 89%, M.P.: 203.6–204.9 °C. Purity: 100.0%. ^1^H-NMR (300 MHz, DMSO-*d*_6_): δ = 7.27–7.34 (2H, m, Aryl-H), 7.36–7.43 (2H, m, Aryl-H), 7.50 (1H, d, *J* = 0.7 Hz, Benzofuran), 7.56 (1H, dd, *J*_1_ = 1.6 Hz, *J*_2_ = 7.8 Hz, 1,2-Disubstituebenzene), 7.61 (1H, dd, *J*_1_ = 0.7 Hz, *J*_2_ = 8.1 Hz, Benzofuran), 7.71 (1H, d, *J* = 7.2 Hz, Benzofuran), 7.75 (1H, dd, *J*_1_ = 1.6 Hz, *J*_2_ = 7.8 Hz, 1,2-Disubstituebenzene), 8.21 (1H, s, -CH=N-), 10.02 (1H, s, -NH), 12.22 (1H, s, -NH). ^13^C-NMR (75 MHz, DMSO-*d*_6_): δ = 110.15, 111.84, 122.39, 124.09, 126.64, 127.68, 128.27, 128.35, 129.80, 130.73, 133.26, 136.76, 151.56, 155.21, 177.04. APCI-MS [M + H]^+^: 330.3. HRMS (*m*/*z*): [M + H]^+^ calcd. for C_16_H_12_N_3_OSCl: 330.0462; found: 330.0468.

*2-(Benzo[b]thiophen-2-ylmethylene)-N-ethylhydrazine-1-carbothioamide* (**2g**)

Yield: 77%, M.P.: 198.9–201.0 °C. Purity: 99.8%. ^1^H-NMR (300 MHz, DMSO-*d*_6_): δ = 1.16 (3H, t, *J* = 7.1 Hz, -NHCH_2_CH_3_), 3.60 (2H, p, *J* = 6.8 Hz, -NHCH_2_CH_3_), 7.36–7.43 (2H, m, Benzothiophen), 7.75 (1H, s, Benzothiophen), 7.82–7.85 (1H, m, Benzothiophen), 7.93–7.96 (1H, m, Benzothiophen), 8.23 (1H, t, *J* = 5.8 Hz, -NHCH_2_CH_3_), 8.36 (1H, s, -CH=N-), 11.61 (1H, s, -NH). ^13^C-NMR (75 MHz, DMSO-*d*_6_): δ = 14.96, 38.91, 123.02, 124.71, 125.29, 126.39, 127.93, 127.97, 137.76, 139.67, 139.79, 176.91. APCI-MS [M + H]^+^: 264.2. HRMS (*m*/*z*): [M + H]^+^ calcd. for C_12_H_13_N_3_S_2_: 264.0624; found: 264.0614 [49].

*2-(Benzo[b]thiophen-2-ylmethylene)-N-(2-methoxyethyl)hydrazine-1-carbothioamide* (**2h**)

Yield: 78%, M.P.: 164.9–166.2 °C. Purity: 100.0%. ^1^H-NMR (300 MHz, DMSO-*d*_6_): δ = 3.30 (3H, s, NHCH_2_CH_3_OCH_3_), 3.53 (3H, t, *J* = 5.8 Hz, -NHCH_2_CH_2_OCH_3_), 3.75 (2H, q, *J* = 5.7 Hz, -NHCH_2_CH_3_OCH_3_), 7.38–7.41 (2H, m, Benzothiophen), 7.77 (1H, s, Benzothiophen), 7.83–7.86 (1H, m, Benzothiophen), 7.94–7.97 (1H, m, Benzothiophen), 8.12 (1H, t, *J* = 5.6 Hz, -NHCH_2_CH_3_), 8.38 (1H, s, -CH=N-), 11.76 (1H, s, -NH). ^13^C-NMR (75 MHz, DMSO-*d*_6_): δ = 43.50, 58.52, 70.44, 123.06, 124.77, 125.33, 126.48, 128.26, 128.30, 138.13, 139.51, 139.78, 177.36. APCI-MS [M + H]^+^: 294.3. HRMS (*m*/*z*): [M + H]^+^ calcd. for C_13_H_15_N_3_OS_2_: 294.0729; found: 294.0715.

*2-(Benzo[b]thiophen-2-ylmethylene)-N-butylhydrazine-1-carbothioamide* (**2i**)

Yield: 80%, M.P.: 168.9–170.7 °C. Purity: 100.0%. ^1^H-NMR (300 MHz, DMSO-*d*_6_): δ = 0.92 (3H, t, *J* = 7.3 Hz, -CH_3_), 1.28–1.36 (2H, m, -CH_2_-), 1.57 (2H, p, *J* = 7.3 Hz, -CH_2_-), 3.57 (2H, q, *J* = 6.8 Hz, -CH_2_-), 7.37–7.40 (2H, m, Benzothiophen), 7.75 (1H, s, Benzothiophen), 7.82–7.85 (1H, m, Benzothiophen), 7.93–7.96 (1H, m, Benzothiophen), 8.18 (1H, t, *J* = 5.8 Hz, -NH), 8.36 (1H, s, -CH=N-), 11.63 (1H, s, -NH). ^13^C-NMR (75 MHz, DMSO-*d*_6_): δ = 14.27, 20.06, 31.38, 43.75, 123.02, 124.71, 125.28, 126.38, 127.92, 127.96, 137.73, 139.69, 139.80, 177.10. APCI-MS [M + H]^+^: 292.3. HRMS (*m*/*z*): [M + H]^+^ calcd. for C_14_H_17_N_3_S_2_: 292.0937; found: 292.0949.

*2-(Benzo[b]thiophen-2-ylmethylene)-N-cyclohexylhydrazine-1-carbothioamide* (**2j**)

Yield: 77%, M.P.: 213.7–215.1 °C. Purity: 100.0%. ^1^H-NMR (300 MHz, DMSO-*d*_6_): δ = 1.18–1.44 (5H, m, cyclohexyl), 1.57–1.92 (5H, m, cyclohexyl), 4.15–4.18 (1H, m, cyclohexyl), 7.38–7.41 (2H, m, Benzothiophen), 7.69 (1H, t, *J* = 8.3 Hz, -NH), 7.77 (1H, s, Benzothiophen), 7.83–7.86 (1H, m, Benzothiophen), 7.94–7.97 (1H, m, Benzothiophen), 8.36 (1H, s, -CH=N-), 11.67 (1H, s, -NH). ^13^C-NMR (75 MHz, DMSO-*d*_6_): δ = 25.09, 25.55, 32.18, 52.74, 123.07, 124.76, 125.33, 126.46, 128.08, 128.12, 137.83, 139.56, 139.82, 175.97. APCI-MS [M + H]^+^: 318.3. HRMS (*m*/*z*): [M + H]^+^ calcd. for C_16_H_19_N_3_S_2_: 318.1093; found: 318.1098.

*2-(Benzo[b]thiophen-2-ylmethylene)-N-phenylhydrazine-1-carbothioamide* (**2k**)

Yield: 82%, M.P.: 213.9–216.0 °C. Purity: 98.4%. ^1^H-NMR (300 MHz, DMSO-*d*_6_): δ = 7.19–7.24 (1H, m, Aryl-H), 7.35–7.44 (4H, m, Aryl-H), 7.60 (2H, d, *J* = 7.7 Hz, 1,4-Disubstituebenzene), 7.85 (1H, s, Benzothiophen), 7.86–7.88 (1H, m, Benzothiophen), 7.94–7.97 (1H, m, Benzothiophen), 8.47 (1H, s, -CH=N-), 9.88 (1H, s, -NH), 12.02 (1H, s, -NH). ^13^C-NMR (75 MHz, DMSO-*d*_6_): δ = 123.08, 124.82, 125.32, 125.80, 126.53, 128.58, 128.64, 128.64, 138.65, 138.68, 139.38, 139.43, 139.75, 140.04, 176.15. APCI-MS [M + H]^+^: 312.3. HRMS (*m*/*z*): [M + H]^+^ calcd. for C_16_H_13_N_3_S_2_: 312.0624; found: 312.0611.

*2-(Benzo[b]thiophen-2-ylmethylene)-N-(2-chlorophenyl)hydrazine-1-carbothioamide* (**2l**)

Yield: 84%, M.P.: 212.6–214.4 °C. Purity: 96.0%. ^1^H-NMR (300 MHz, DMSO-*d*_6_): δ = 7.26–7.31 (2H, m, Aryl-H), 7.36–7.45 (3H, m, Aryl-H), 7.57 (1H, dd, *J*_1_ = 1.5 Hz, *J*_2_ = 7.9 Hz, 1,2-Disubstituebenzene), 7.86-.78 (2H, m, Aryl-H), 7.96–7.99 (1H, m, Benzothiophen), 8.09 (1H, dd, *J*_1_ = 1.6 Hz, *J*_2_ = 8.0 Hz, 1,2-Disubstituebenzene), 8.49 (1H, s, -CH=N-), 9.93 (1H, s, -NH), 12.26 (1H, s, -NH). ^13^C-NMR (75 MHz, DMSO-*d*_6_): δ = 123.16, 124.91, 125.38, 126.67, 127.60, 127.65, 127.93, 129.01, 129.11, 129.76, 136.40, 138.99, 139.27, 139.75, 140.00, 175.99. APCI-MS [M + H]^+^: 346.3. HRMS (*m*/*z*): [M + H]^+^ calcd. for C_16_H_12_N_3_S_2_Cl: 345.0234; found: 346.0234.

### 3.2. MAO-A and MAO-B Inhibition Assay

The in vitro MAO inhibition test was performed using the available fluorometric method, and the percentages and IC_50_ values of obtained compounds were calculated as previously described by our research group [33,34,35,36,37,38,39].

### 3.3. Enzyme Kinetic Studies

The same materials used in the MAO inhibition assay were used in this experiment. In accordance with the assay given in our previous studies, compounds **2b** and **2h**, which were identified as the most active selective MAO-B inhibitor candidates, were experienced at three independent concentrations of IC_50_/2, IC_50_, and 2(IC_50_) [33,34,35,36,37,38,39]. All processes were evaluated in quadruplicate. The results were analyzed with Microsoft Office Excel 2013 using Lineweaver–Burk diagrams. The V_max_ values of the Lineweaver–Burk plots were replotted versus the inhibitors’ concentrations, and the K_i_ values were determined from the x-axis intercept as -K_i_.

### 3.4. Cytotoxicity Assay

The NIH/3T3 mouse embryonic fibroblast cell line (ATCC^®^ CRL-1658™, London, UK) was used for cytotoxicity assays. The incubation period of NIH/3T3 cells was based on the supplier’s recommendation. NIH/3T3 cells were seeded at 1 × 10^4^ cells into each well of 96-well plates. MTT assay was carried out in accordance with the standards previously described [50,51]. The most effective compounds **2b** and **2h** were tested between 1 mM and 0.000316 mM concentrations. Inhibition % for each concentration was calculated according to the following formula, and IC_50_ values were reported by plotting the % inhibition dose response curve against the compound concentrations tested [50,51,52]:

% inhibition = 100 − (mean sample × 100/mean solvent).

### 3.5. Molecular Docking

A structure based in silico procedure was applied to discover the binding modes of compounds 2b and 2h to *h*MAO-B enzyme active site. The crystal structures of *h*MAO-B (PDB ID: 2V5Z) [40], which was crystallized with safinamide, were retrieved from the Protein Data Bank server (www.pdb.org, accessed at 15 September 2021). The docking procedure was conducted according to the published papers priorly by our research community [33,34,35,36,37,38,39].

## 4. Conclusions

Non-selective MAO inhibitors have many disadvantages. Selective inhibitors are more suitable for use in treatment instead of these inhibitors, which have the risk of side effects such as cheese effect. Parkinson’s disease is one of the most common neurodegenerative diseases after Alzheimer’s disease. In addition to the use of selective MAO-B inhibitors in the treatment of Parkinson’s, they are also used as dual inhibitors in Alzheimer’s disease. The frequency of use in such areas shows how important it is to develop new selective MAO-B inhibitors.

In this study, designed for this purpose, thiosemicarbazone derivatives with proven MAO-B effects were used, and molecular modifications were used to obtain compounds with high activity values. We have reported the synthesis of 12 new benzofuran/benzothiophene derivatives and investigated them for their in vitro MAO inhibitory activity. The derivatives **2b** and **2h** (bearing benzofuran/benzothiophene ring with methoxyethyl moiety) among the synthesized compounds demonstrated remarkable inhibitory activity against MAO-B enzyme. Compounds **2b** and **2h** were found to be the most effective agents in the series with the IC_50_ value of 0.042 ± 0.002 µM and 0.056 ± 0.002 µM, respectively. Furthermore, it was noteworthy that these compounds performed a very similar degree of inhibition to reference drug Selegiline (IC_50_ value = 0.037 ± 0.001 µM). Besides, binding modes of these compounds with the active site of MAO-B enzyme were elucidated owing to molecular docking studies. It was found that these derivatives were settled down in similar and proper positions in the active region. As a result, all this information we gained from this study may enable medicinal chemists to obtain more potent and selective MAO-B inhibitors by doing chemical modifications on benzofuran/benzothiophene derivatives.

## Figures and Tables

**Table:** 

Compounds	X	R
**2a**	–O	–Ethyl
**2b**	–O	–2-Methoxyethyl
**2c**	–O	–Butyl
**2d**	–O	–Cyclohexyl
**2e**	–O	–Phenyl
**2f**	–O	–2-Chlorophenyl
**2g**	–S	–Ethyl
**2h**	–S	–2-Methoxyethyl
**2i**	–S	–Butyl
**2j**	–S	–Cyclohexyl
**2k**	–S	–Phenyl
**2l**	–S	–2-Chlorophenyl

**Table 1:** % Inhibition of compounds **2a**–**2l** against MAO-A and MAO-B.

Compounds	MAO-A % Inhibition		MAO-B % Inhibition	
	10^−3^ M	10^−4^ M	10^−3^ M	10^−4^ M
**2a**	34.128 ± 0.927	22.314 ± 0.824	74.159 ± 1.412	46.356 ± 0.997
**2b**	32.951 ± 0.955	28.357 ± 0.899	**91.625 ± 1.652**	**87.357 ± 1.388**
**2c**	30.357 ± 0.902	26.735 ± 0.820	76.345 ± 1.623	43.451 ± 0.728
**2d**	37.518 ± 0.897	20.856 ± 0.834	82.624 ± 1.318	41.052 ± 0.956
**2e**	36.735 ± 0.910	23.402 ± 0.832	85.486 ± 1.528	40.828 ± 0.827
**2f**	31.057 ± 0.994	24.174 ± 0.711	82.366 ± 1.332	47.398 ± 0.930
**2g**	38.935 ± 0.854	28.692 ± 0.803	78.951 ± 1.702	44.774 ± 0.758
**2h**	36.751 ± 0.741	21.873 ± 0.750	**90.412 ± 1.602**	**84.362 ± 1.325**
**2i**	35.654 ± 0.863	29.162 ± 0.816	84.654 ± 1.628	46.035 ± 0.931
**2j**	33.667 ± 0.729	23.041 ± 0.863	82.923 ± 1.621	42.957 ± 0.965
**2k**	31.065 ± 0.911	27.859 ± 0.815	80.451 ± 1.475	46.651 ± 0.899
**2l**	36.741 ± 0.966	24.389 ± 0.721	83.642 ± 1.597	42.249 ± 0.763
**Clorgiline**	99.411 ± 1.955	98.257 ± 1.824	-	-
**Selegiline**	–	–	99.387 ± 1.385	95.629 ± 1.456

**Table 2:** IC_50_ values of compounds **2b**, **2h**, and Selegiline against MAO-B.

Compounds	MAO-B % Inhibition							IC_50_ (µM)	Selectivity Index
	10^−3^ M	10^−4^ M	10^−5^ M	10^−6^ M	10^−7^ M	10^−8^ M	10^−9^ M		
**2b**	91.625 ± 1.652	87.357 ± 1.388	81.547 ± 1.722	74.951 ± 1.634	66.379 ± 0.975	41.513 ± 0.856	25.820 ± 0.765	0.042 ± 0.002	>23.812
**2h**	90.412 ± 1.602	84.362 ± 1.325	80.792 ± 1.045	72.188 ± 1.388	64.970 ± 1.107	42.826 ± 0.948	30.945 ± 0.782	0.056 ± 0.002	>17.857
**Selegiline**	99.387 ± 1.385	95.629 ± 1.456	86.205 ± 1.200	78.324 ± 1.108	66.871 ± 1.056	42.875 ± 0.865	16.748 ± 0.596	0.037 ± 0.001	–

**Table 3:** IC_50_ values of compounds **2b** and **2h** against the NIH/3T3 cell line.

Compounds	IC_50_ (µM) NIH3T3 Cell Line	IC_50_ (µM) MAO-B Enzyme
**2b**	0.042 ± 0.002	129.631 ± 0.235
**2h**	0.056 ± 0.002	81.266 ± 0.158

## Data Availability

Not applicable.

## Supplementary Materials

The following are available online. Figure S1: ^1^H-NMR spectra of the compound **2a**. Figure S2: ^13^C-NMR spectra of the compound **2a**. Figure S3: APCI-MS spectra of the compound **2a**. Figure S4: HRMS spectra of the compound **2a**. Figure S5: LCMS spectra of the compound **2a**. Figure S6: ^1^H-NMR spectra of the compound **2b**. Figure S7: ^13^CNMR spectra of the compound **2b**. Figure S8: APCI-MS spectra of the compound **2b**. Figure S9: HRMS spectra of the compound **2b**. Figure S10: LCMS spectra of the compound **2b**. Figure S11: ^1^H-NMR spectra of the compound **2c**. Figure S12: ^13^C-NMR spectra of the compound **2c**. Figure S13: APCI-MS spectra of the compound **2c**. Figure S14: HRMS spectra of the compound **2c**. Figure S15: LCMS spectra of the compound **2c**. Figure S16: ^1^H-NMR spectra of the compound **2d**. Figure S17: ^13^C-NMR spectra of the compound **2d**. Figure S18: APCI-MS spectra of the compound **2d**. Figure S19: HRMS spectra of the compound **2d**. Figure S20: LCMS spectra of the compound **2d**. Figure S21: ^1^H-NMR spectra of the compound **2e**. Figure S22: ^13^C-NMR spectra of the compound **2e**. Figure S23: APCI-MS spectra of the compound **2e**. Figure S24: HRMS spectra of the compound **2e**. Figure S25: LCMS spectra of the compound **2e**. Figure S26: ^1^H-NMR spectra of the compound **2f**. Figure S27: ^13^C-NMR spectra of the compound **2f**. Figure S28: APCI-MS spectra of the compound **2f**. Figure S29: HRMS spectra of the compound **2f**. Figure S30: LCMS spectra of the compound **2f**. Figure S31: ^1^H-NMR spectra of the compound **2g**. Figure S32: ^13^C-NMR spectra of the compound **2g**. Figure S33: APCI-MS spectra of the compound **2g**. Figure S34: HRMS spectra of the compound **2g**. Figure S35: LCMS spectra of the compound **2g**. Figure S36: ^1^H-NMR spectra of the compound **2h**. Figure S37: ^13^C-NMR spectra of the compound **2h**. Figure S38: APCI-MS spectra of the compound **2h**. Figure S39: HRMS spectra of the compound **2h**. Figure S40: LCMS spectra of the compound **2h**. Figure S41: ^1^H-NMR spectra of the compound **2i**. Figure S42: ^13^C-NMR spectra of the compound **2i**. Figure S43: APCI-MS spectra of the compound **2i**. Figure S44: HRMS spectra of the compound **2i**. Figure S45: LCMS spectra of the compound **2i**. Figure S46: ^1^H-NMR spectra of the compound **2j**. Figure S47: ^13^C-NMR spectra of the compound **2j**. Figure S48: APCI-MS spectra of the compound **2j**. Figure S49: HRMS spectra of the compound **2j**. Figure S50: LCMS spectra of the compound **2j**. Figure S51: ^1^H-NMR spectra of the compound **2k**. Figure S52: ^13^C-NMR spectra of the compound **2k**. Figure S53: APCI-MS spectra of the compound **2k**. Figure S54: HRMS spectra of the compound **2k**. Figure S55: LCMS spectra of the compound **2k**. Figure S56: ^1^H-NMR spectra of the compound **2l**. Figure S57: ^13^C-NMR spectra of the compound **2l**. Figure S58: APCI-MS spectra of the compound **2l**. Figure S59: HRMS spectra of the compound **2l**. Figure S60: LCMS spectra of the compound **2l**.

## Sample Availability

Samples of the compounds are available from the authors.

## References

[1] Narayan P., Ehsani S., Lindquist S. (2015). Correction: Corrigendum: Combating neurodegenerative disease with chemical probes and model systems. Nat. Chem. Biol..

[2] Trippier P.C., Labby K.J., Hawker D.D., Mataka J.J., Silverman R.B. (2013). Target-and mechanism-based therapeutics for neurodegenerative diseases: Strength in numbers. J. Med. Chem..

[3] Hong R., Li X. (2019). Discovery of monoamine oxidase inhibitors by medicinal chemistry approaches. MedChemComm.

[4] Thase M.E. (2012). The role of monoamine oxidase inhibitors in depression treatment guidelines. J. Clin. Psychiatry.

[5] Youdim M.B., Lavie L. (1994). Selective MAO-A and B inhibitors, radical scavengers and nitric oxide synthase inhibitors in Parkinson’s desease. Life Sci..

[6] Hagenow J., Hagenow S., Grau K., Khanfar M., Hefke L., Proschak E., Stark H. (2020). Reversible small molecule inhibitors of MAO A and MAO B with anilide motifs. Drug Des. Dev. Ther..

[7] Ramsay R.R., Albreht A. (2018). Kinetics, mechanism, and inhibition of monoamine oxidase. J. Neural Transm..

[8] Tong J., Meyer J.H., Furukawa Y., Boileau I., Chang L.-J., Wilson A.A., Houle S. (2013). Distribution of monoamine oxidase proteins in human brain: Implications for brain imaging studies. J. Cereb. Blood Flow Metab..

[9] Finberg J.P., Rabey J.M. (2016). Inhibitors of MAO-A and MAO-B in psychiatry and neurology. Front. Pharmacol..

[10] Youdim M.B., Edmondson D., Tipton K.F. (2006). The therapeutic potential of monoamine oxidase inhibitors. Nat. Rev. Neurosci..

[11] Nagatsu T. (2004). Progress in monoamine oxidase (MAO) research in relation to genetic engineering. Neurotoxicology.

[12] Pacher P., Kecskemeti V. (2004). Trends in the development of new antidepressants. Is there a light at the end of the tunnel?. Curr. Med. Chem..

[13] Hubálek F., Binda C., Khalil A., Li M., Mattevi A., Castagnoli N., Edmondson D.E. (2005). Demonstration of isoleucine 199 as a structural determinant for the selective inhibition of human monoamine oxidase B by specific reversible inhibitors. J. Biol. Chem..

[14] Pålhagen S., Heinonen E., Hägglund J., Kaugesaar T., Mäki-Ikola O., Palm R., the Swedish Parkinson Study Group (2006). Selegiline slows the progression of the symptoms of Parkinson disease. Neurology.

[15] Tripathi R.K., Goshain O., Ayyannan S.R. (2013). Design, synthesis, in vitro MAO-B inhibitory evaluation, and computational studies of some 6-nitrobenzothiazole-derived semicarbazones. ChemMedChem.

[16] Youdim M., Fridkin M., Zheng H. (2004). Novel bifunctional drugs targeting monoamine oxidase inhibition and iron chelation as an approach to neuroprotection in Parkinson’s disease and other neurodegenerative diseases. J. Neural Transm..

[17] Reis J., Manzella N., Cagide F., Mialet-Perez J., Uriarte E., Parini A., Borges F., Binda C. (2018). Tight-binding inhibition of human monoamine oxidase B by chromone analogs: A kinetic, crystallographic, and biological analysis. J. Med. Chem..

[18] Filip V., Kolibas E. (1999). Selegiline in the treatment of Alzheimer’s disease: A long-term randomized placebo-controlled trial. Czech and Slovak Senile Dementia of Alzheimer Type Study Group. J. Psychiatry Neurosci..

[19] Sano M., Ernesto C., Thomas R.G., Klauber M.R., Schafer K., Grundman M., Woodbury P., Growdon J., Cotman C.W., Pfeiffer E. (1997). A controlled trial of selegiline, alpha-tocopherol, or both as treatment for Alzheimer’s disease. N. Engl. J. Med..

[20] Fitton A., Faulds D., Goa K.L. (1992). Moclobemide. Drugs.

[21] Sharma T., Anand R., Hartman R., Rossetti S. (2007). Overlap of cognitive deficits in Parkinson’s (PD) and Alzheimer’s (AD) diseases: Potential use of safinamide: 323. Mov. Disord..

[22] Cambria A., Raudino A., Castelli F., Raciti G., Mazzone P., Buemi G., Pignatello R., Mazzone G. (1996). Structure-activity studies on monoamine oxidase inhibitors by calorimetric and quantum mechanical calculations. J. Enzym. Inhib..

[23] Gritsch S., Guccione S., Hoffmann R., Cambria A., Raciti G., Langer T. (2001). A 3D QSAR study of monoamino oxidase-B inhibitors using the chemical function based pharmacophore generation approach. J. Enzym. Inhib..

[24] La Regina G., Silvestri R., Artico M., Lavecchia A., Novellino E., Befani O., Turini P., Agostinelli E. (2007). New pyrrole inhibitors of monoamine oxidase: Synthesis, biological evaluation, and structural determinants of MAO-A and MAO-B selectivity. J. Med. Chem..

[25] La Regina G., Silvestri R., Gatti V., Lavecchia A., Novellino E., Befani O., Turini P., Agostinelli E. (2008). Synthesis, structure–activity relationships and molecular modeling studies of new indole inhibitors of monoamine oxidases A and B. Bioorganic Med. Chem..

[26] Roth M., Mountjoy C., Amrein R., The International Collaborative Study Group (1996). Moclobemide in elderly patients with cognitive decline and depression: An international double-blind, placebo-controlled trial. Br. J. Psychiatry.

[27] Silvestri R., La Regina G., De Martino G., Artico M., Befani O., Palumbo M., Agostinelli E., Turini P. (2003). Simple, potent, and selective pyrrole inhibitors of monoamine oxidase types A and B. J. Med. Chem..

[28] Tripathi R.K., Sasi V.M., Gupta S.K., Krishnamurthy S., Ayyannan S.R. (2018). Design, synthesis, and pharmacological evaluation of 2-amino-5-nitrothiazole derived semicarbazones as dual inhibitors of monoamine oxidase and cholinesterase: Effect of the size of aryl binding site. J. Enzym. Inhib. Med. Chem..

[29] Tripathi R.K., Rai G.K., Ayyannan S.R. (2016). Exploration of a Library of 3,4-(Methylenedioxy)aniline-Derived Semicarbazones as Dual Inhibitors of Monoamine Oxidase and Acetylcholinesterase: Design, Synthesis, and Evaluation. ChemMedChem.

[30] Mathew G.E., Oh J.M., Mohan K., Kumar K.S., Jayanthi S., Kim H., Mathew B. (2020). Inhibitions of monoamine oxidases and acetylcholinesterase by 1-methyl, 5-phenyl substituted thiosemicarbazones: Synthesis, biochemical, and computational investigations. Process Biochem..

[31] Son S.-Y., Ma J., Kondou Y., Yoshimura M., Yamashita E., Tsukihara T. (2008). Structure of human monoamine oxidase A at 2.2-Å resolution: The control of opening the entry for substrates/inhibitors. Proc. Natl. Acad. Sci. USA.

[32] De Colibus L., Li M., Binda C., Lustig A., Edmondson D.E., Mattevi A. (2005). Three-dimensional structure of human monoamine oxidase A (MAO A): Relation to the structures of rat MAO A and human MAO B. Proc. Natl. Acad. Sci. USA.

[33] Can N.Ö., Osmaniye D., Levent S., Sağlık B.N., Inci B., Ilgın S., Özkay Y., Kaplancıklı Z.A. (2017). Synthesis of new hydrazone derivatives for MAO enzymes inhibitory activity. Molecules.

[34] Can N.Ö., Osmaniye D., Levent S., Sağlık B.N., Korkut B., Atlı Ö., Özkay Y., Kaplancıklı Z.A. (2018). Design, synthesis and biological assessment of new thiazolylhydrazine derivatives as selective and reversible hMAO-A inhibitors. Eur. J. Med. Chem..

[35] Can Ö.D., Osmaniye D., Özkay Ü.D., Sağlık B.N., Levent S., Ilgın S., Baysal M., Özkay Y., Kaplancıklı Z.A. (2017). MAO enzymes inhibitory activity of new benzimidazole derivatives including hydrazone and propargyl side chains. Eur. J. Med. Chem..

[36] Ilgın S., Osmaniye D., Levent S., Sağlık B.N., Acar Çevik U., Çavuşoğlu B.K., Özkay Y., Kaplancıklı Z.A. (2017). Design and synthesis of new benzothiazole compounds as selective hMAO-B inhibitors. Molecules.

[37] Sağlık B.N., Çavuşoğlu B.K., Osmaniye D., Levent S., Çevik U.A., Ilgın S., Özkay Y., Kaplancıklı Z.A., Öztürk Y. (2019). In vitro and in silico evaluation of new thiazole compounds as monoamine oxidase inhibitors. Bioorganic Chem..

[38] Tok F., Sağlık B.N., Özkay Y., Ilgın S., Kaplancıklı Z.A., Koçyiğit-Kaymakçıoğlu B. (2021). Synthesis of new hydrazone derivatives and evaluation of their monoamine oxidase inhibitory activity. Bioorganic Chem..

[39] Tok F., Uğraş Z., Sağlık B.N., Özkay Y., Kaplancıklı Z.A., Koçyiğit-Kaymakçıoğlu B. (2021). Novel 2, 5-disubstituted-1, 3, 4-oxadiazole derivatives as MAO-B inhibitors: Synthesis, biological evaluation and molecular modeling studies. Bioorganic Chem..

[40] Binda C., Wang J., Pisani L., Caccia C., Carotti A., Salvati P., Edmondson D.E., Mattevi A. (2007). Structures of human monoamine oxidase B complexes with selective noncovalent inhibitors: Safinamide and coumarin analogs. J. Med. Chem..

[41] Schrödinger L. (2016). Glide.

[42] Sağlık B.N., Çavuşoğlu B.K., Çevik U.A., Osmaniye D., Levent S., Özkay Y., Kaplancıklı Z.A. (2020). Novel 1,3,4-thiadiazole compounds as potential MAO-A inhibitors–design, synthesis, biological evaluation and molecular modelling. RSC Med. Chem..

[43] Çevik U.A., Osmaniye D., Sağlik B.N., Çavuşoğlu B.K., Levent S., Karaduman A.B., Ilgin S., Karaburun A.Ç., Özkay Y., Kaplancikli Z.A. (2020). Multifunctional quinoxaline-hydrazone derivatives with acetylcholinesterase and monoamine oxidases inhibitory activities as potential agents against Alzheimer’s disease. Med. Chem. Res..

[44] Ichimori K., Stuehr D.J., Atkinson R.N., King S.B. (1999). Synthesis and evaluation of new sulfur-containing L-arginine-derived inhibitors of nitric oxide synthase. J. Med. Chem..

[45] Basheer S.M., Willis A.C., Pace R.J., Sreekanth A. (2016). Spectroscopic and TD-DFT studies on the turn-off fluorescent chemosensor based on anthraldehyde N (4) cyclohexyl thiosemicarbazone for the selective recognition of fluoride and copper ions. Polyhedron.

[46] Huang H., Chen Q., Ku X., Meng L., Lin L., Wang X., Zhu C., Wang Y., Chen Z., Li M. (2010). A series of α-heterocyclic carboxaldehyde thiosemicarbazones inhibit topoisomerase IIα catalytic activity. J. Med. Chem..

[47] Serra S., Moineaux L., Vancraeynest C., Masereel B., Wouters J., Pochet L., Frédérick R. (2014). Thiosemicarbazide, a fragment with promising indolamine-2,3-dioxygenase (IDO) inhibition properties. Eur. J. Med. Chem..

[48] Misra V., Saxena A. (1967). Potential Antiviral and Antituberculous Compounds. III. N-1-(4-methoxy naphthylidene/2-benzofurylidene)-N-4-(aryl) thiosemicarbazides; 5-carboxymethyl, 3-aryl, thiazolidine-2, 4-dione hydrazones; 3-aryl, thiazolidine-2, 4-dione hydrazones and N-1-(4-methoxy naphthylidene/2-benzofurylidene)-N-4-(aryl) amino guanidines. J. für Praktische Chemie.

[49] Qiao Y., Che Y., Yu Y., Tang Y., Liu L., Zhao X., Zhao J. (2018). Solid-state photochromic properties, mechanism and electrospun membranes of (E)-2-(benzo [b] thiophen-2-ylmethylene)-N-ethylhydrazine-1-carbothioamide. Dyes Pigment..

[50] Sağlık B.N., Ilgın S., Özkay Y. (2016). Synthesis of new donepezil analogues and investigation of their effects on cholinesterase enzymes. Eur. J. Med. Chem..

[51] Özkay Ü.D., Can Ö.D., Sağlık B.N., Çevik U.A., Levent S., Özkay Y., Ilgın S., Atlı Ö. (2016). Design, synthesis, and AChE inhibitory activity of new benzothiazole–piperazines. Bioorganic Med. Chem. Lett..

[52] Patel S., Gheewala N., Suthar A., Shah A. (2009). In-vitro cytotoxicity activity of Solanum nigrum extract against Hela cell line and Vero cell line. Int. J. Pharm. Pharm. Sci..

