# Supplementary material for: Novel Thiosemicarbazone Derivatives: In Vitro and In Silico Evaluation as Potential MAO-B Inhibitors
Source: Molecules. 2021 Nov 2;26(21):6640. doi: 10.3390/molecules26216640 (PMC8587871; doi:10.3390/molecules26216640)
Supplement: Supplementary file 1 [file molecules-26-06640-s001.zip › molecules-1403224-supplementary.pdf]

Novel thiosemicarbazone Derivatives: *In vitro* and *In Silico* Evaluation as Potential MAO-B Inhibitors

Derya Osmaniye[a,b,*], Berkant Kurban[a], Begüm Nurpelin Sağlık[a,b], Serkan Levent[a,b], Yusuf Özkay[a,b], Zafer Asım Kaplancıklı[a]

[a]*Department of Pharmaceutical Chemistry, Faculty of Pharmacy, Anadolu University, 26470 Eskişehir, Turkey*

[b]*Doping and Narcotic Compounds Analysis Laboratory, Faculty of Pharmacy, Anadolu University, 26470 Eskişehir, Turkey*

* Corresponding author.

E-mail address: dosmaniye@anadolu.edu.tr (D. Osmaniye).

Tel: +90-222-3350580/3778 *Fax*: +90-222-3350750.

Address: Anadolu University, Faculty of Pharmacy, Department of Pharmaceutical Chemistry, 26470, Eskişehir, Turkey.

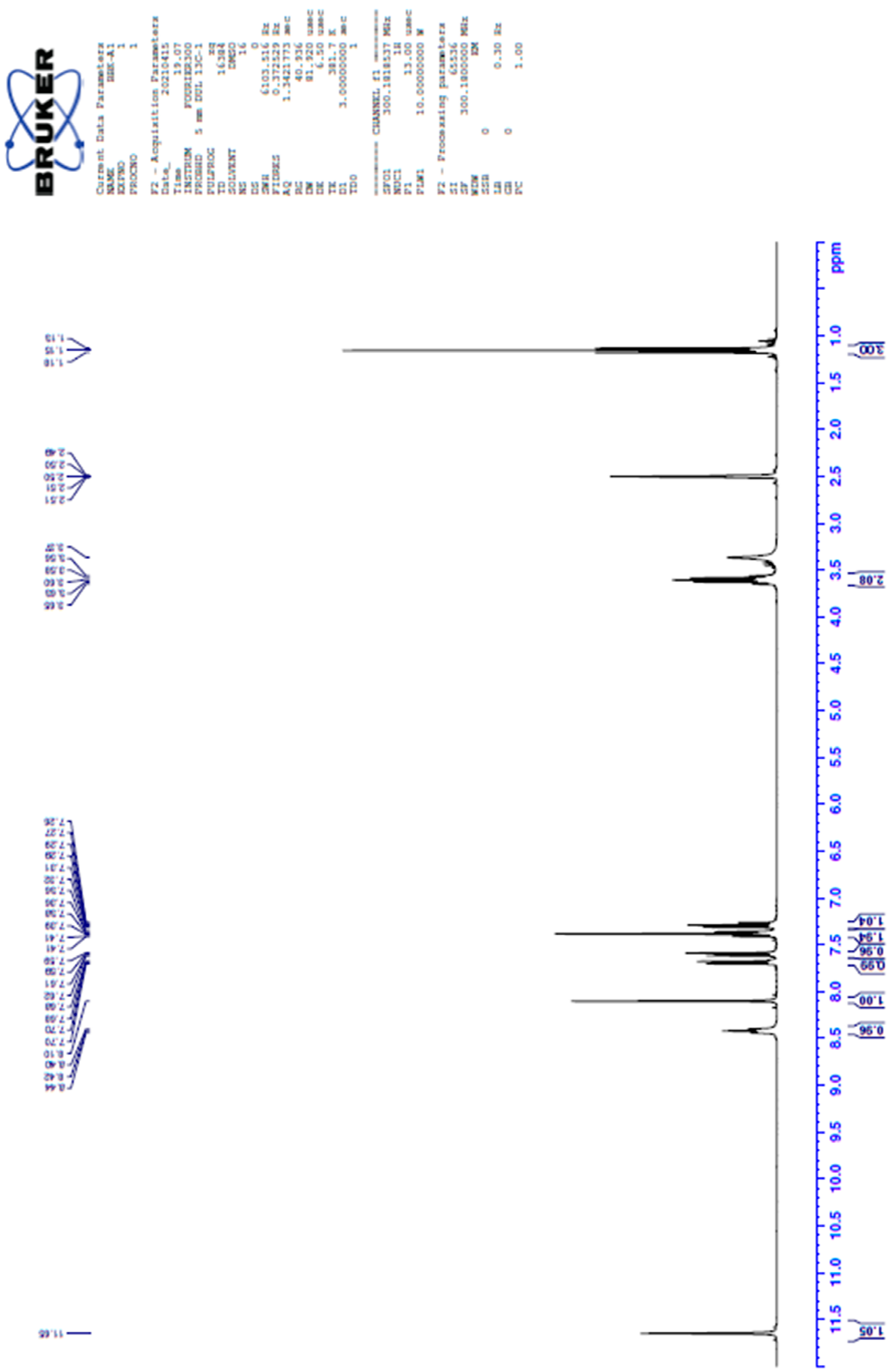

Figure S1: ^{1}H-NMR spectra of the compound **2a**

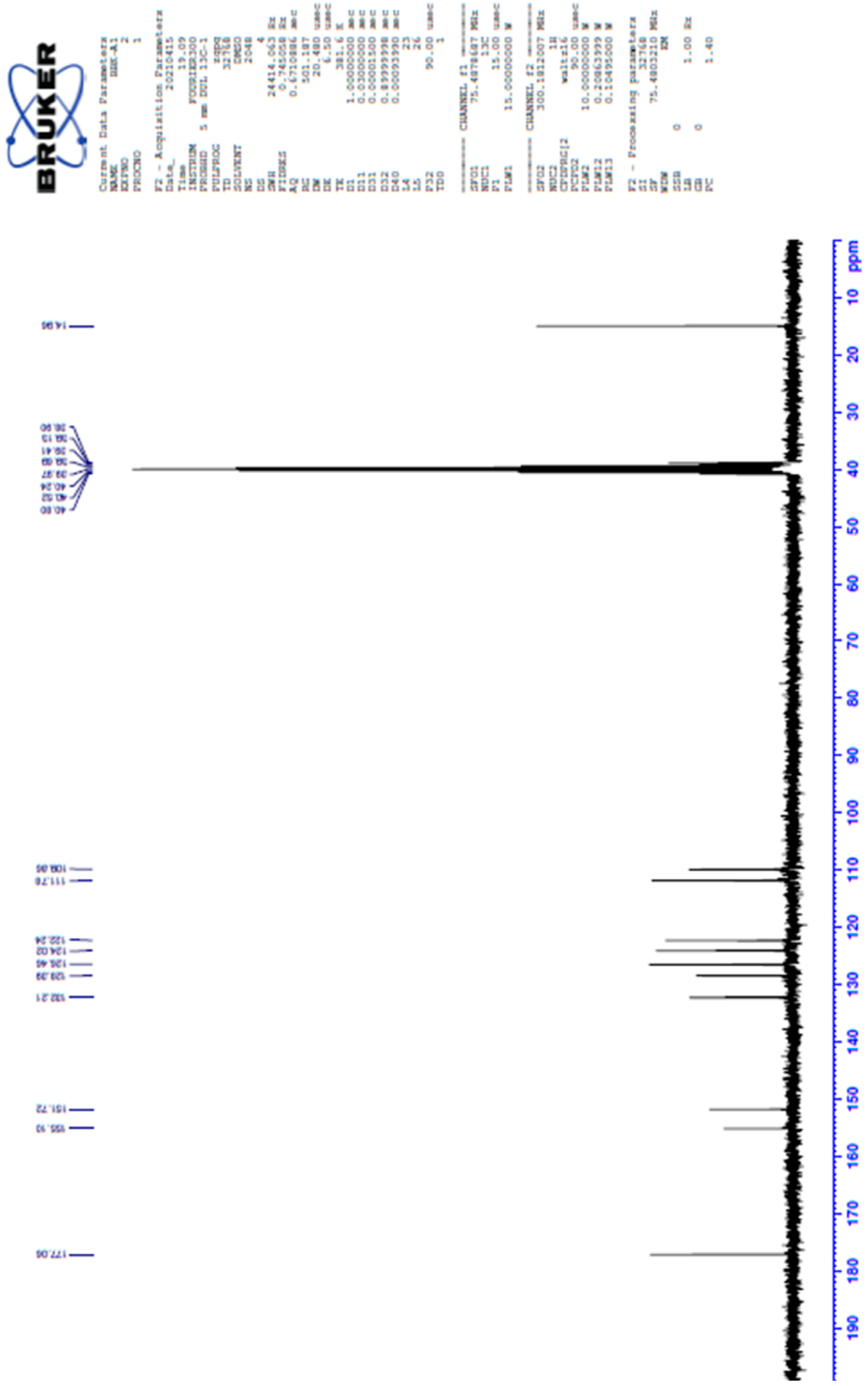

Figure S2: ^{13}C-NMR spectra of the compound **2a**

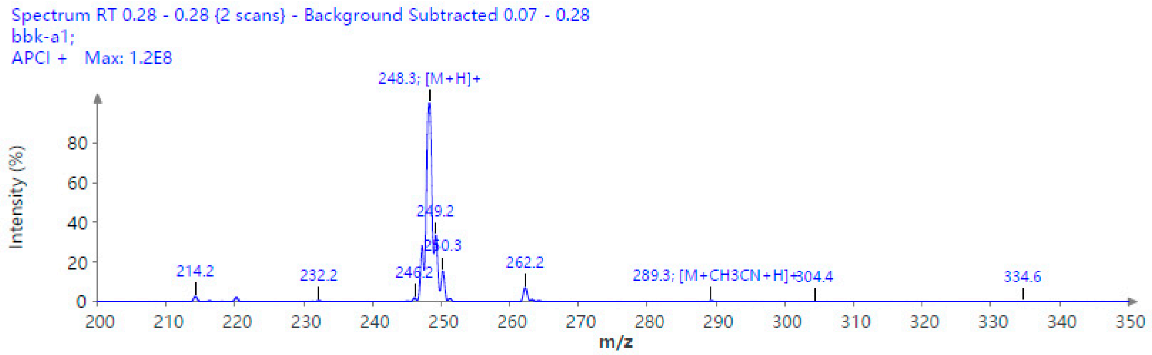

Figure S3: APCI-MS spectra of the compound **2a**

Formula Predictor Report - BBK-A1_51.lcd

Data File: C:\LabSolutions\Data\Analiz\derya\BBK-A1_51.lcd

Elmt	Val.	Min	Max	Elmt	Val.	Min	Max	Elmt	Val.	Min	Max	Elmt	Val.	Min	Max	Use Adduct
H	1	0	40	O	2	0	6	S	2	0	4	Ru	2	0	0	H
C	4	0	40	F	1	0	0	Cl	1	0	0	Pd	2	0	0	
N	3	2	5	P	3	0	0	Br	1	0	0	I	3	0	0	

Error Margin (ppm): 5
HC Ratio: unlimited
Max Isotopes: 3
MSn Iso RI (%): 10.00

DBE Range: 0.0 - 20.0
Apply N Rule: yes
Isotope RI (%): 1.00
MSn Logic Mode: AND

Electron Ions: both
Use MSn Info: yes
Isotope Res: 9000
Max Results: 150

Event#: 1 MS(E+) Ret. Time : 3.160 Scan# : 475

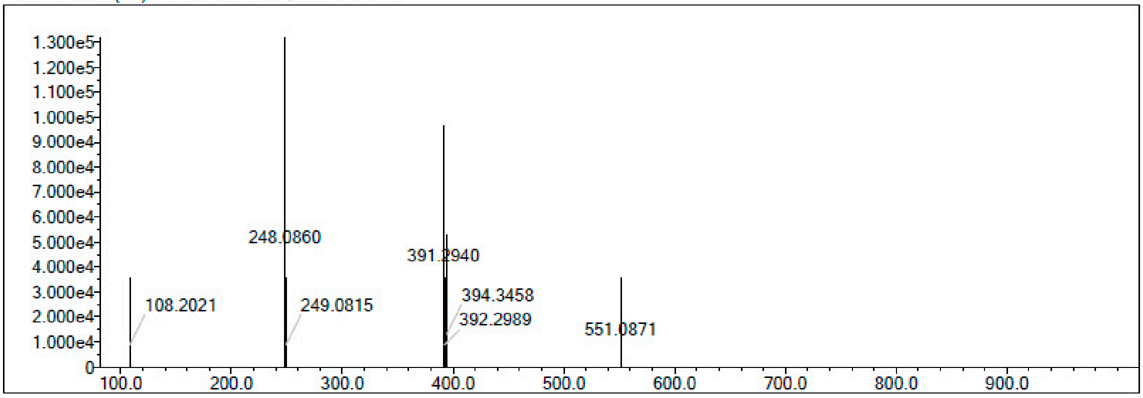

Measured region for 248.0860 m/z

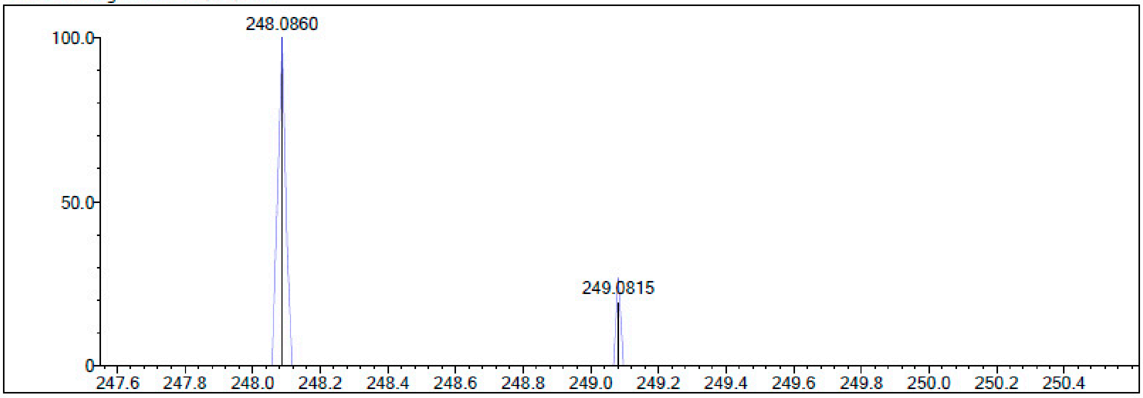

C12 H13 N3 O S [M+H]+ : Predicted region for 248.0852 m/z

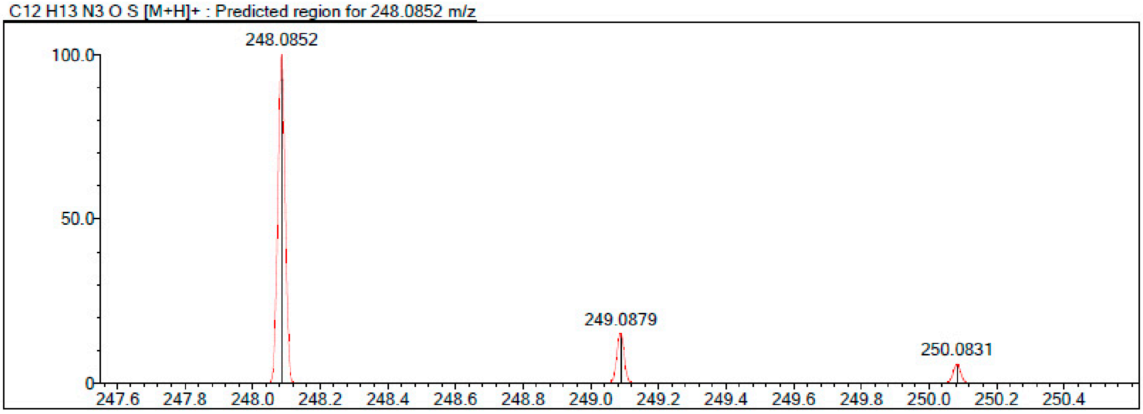

Rank	Score	Formula (M)	Ion	Meas. m/z	Pred. m/z	Df. (mDa)	Df. (ppm)	Iso	DBE
1	45.76	C12 H13 N3 O S	[M+H]+	248.0860	248.0852	0.8	3.22	48.45	8.0

Figure S4: HRMS spectra of the compound **2a**

==== Shimadzu LCMSsolution Analysis Report ====

Acquired by	: Admin
Sample Name	: BBK-A1
Sample ID	:
Vail #	: 61
Injection Volume	: 0.3 uL
Data File Name	: BBK-A1_51.lcd
Method File Name	: isocratic_serkan.lcm
Batch File Name	: batch.lcb
Report File Name	: DefaultLCMS.lcr
Data Acquired	: 10.08.2021 14:59:31
Data Processed	: 12.08.2021 14:18:30

<Chromatogram>

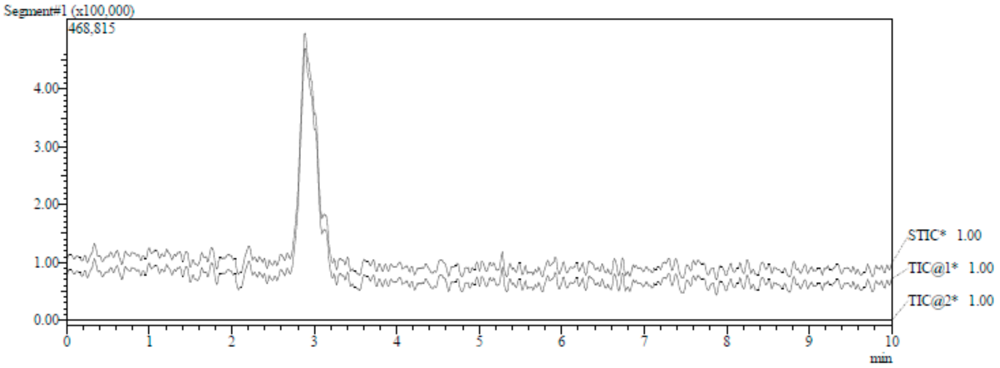

Chromatogram
BBK-A1 C:\LabSolutions\Data\Analiz\derya\BBK-A1_51.lcd

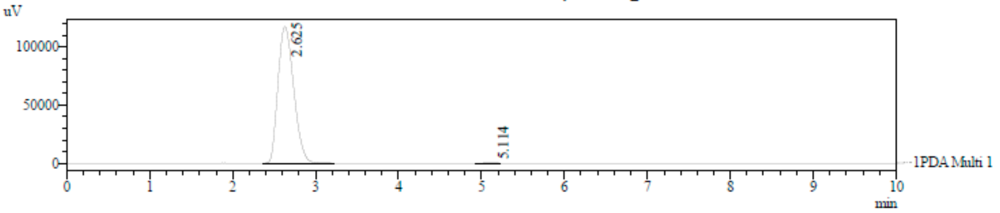

1 PDA Multi 1 / 341nm 4nm

PeakTable

PDA Ch1 341nm 4nm

Peak#	Ret. Time	Area	Height	Area %	Height %
1	2.625	1545614	117241	99.902	99.885
2	5.114	1512	135	0.098	0.115
Total		1547126	117376	100.000	100.000

Figure S5: LCMS spectra of the compound **2a**

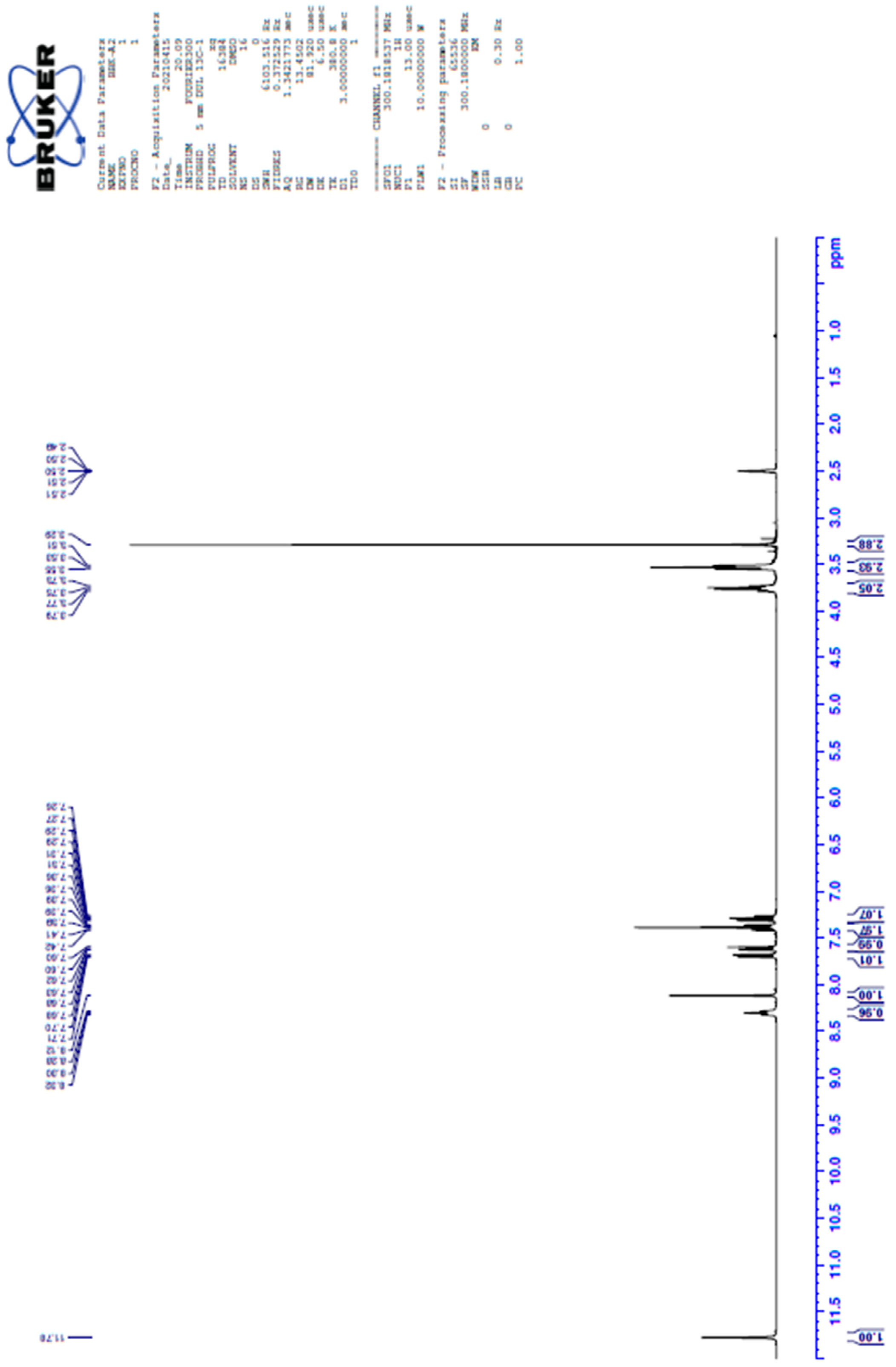

Figure S6: ^{1}H-NMR spectra of the compound **2b**

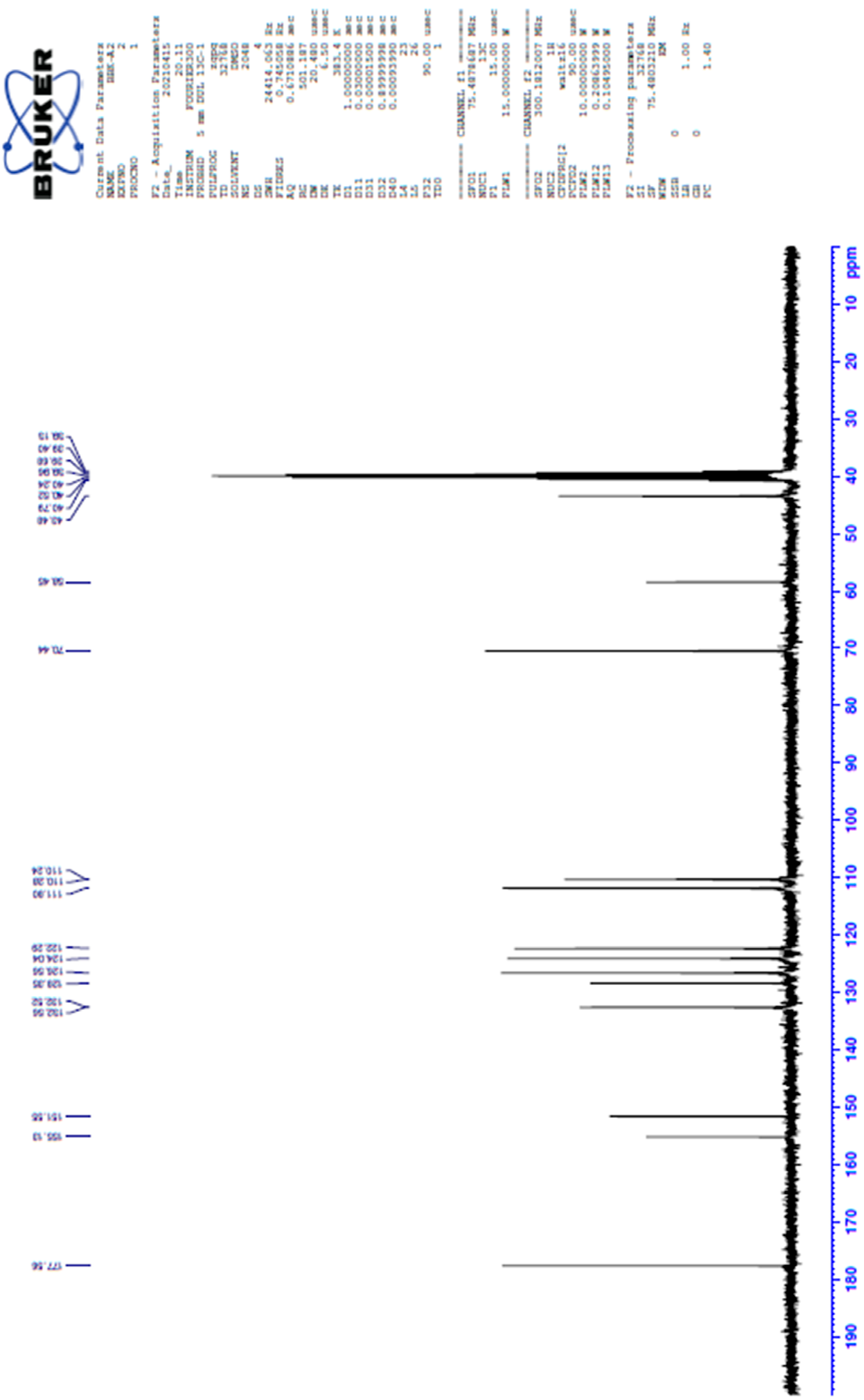

Figure S7: ^{13}C-NMR spectra of the compound **2b**

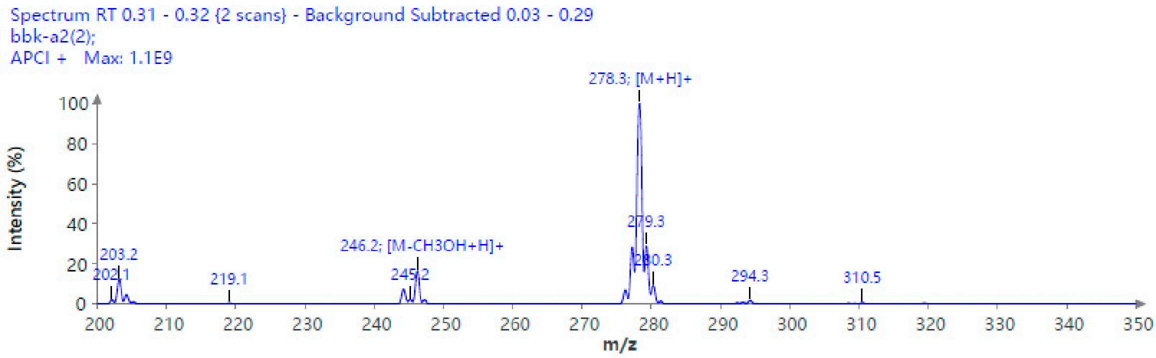

Figure S8: APCI-MS spectra of the compound **2b**

Formula Predictor Report - BBK-A2_56.lcd   Page 1 of 1

Data File: C:\LabSolutions\Data\Analiz\derya\BBK-A2_56.lcd

Elmt	Val.	Min	Max	Elmt	Val.	Min	Max	Elmt	Val.	Min	Max	Elmt	Val.	Min	Max	Use Adduct
H	1	0	40	O	2	0	5	S	2	1	1	Ru	2	0	0	H
C	4	0	40	F	1	0	0	Cl	1	0	0	Pd	2	0	0	
N	3	2	5	P	3	0	0	Br	1	0	0	I	3	0	0	

Error Margin (ppm):	10	DBE Range:	0.0 - 20.0	Electron Ions:	both
HC Ratio:	unlimited	Apply N Rule:	yes	Use MSn Info:	yes
Max Isotopes:	3	Isotope RI (%):	1.00	Isotope Res:	9000
MSn Iso RI (%):	10.00	MSn Logic Mode:	AND	Max Results:	150

Event#: 1 MS(E+)   Ret. Time : 2.787   Scan# : 419

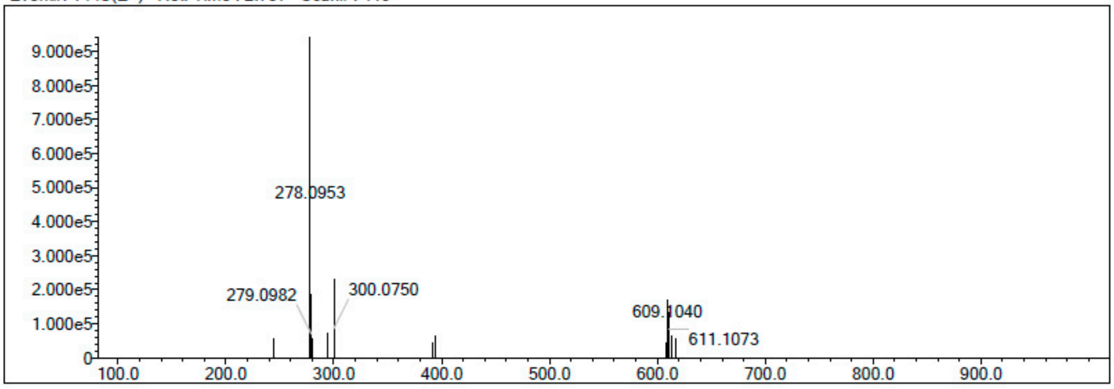

Measured region for 278.0953 m/z

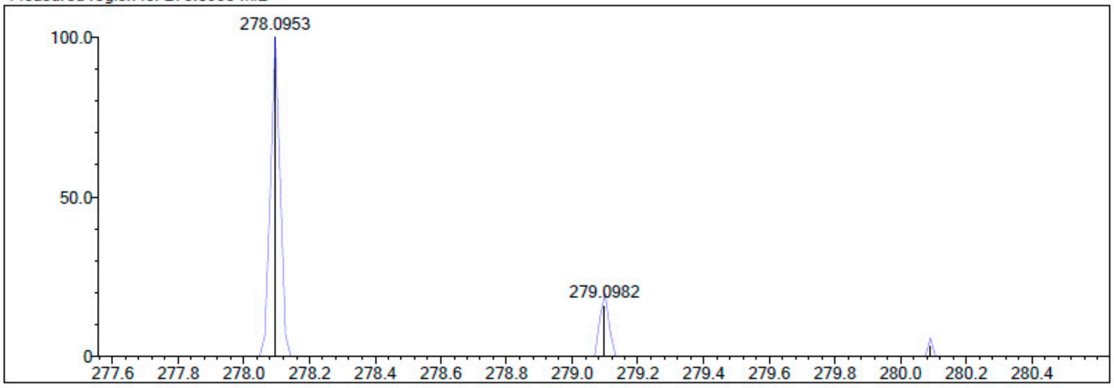

C13 H15 N3 O2 S [M+H]+ : Predicted region for 278.0958 m/z

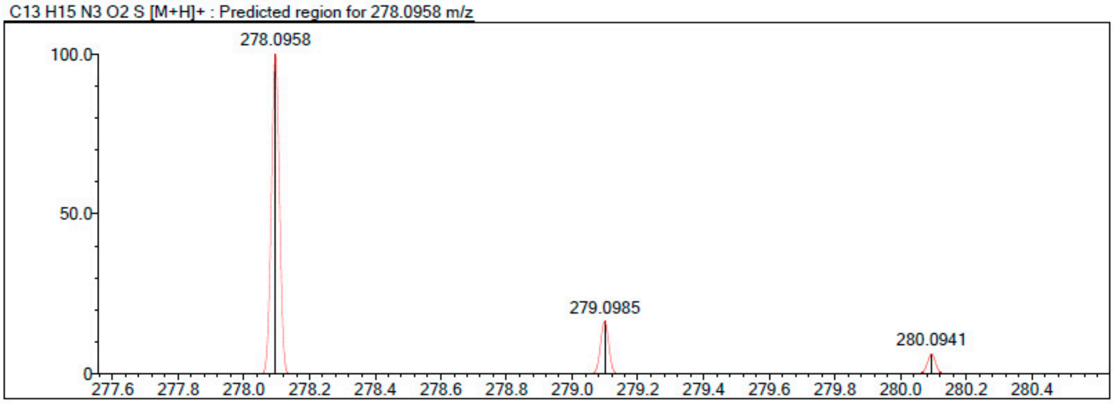

Rank	Score	Formula (M)	Ion	Meas. m/z	Pred. m/z	Df. (mDa)	Df. (ppm)	Iso	DBE
1	86.18	C13 H15 N3 O2 S	[M+H]+	278.0953	278.0958	-0.5	-1.80	87.94	8.0

Figure S9: HRMS spectra of the compound **2b**

==== Shimadzu LCMSsolution Analysis Report ====

Acquired by	: Admin
Sample Name	: BBK-A2
Sample ID	:
Vail #	: 22
Injection Volume	: 0.3 uL
Data File Name	: BBK-A2_56.lcd
Method File Name	: isocratic_serkan.lcm
Batch File Name	: batch.lcb
Report File Name	: DefaultLCMS.lcr
Data Acquired	: 13.08.2021 10:39:40
Data Processed	: 13.08.2021 11:39:51

<Chromatogram>

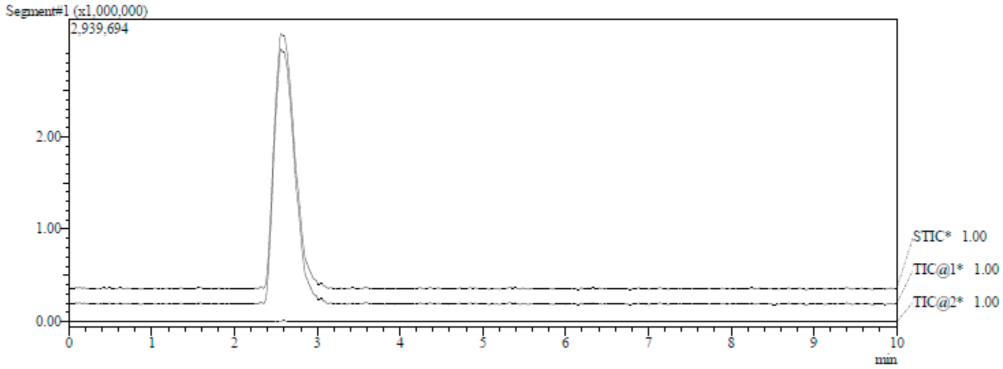

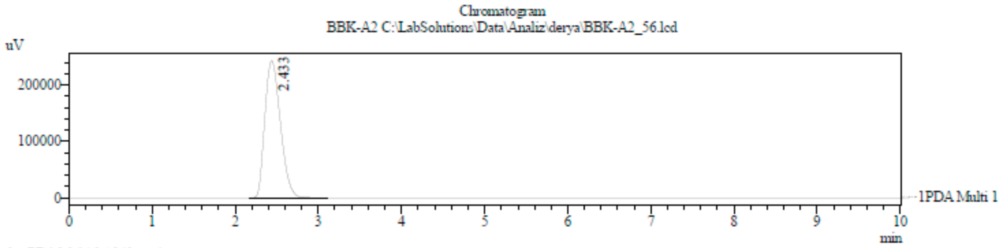

1 PDA Multi 1 / 341nm 4nm

PeakTable

PDA Ch1 341nm 4nm

Peak#	Ret. Time	Area	Height	Area %	Height %
1	2.433	3164120	243320	100.000	100.000
Total		3164120	243320	100.000	100.000

Figure S10: LCMS spectra of the compound **2b**

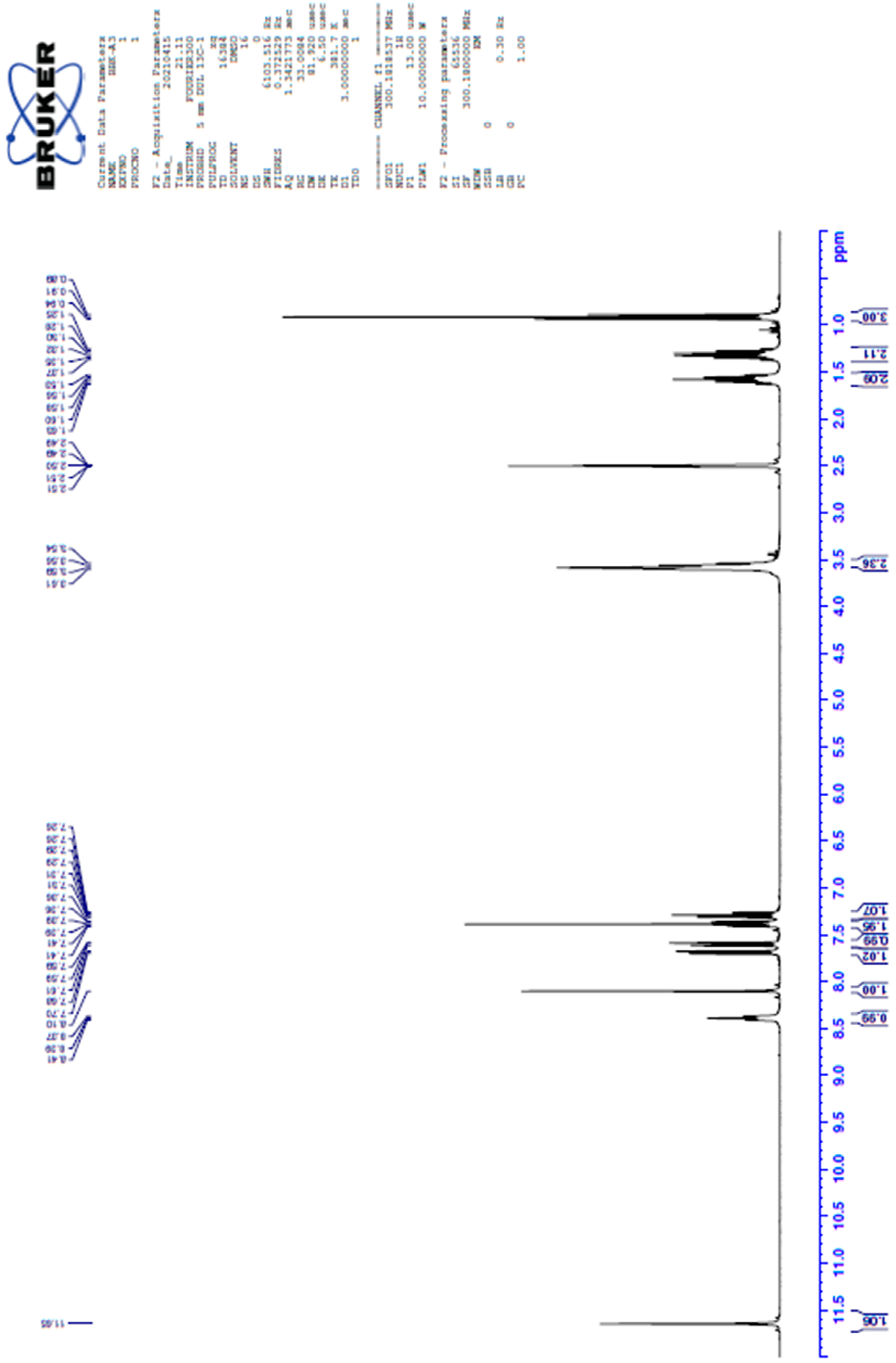

Figure S11: ^{1}H-NMR spectra of the compound **2c**

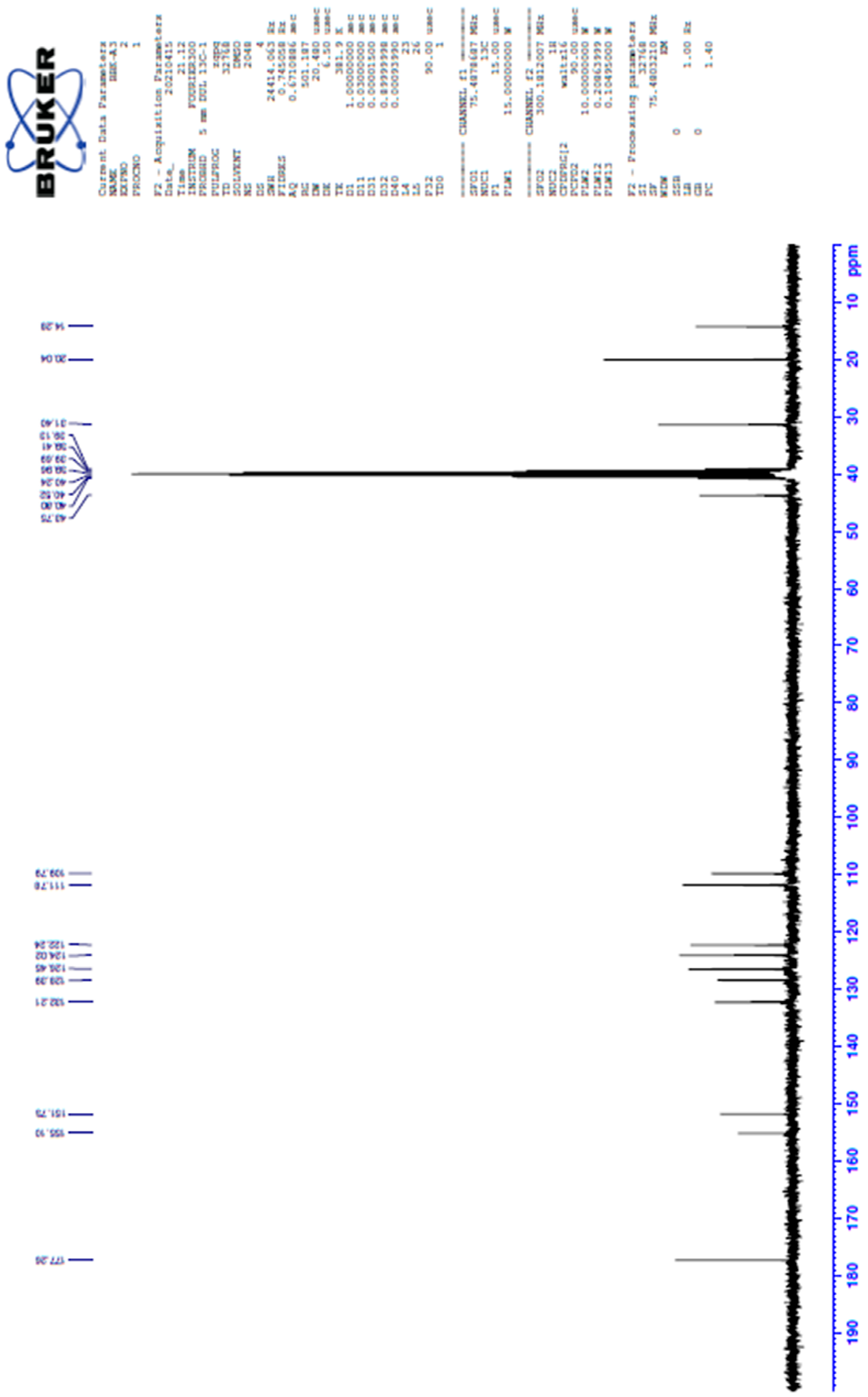

Figure S12: ^{13}C-NMR spectra of the compound **2c**

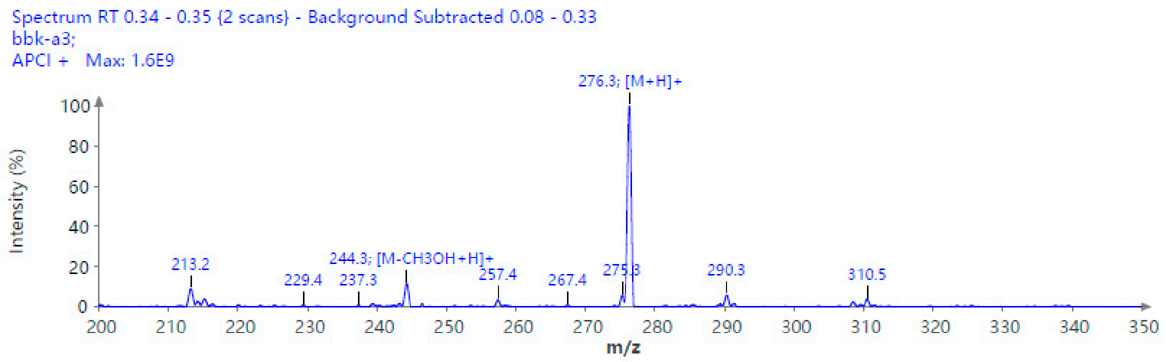

Figure S13: APCI-MS spectra of the compound **2c**

Data File: C:\LabSolutions\Data\Analiz\derya\BBK-A3_57.lcd

Elmt	Val.	Min	Max	Elmt	Val.	Min	Max	Elmt	Val.	Min	Max	Elmt	Val.	Min	Max	Use Adduct
H	1	0	40	O	2	0	5	S	2	1	1	Ru	2	0	0	H
C	4	0	40	F	1	0	0	Cl	1	0	0	Pd	2	0	0	
N	3	2	5	P	3	0	0	Br	1	0	0	I	3	0	0	

Error Margin (ppm):	10	DBE Range:	0.0 - 20.0	Electron Ions:	both
HC Ratio:	unlimited	Apply N Rule:	yes	Use MSn Info:	yes
Max Isotopes:	3	Isotope RI (%):	1.00	Isotope Res:	9000
MSn Iso RI (%):	10.00	MSn Logic Mode:	AND	Max Results:	150

Event#: 1 MS(E+) Ret. Time : 4.093 Scan# : 615

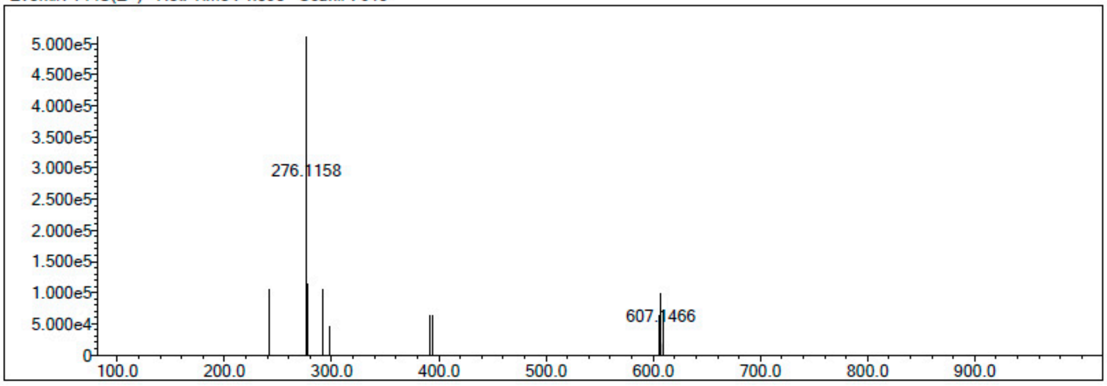

Measured region for 276.1158 m/z

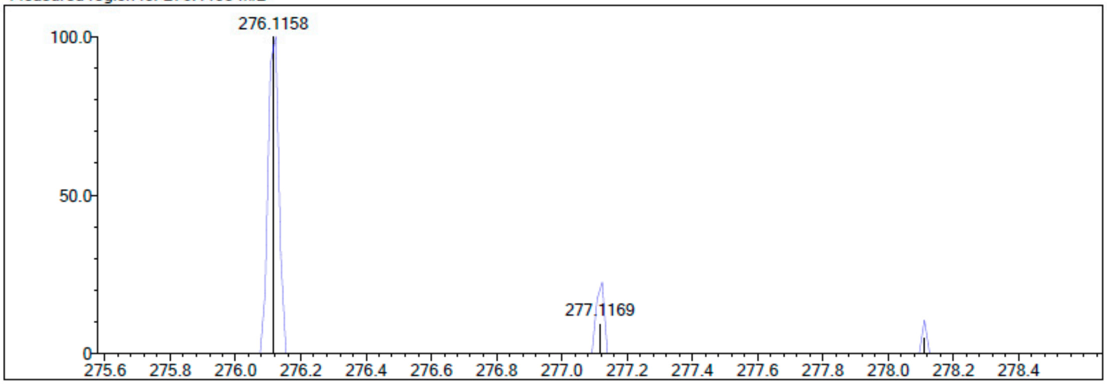

C14 H17 N3 O S [M+H]+ : Predicted region for 276.1165 m/z

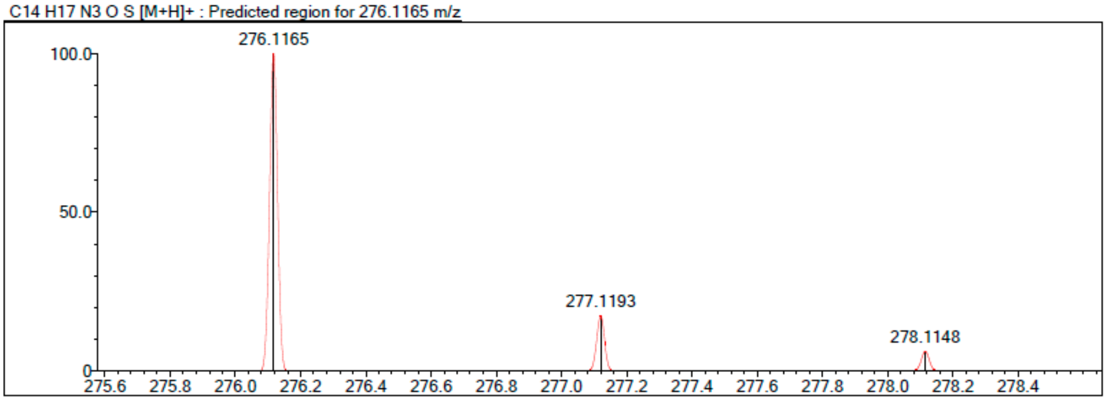

Rank	Score	Formula (M)	Ion	Meas. m/z	Pred. m/z	Df. (mDa)	Df. (ppm)	Iso	DBE
1	73.36	C14 H17 N3 O S	[M+H]+	276.1158	276.1165	-0.7	-2.54	76.30	8.0

Figure S14: HRMS spectra of the compound **2c**

==== Shimadzu LCMSsolution Analysis Report ====

Acquired by	: Admin
Sample Name	: BBK-A3
Sample ID	:
Vail #	: 23
Injection Volume	: 0.3 uL
Data File Name	: BBK-A3_57.lcd
Method File Name	: isocratic_serkan.lcm
Batch File Name	: batch.lcb
Report File Name	: DefaultLCMS.lcr
Data Acquired	: 13.08.2021 10:50:13
Data Processed	: 13.08.2021 11:44:14

<Chromatogram>

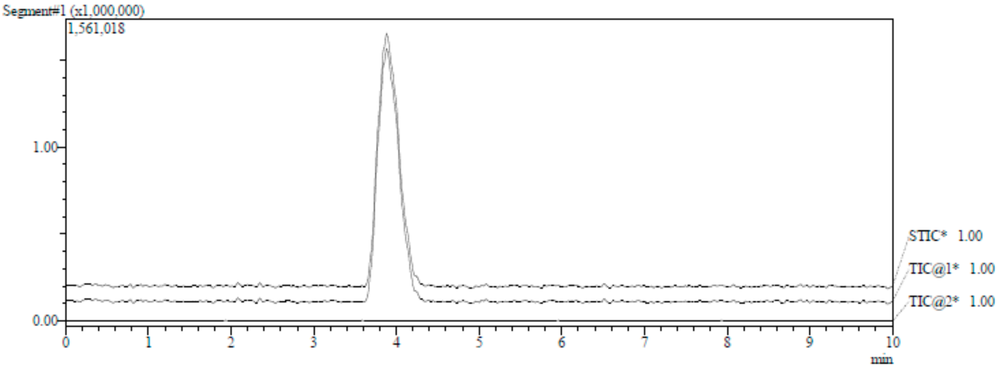

Chromatogram
BBK-A3 C:\LabSolutions\Data\Analiz\derya\BBK-A3_57.lcd

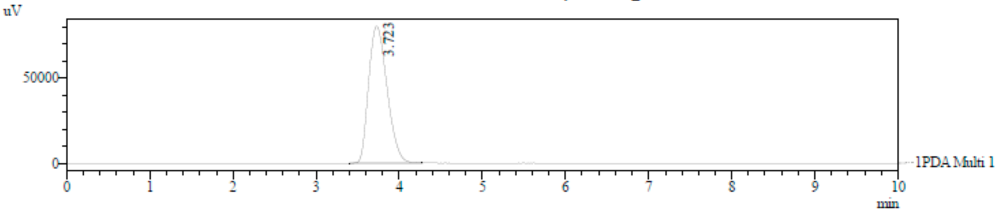

1 PDA Multi 1 / 341nm 4nm

PeakTable

PDA Ch1 341nm 4nm

Peak#	Ret. Time	Area	Height	Area %	Height %
1	3.723	1241295	80294	100.000	100.000
Total		1241295	80294	100.000	100.000

Figure S15: LCMS spectra of the compound **2c**

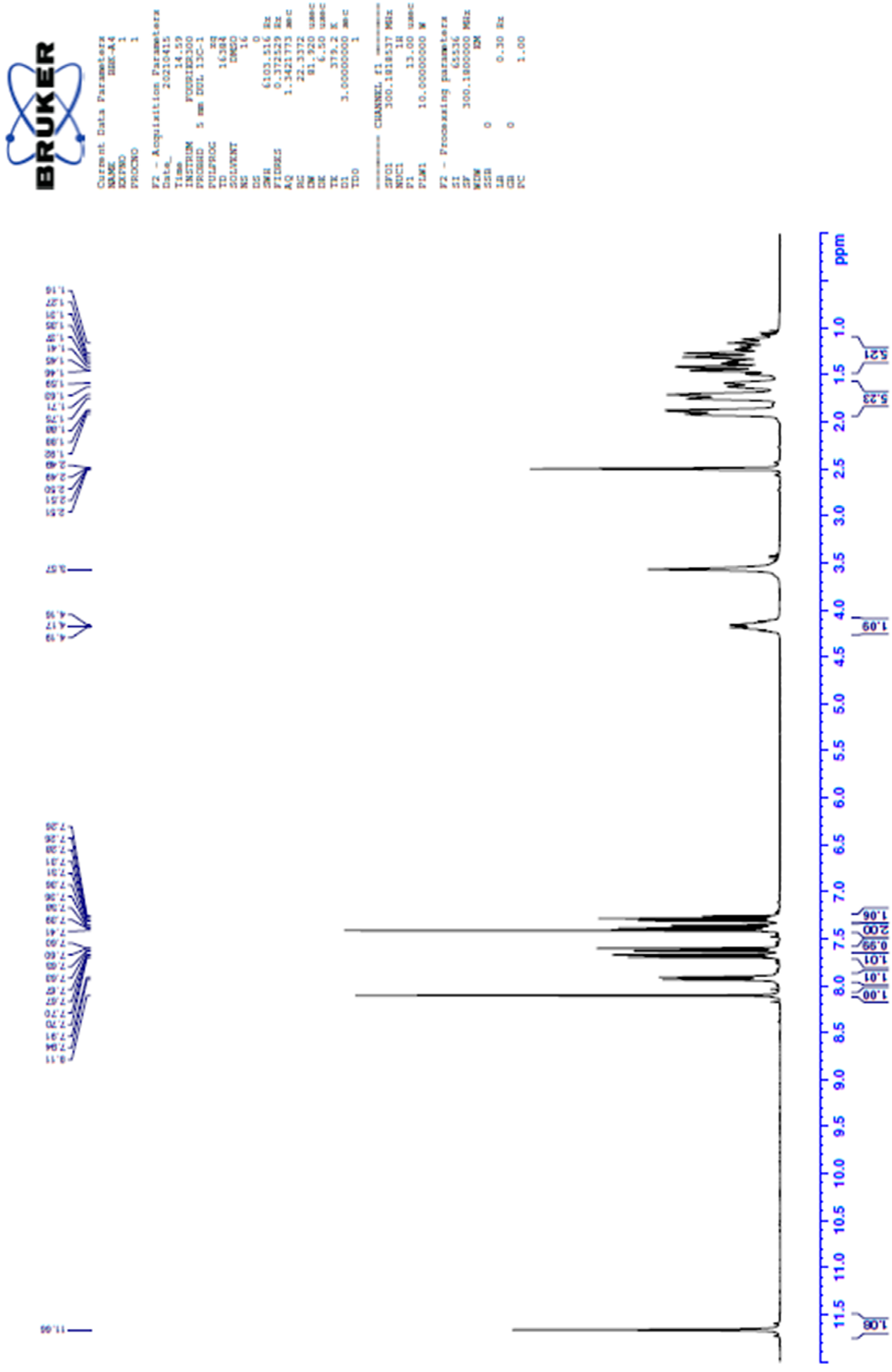

Figure S16: ^{1}H-NMR spectra of the compound **2d**

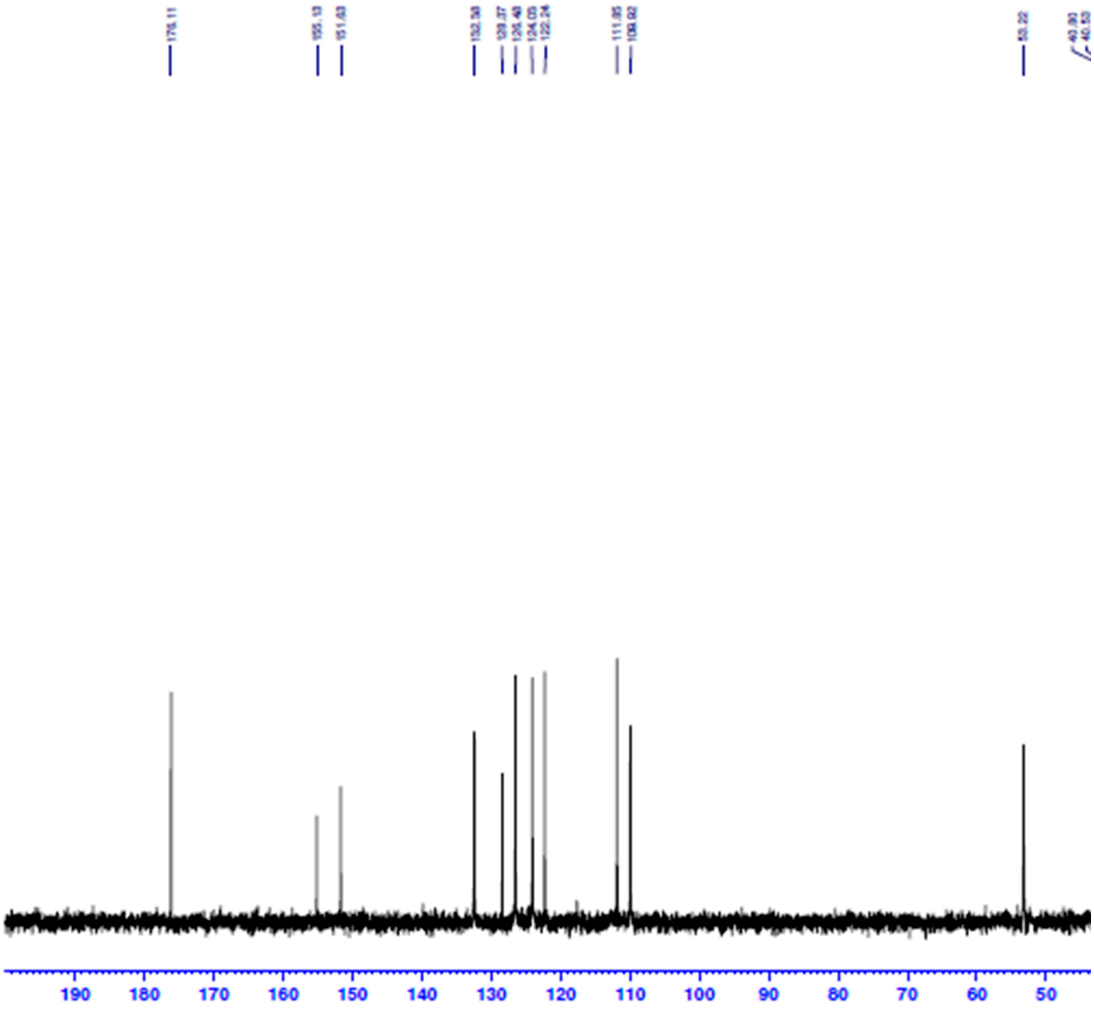

Figure S17: ^{13}C-NMR spectra of the compound **2d**

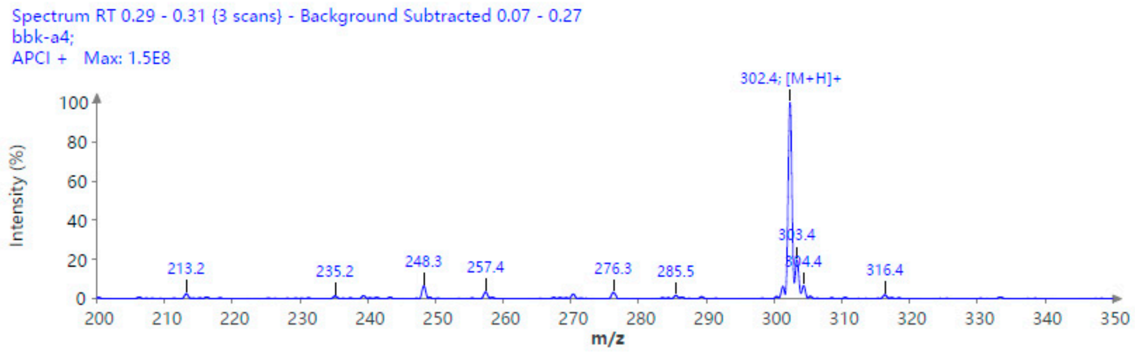

Figure S18: APCI-MS spectra of the compound **2d**

Data File: C:\LabSolutions\Data\Analiz\derya\BBK-A4_1.lcd

Elmt	Val.	Min	Max	Elmt	Val.	Min	Max	Elmt	Val.	Min	Max	Elmt	Val.	Min	Max	Use Adduct
H	1	0	40	O	2	0	4	S	2	1	2	Ru	2	0	0	H
C	4	0	40	F	1	0	0	Cl	1	0	1	Pd	2	0	0	
N	3	2	5	P	3	0	0	Br	1	0	0	I	3	0	0	

Error Margin (ppm):	10	DBE Range:	6.0 - 13.0	Electron Ions:	both
HC Ratio:	unlimited	Apply N Rule:	yes	Use MSn Info:	yes
Max Isotopes:	3	Isotope RI (%):	1.00	Isotope Res:	9000
MSn Iso RI (%):	10.00	MSn Logic Mode:	AND	Max Results:	150

Event#: 1 MS(E+) Ret. Time : 3.600 Scan# : 541

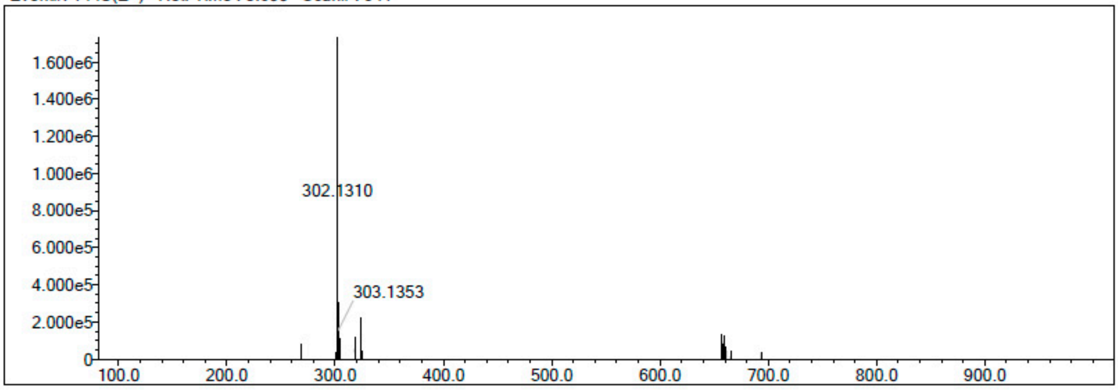

Measured region for 302.1310 m/z

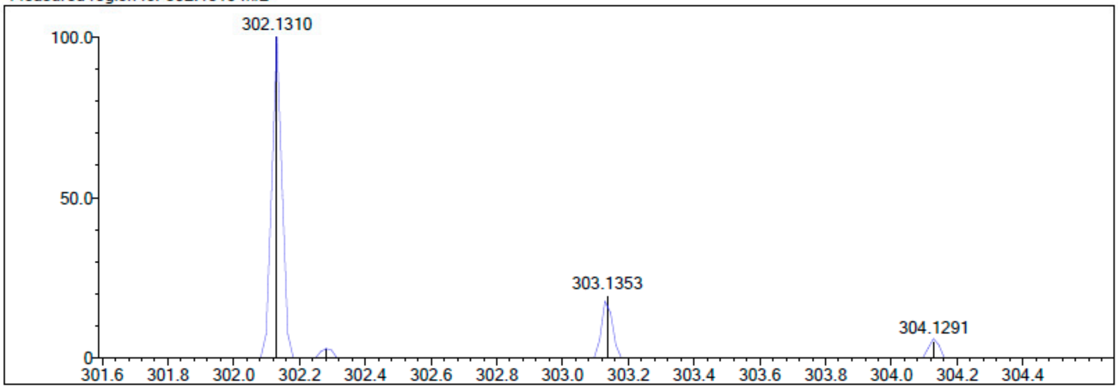

C16 H19 N3 O S [M+H]+ : Predicted region for 302.1322 m/z

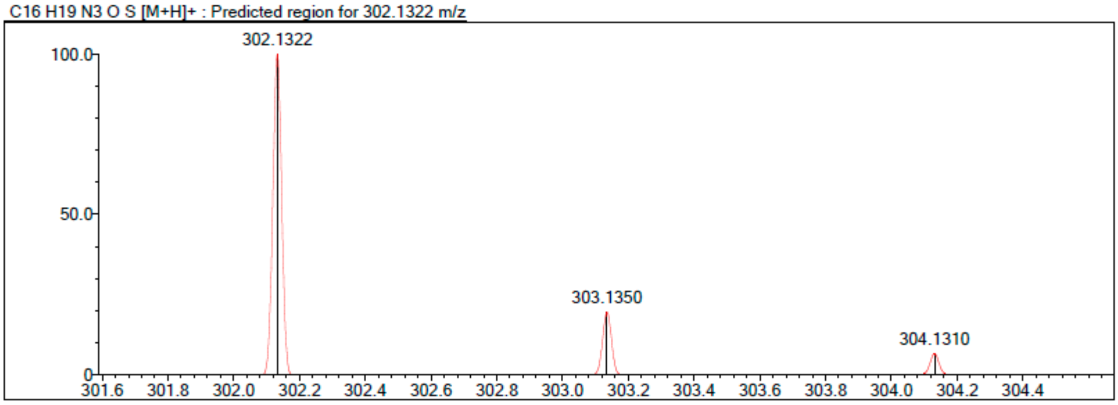

Rank	Score	Formula (M)	Ion	Meas. m/z	Pred. m/z	Df. (mDa)	Df. (ppm)	Iso	DBE
1	89.67	C16 H19 N3 O S	[M+H]+	302.1310	302.1322	-1.2	-3.97	96.86	9.0

Figure S19: HRMS spectra of the compound **2d**

==== Shimadzu LCMSsolution Analysis Report ====

Acquired by	: Admin
Sample Name	: BBK-A4
Sample ID	:
Vail #	: 64
Injection Volume	: 0.3 uL
Data File Name	: BBK-A4_54.lcd
Method File Name	: isocratic_serkan.lcm
Batch File Name	: batch.lcb
Report File Name	: DefaultLCMS.lcr
Data Acquired	: 10.08.2021 15:31:09
Data Processed	: 12.08.2021 14:15:51

<Chromatogram>

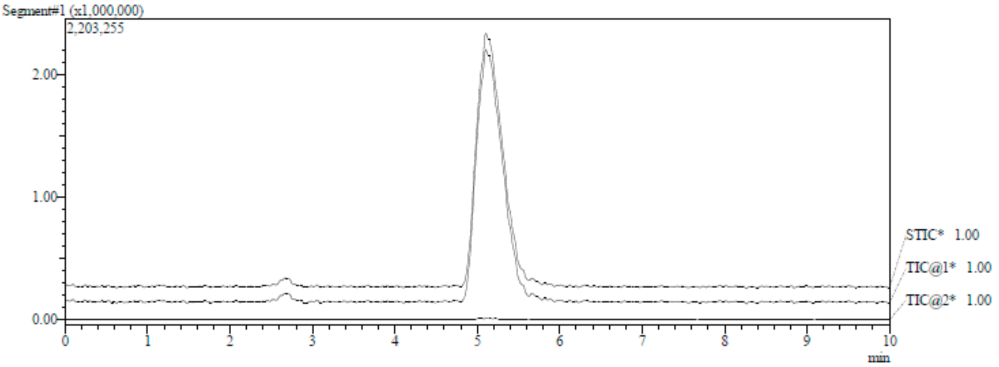

Chromatogram
BBK-A4 C:\LabSolutions\Data\Analiz\derya\BBK-A4_54.lcd

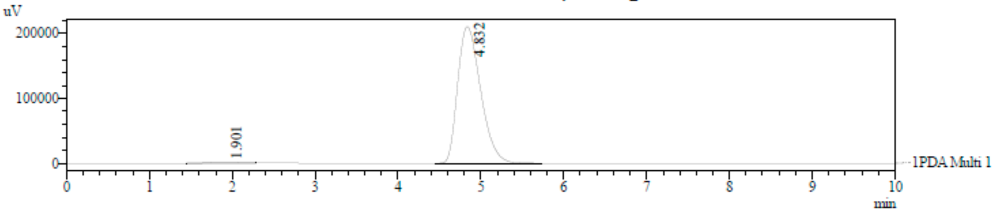

1 PDA Multi 1 / 341nm 4nm

PeakTable

PDA Ch1 341nm 4nm

Peak#	Ret. Time	Area	Height	Area %	Height %
1	1.901	17205	862	0.423	0.407
2	4.832	4050923	210731	99.577	99.593
Total		4068128	211592	100.000	100.000

Figure S20: APCI-MS spectra of the compound **2d**

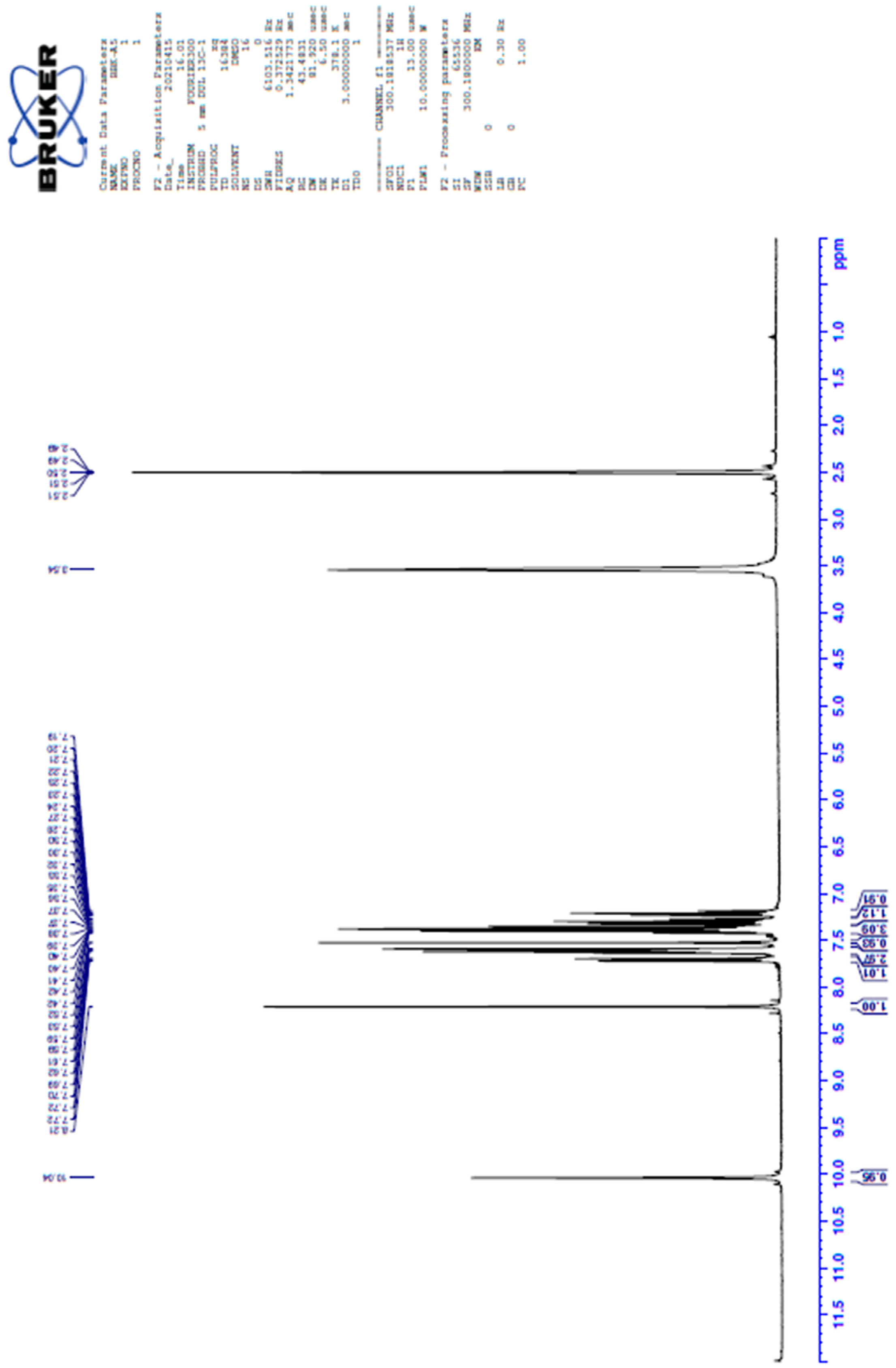

Figure S21: ^{1}H-NMR spectra of the compound **2e**

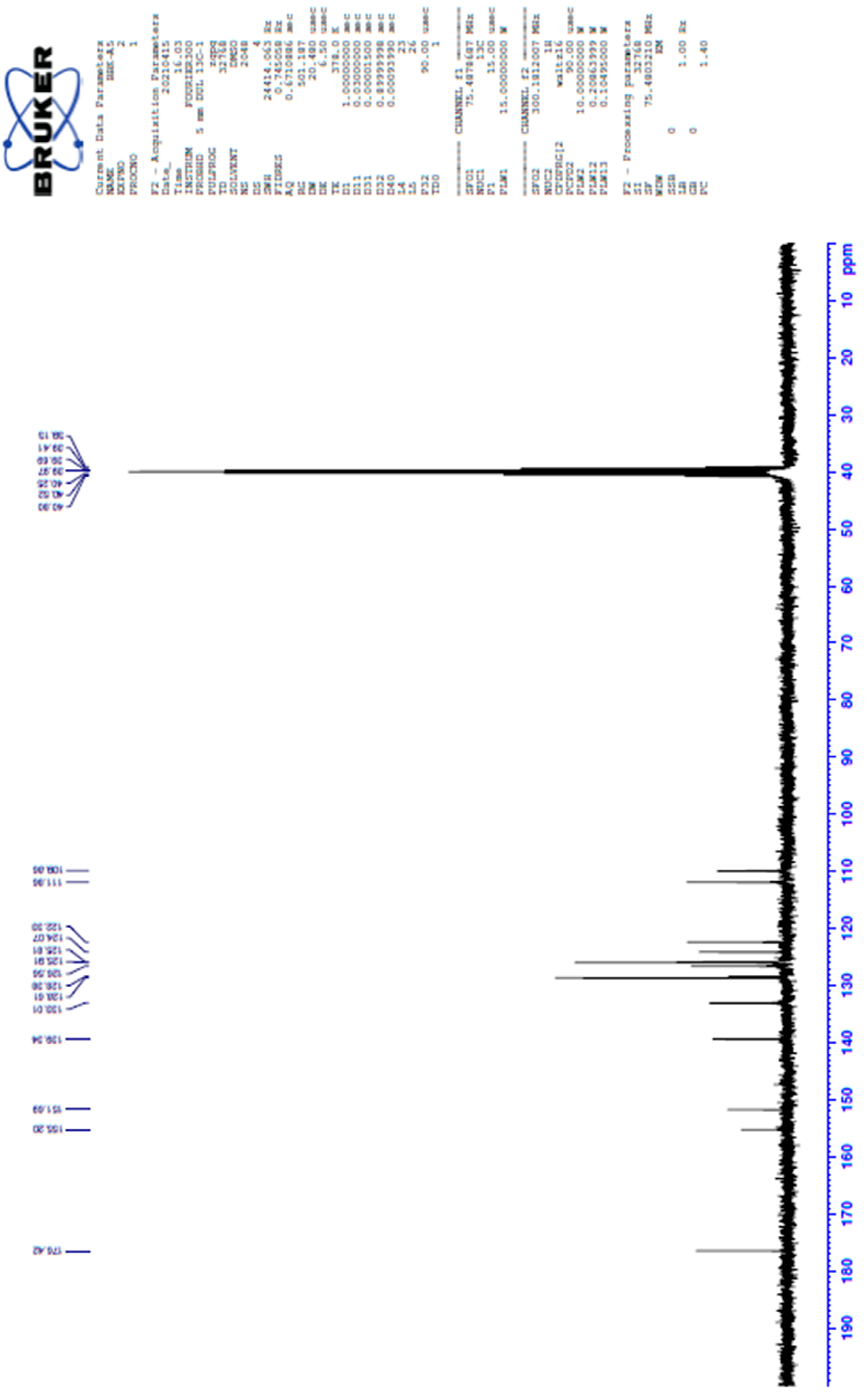

Figure S22: ^{13}C-NMR spectra of the compound **2e**

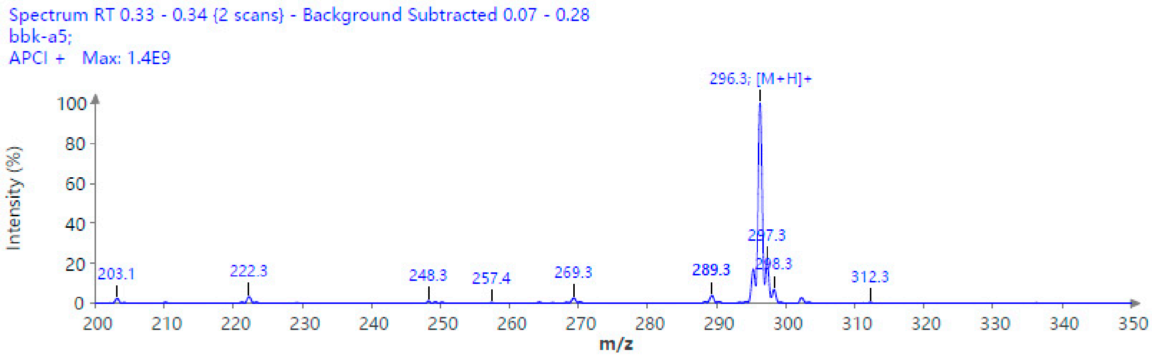

Figure S23: APCI-MS spectra of the compound **2e**

Formula Predictor Report - BBK-A5_55.lcd

Page 1 of 1

Data File: C:\LabSolutions\Data\Analiz\derya\BBK-A5_55.lcd

Elmt	Val.	Min	Max	Elmt	Val.	Min	Max	Elmt	Val.	Min	Max	Elmt	Val.	Min	Max	Use Adduct
H	1	0	40	O	2	0	6	S	2	1	1	Ru	2	0	0	H
C	4	0	40	F	1	0	0	Cl	1	0	0	Pd	2	0	0	
N	3	2	5	P	3	0	0	Br	1	0	0	I	3	0	0	

Error Margin (ppm):	10	DBE Range:	0.0 - 20.0	Electron Ions:	both
HC Ratio:	unlimited	Apply N Rule:	yes	Use MSn Info:	yes
Max Isotopes:	3	Isotope RI (%):	1.00	Isotope Res:	9000
MSn Iso RI (%):	10.00	MSn Logic Mode:	AND	Max Results:	150

Event#: 1 MS(E+) Ret. Time : 3.720 Scan# : 559

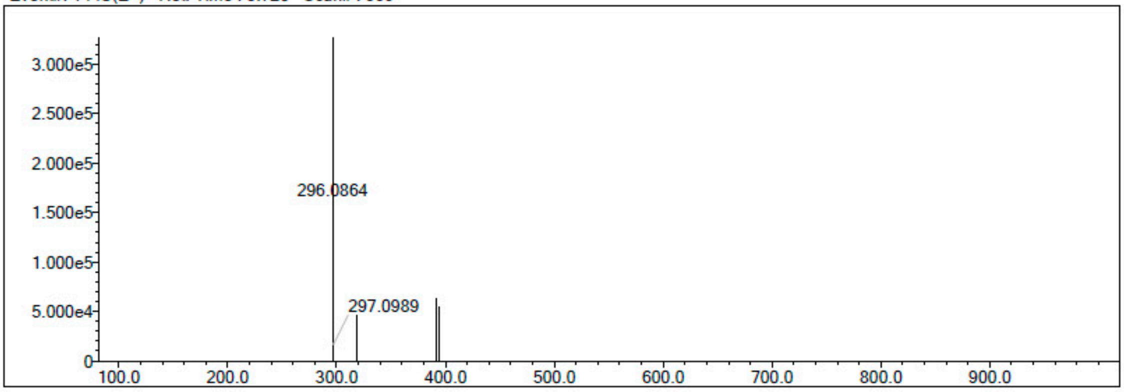

Measured region for 296.0864 m/z

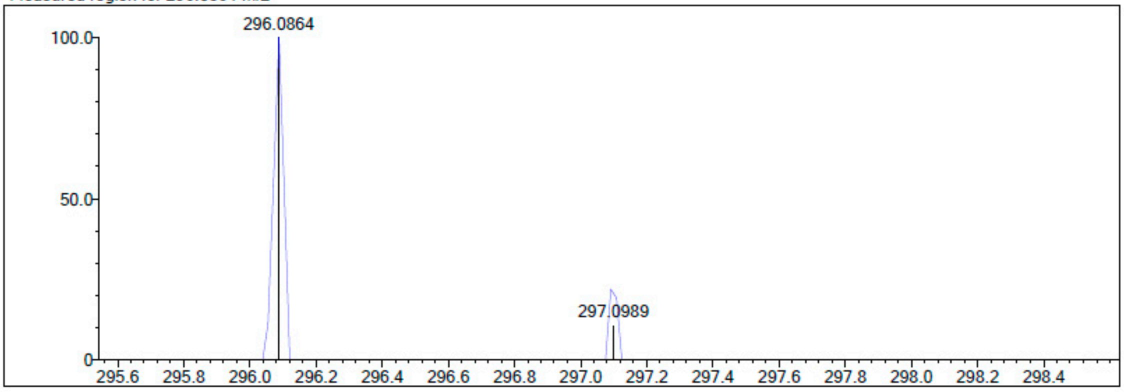

C16 H13 N3 O S [M+H]+ : Predicted region for 296.0852 m/z

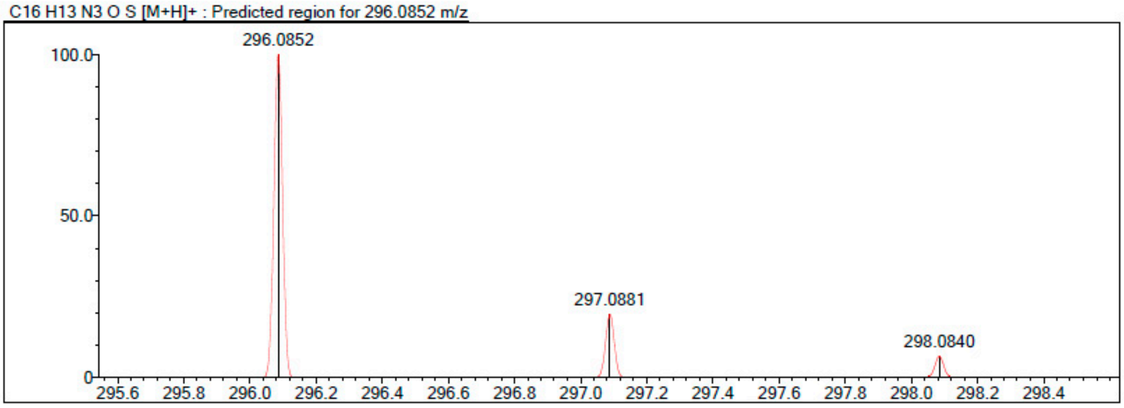

Rank	Score	Formula (M)	Ion	Meas. m/z	Pred. m/z	Df. (mDa)	Df. (ppm)	Iso	DBE
1	41.86	C16 H13 N3 O S	[M+H]+	296.0864	296.0852	1.2	4.05	45.32	12.0

Figure S24: HRMS spectra of the compound **2e**

==== Shimadzu LCMSsolution Analysis Report ====

Acquired by	: Admin
Sample Name	: BBK-A5
Sample ID	:
Vail #	: 65
Injection Volume	: 0.3 uL
Data File Name	: BBK-A5_55.lcd
Method File Name	: isocratic_serkan.lcm
Batch File Name	: batch.lcb
Report File Name	: DefaultLCMS.lcr
Data Acquired	: 10.08.2021 15:41:42
Data Processed	: 12.08.2021 14:15:32

<Chromatogram>

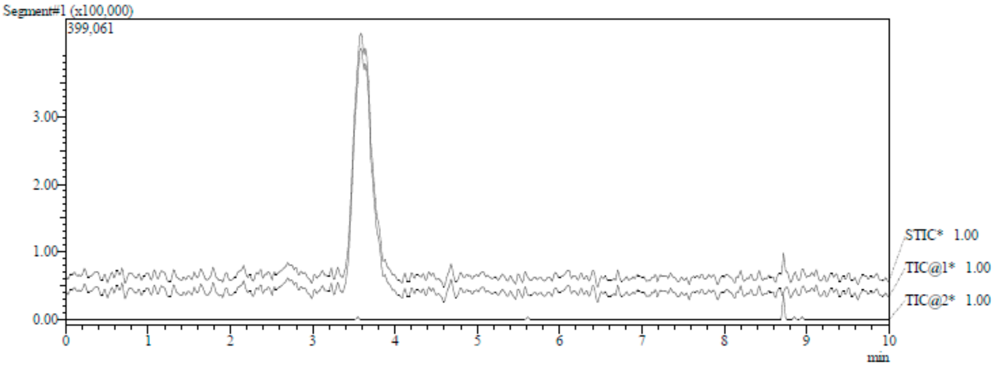

Chromatogram
BBK-A5 C:\LabSolutions\Data\Analiz\derya\BBK-A5_55.lcd

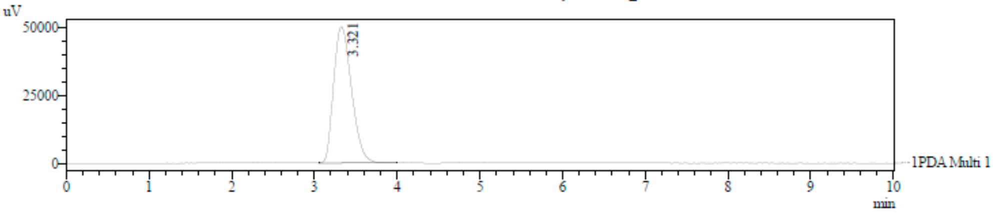

1 PDA Multi 1 / 341nm 4nm

PeakTable

PDA Ch1 341nm 4nm

Peak#	Ret. Time	Area	Height	Area %	Height %
1	3.321	759729	50462	100.000	100.000
Total		759729	50462	100.000	100.000

Figure S25: LCMS spectra of the compound **2e**

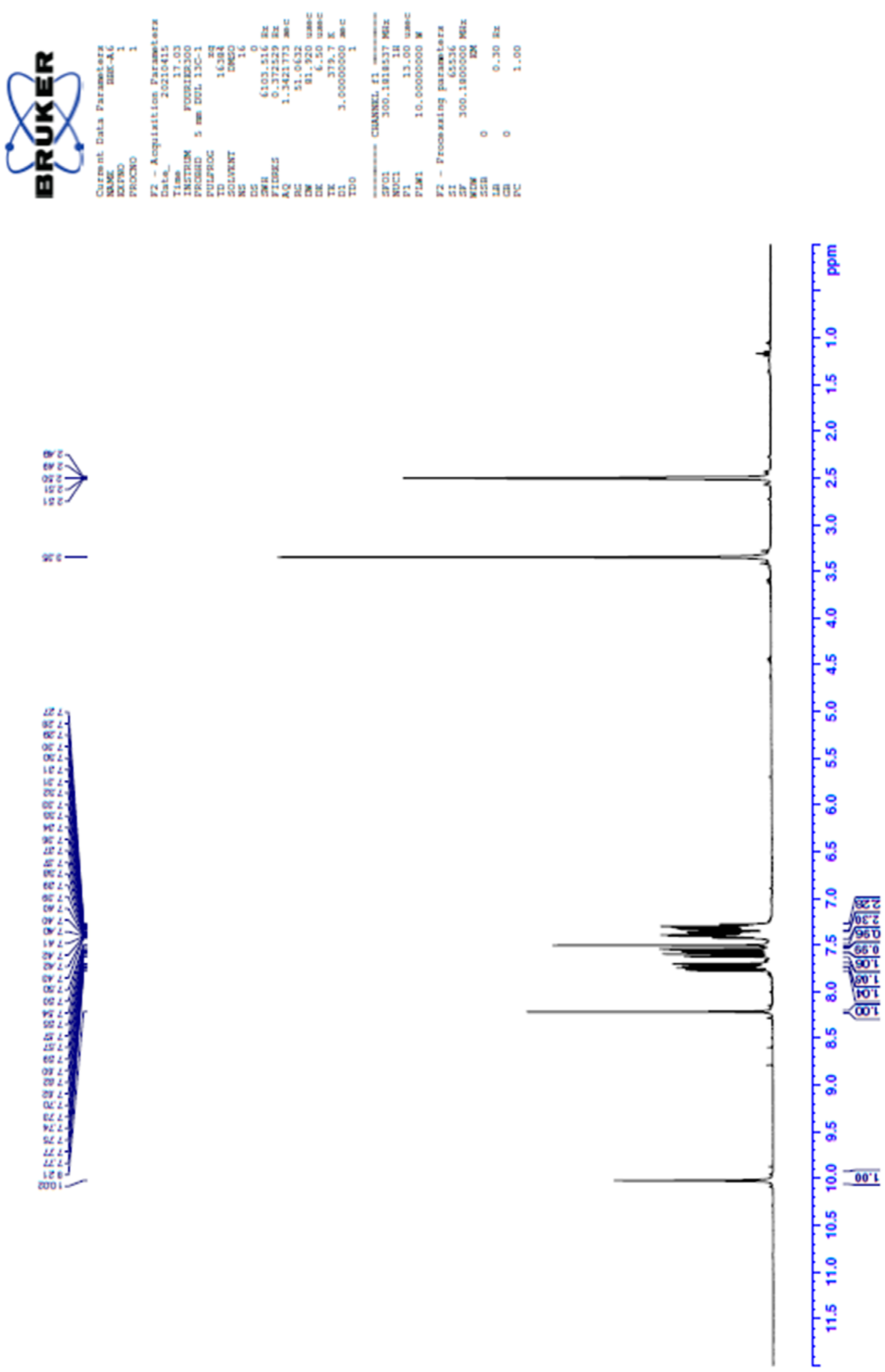

Figure S26: ^{1}H-NMR spectra of the compound **2f**

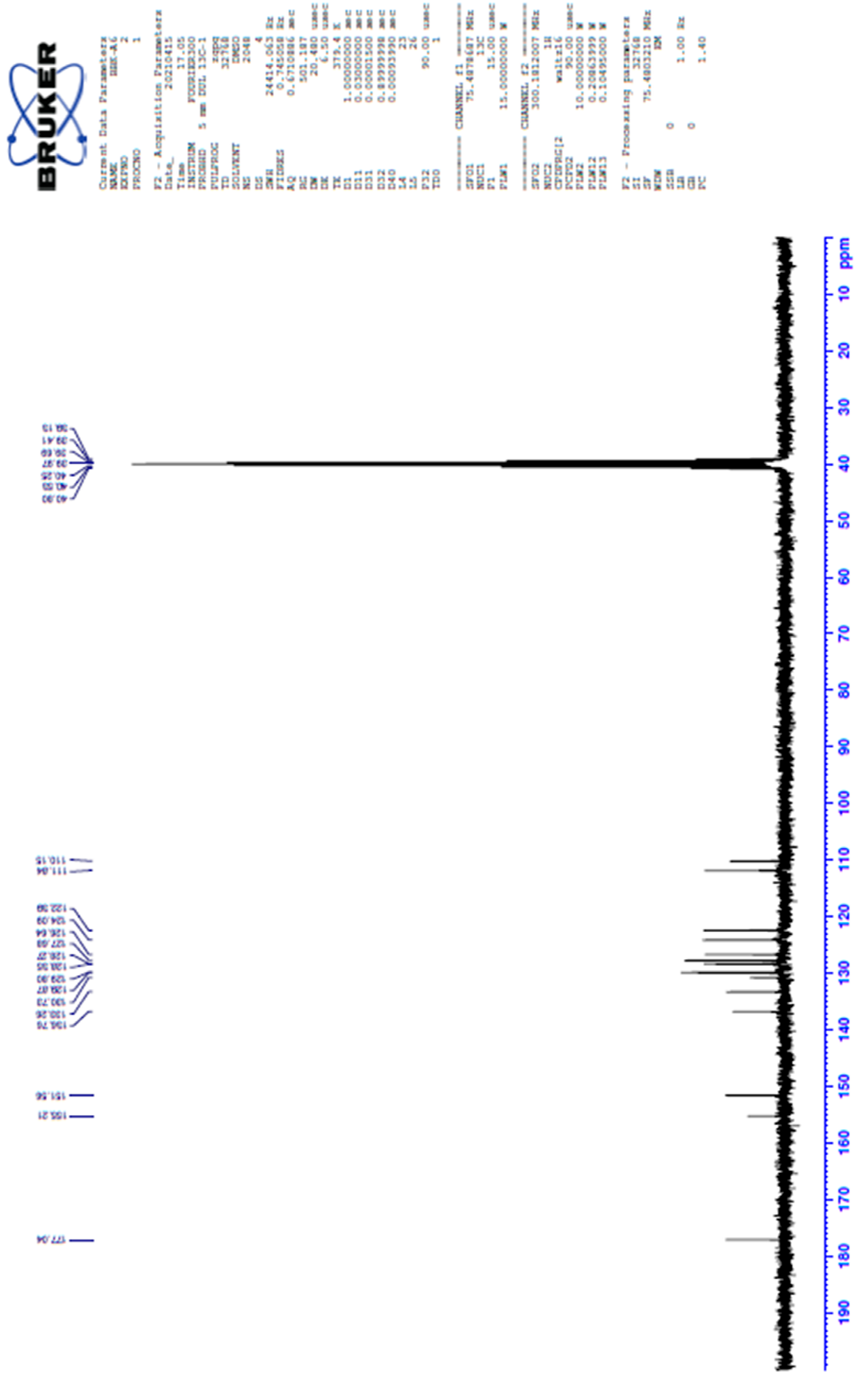

Figure S27: ^{13}C-NMR spectra of the compound **2f**

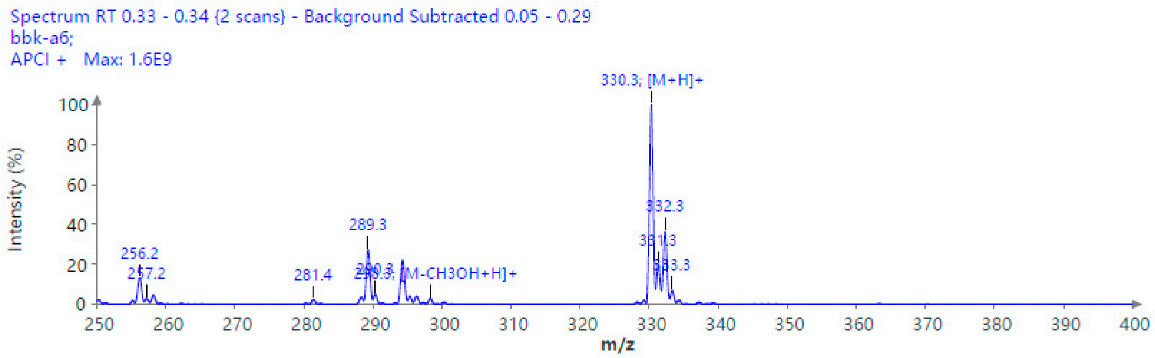

Figure S28: APCI-MS spectra of the compound **2f**

Formula Predictor Report - BBK-A6_56.lcd

Page 1 of 1

Data File: C:\LabSolutions\Data\Analiz\derya\BBK-A6_56.lcd

Elmt	Val.	Min	Max	Elmt	Val.	Min	Max	Elmt	Val.	Min	Max	Elmt	Val.	Min	Max	Use Adduct
H	1	0	40	O	2	0	6	S	2	1	1	Ru	2	0	0	H
C	4	0	40	F	1	0	0	Cl	1	1	1	Pd	2	0	0	
N	3	2	5	P	3	0	0	Br	1	0	0	I	3	0	0	

Error Margin (ppm):	10	DBE Range:	0.0 - 20.0	Electron Ions:	both
HC Ratio:	unlimited	Apply N Rule:	yes	Use MSn Info:	yes
Max Isotopes:	3	Isotope RI (%):	1.00	Isotope Res:	9000
MSn Iso RI (%):	10.00	MSn Logic Mode:	AND	Max Results:	150

Event#: 1 MS(E+) Ret. Time : 4.573 Scan# : 687

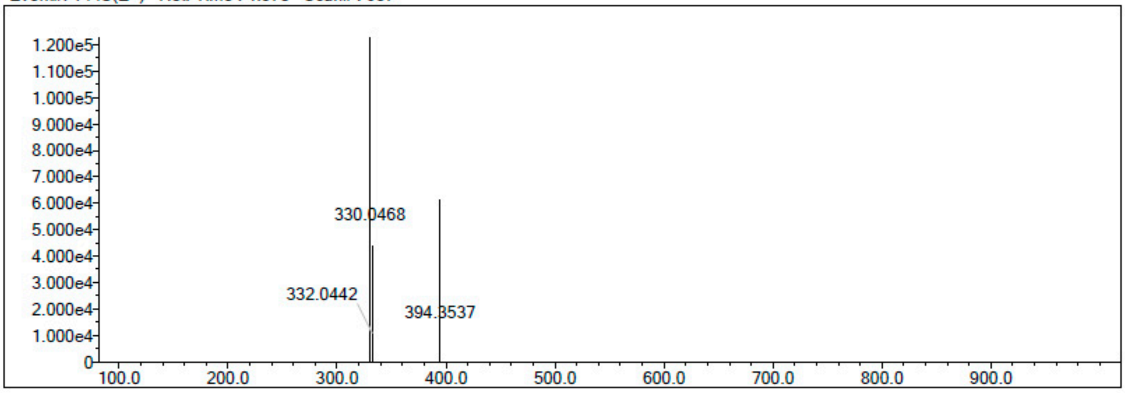

Measured region for 330.0468 m/z

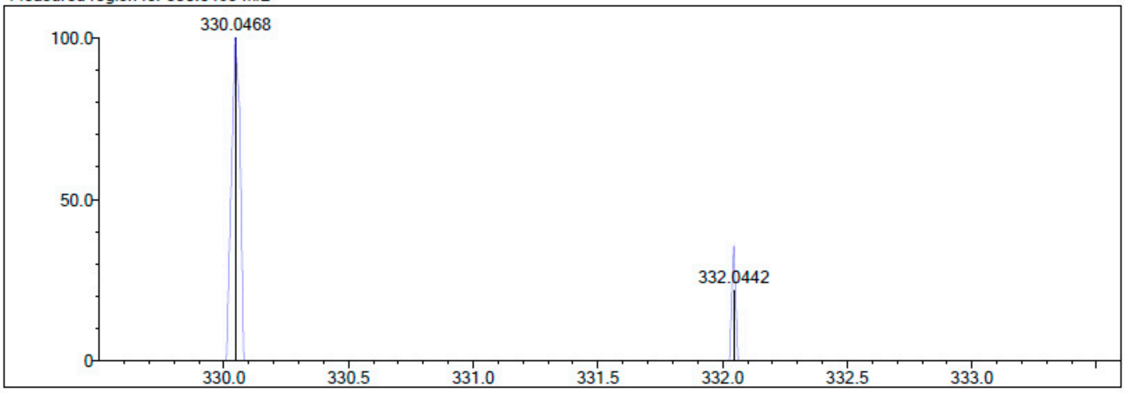

C16 H12 N3 O S Cl [M+H]+ : Predicted region for 330.0462 m/z

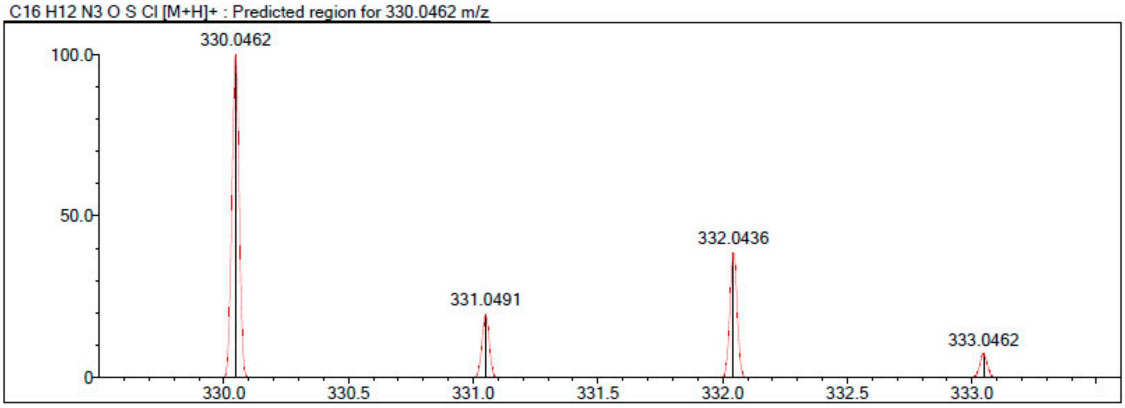

Rank	Score	Formula (M)	Ion	Meas. m/z	Pred. m/z	Df. (mDa)	Df. (ppm)	Iso	DBE
1	82.45	C16 H12 N3 O S Cl	[M+H]+	330.0468	330.0462	0.6	1.82	84.18	12.0

Figure S29: HRMS spectra of the compound **2f**

==== Shimadzu LCMSsolution Analysis Report ====

Acquired by	: Admin
Sample Name	: BBK-A6
Sample ID	:
Vail #	: 24
Injection Volume	: 0.3 uL
Data File Name	: BBK-A6_58.lcd
Method File Name	: isocratic_serkan.lcm
Batch File Name	: batch.lcb
Report File Name	: DefaultLCMS.lcr
Data Acquired	: 13.08.2021 11:00:47
Data Processed	: 13.08.2021 11:47:07

<Chromatogram>

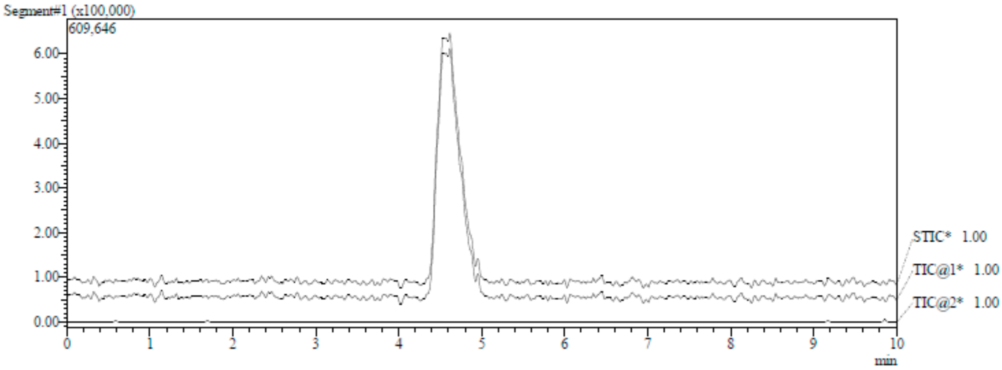

Chromatogram
BBK-A6 C:\LabSolutions\Data\Analiz\derya\BBK-A6_58.lcd

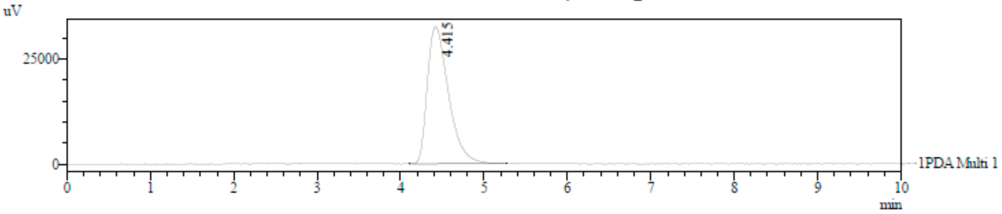

1 PDA Multi 1 / 341nm 4nm

PeakTable

PDA Ch1 341nm 4nm

Peak#	Ret. Time	Area	Height	Area %	Height %
1	4.415	578786	32721	100.000	100.000
Total		578786	32721	100.000	100.000

Figure S30: LCMS spectra of the compound **2f**

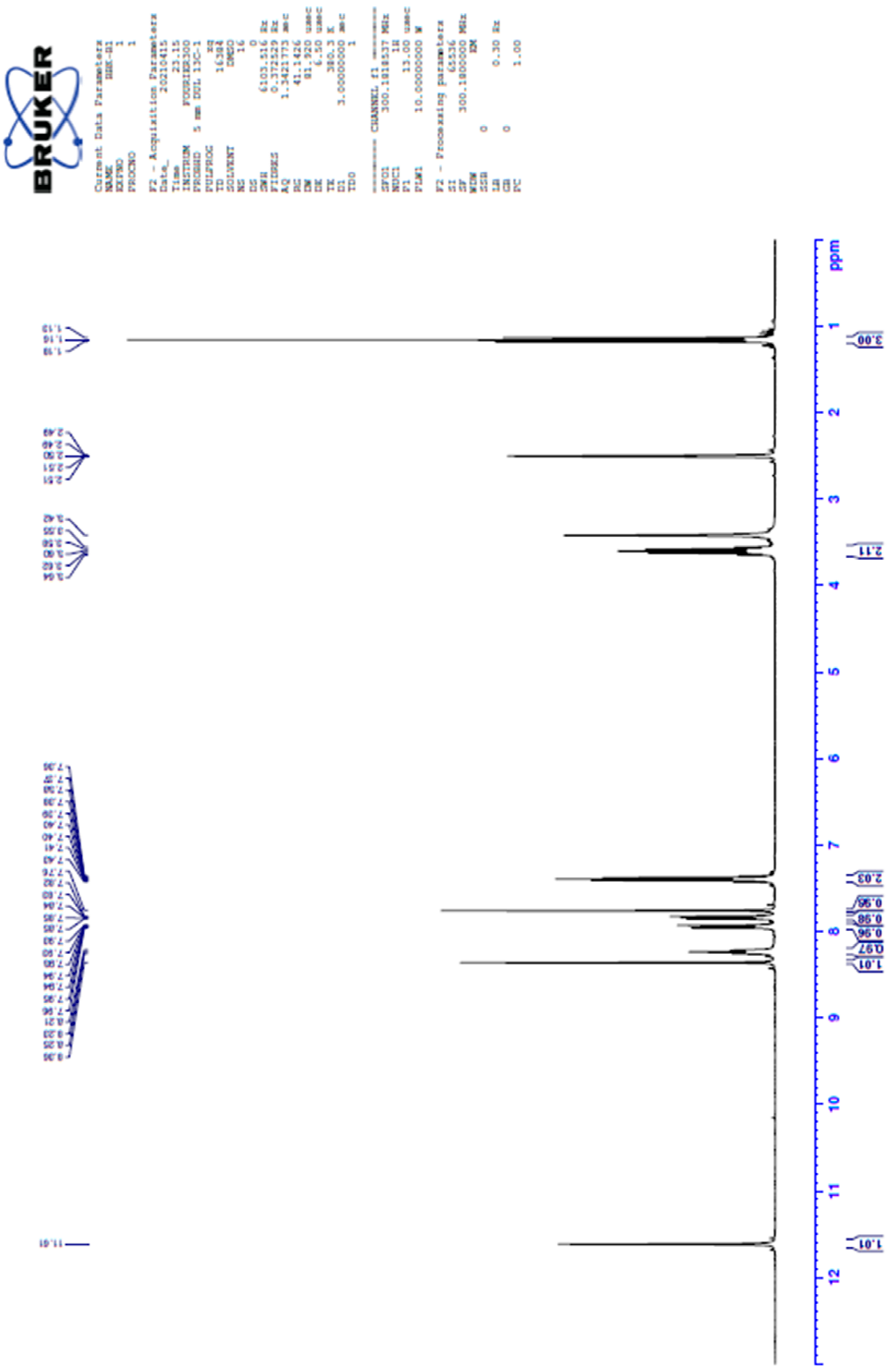

Figure S31: ^{1}H-NMR spectra of the compound **2g**

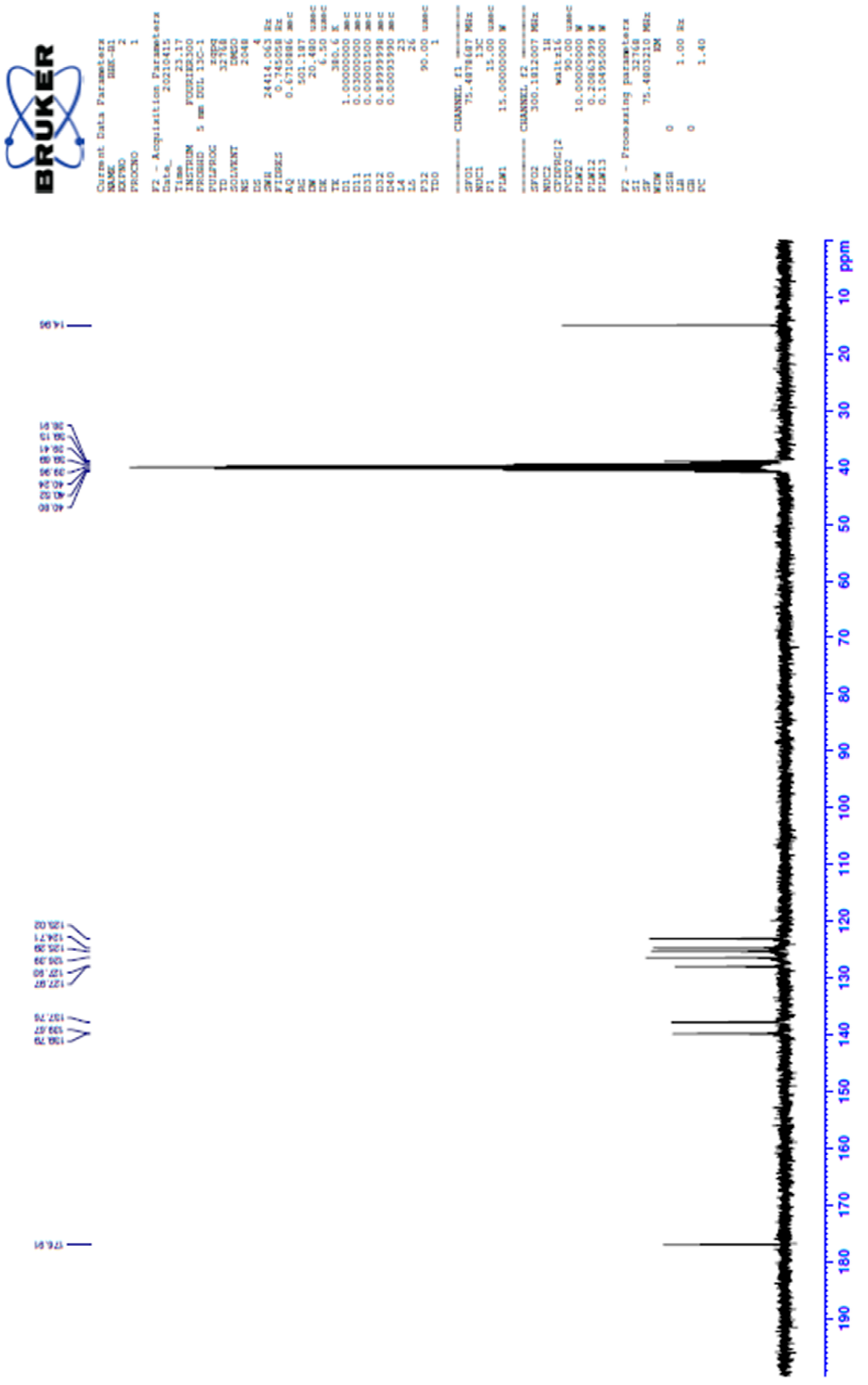

Figure S32: ^{13}C-NMR spectra of the compound **2g**

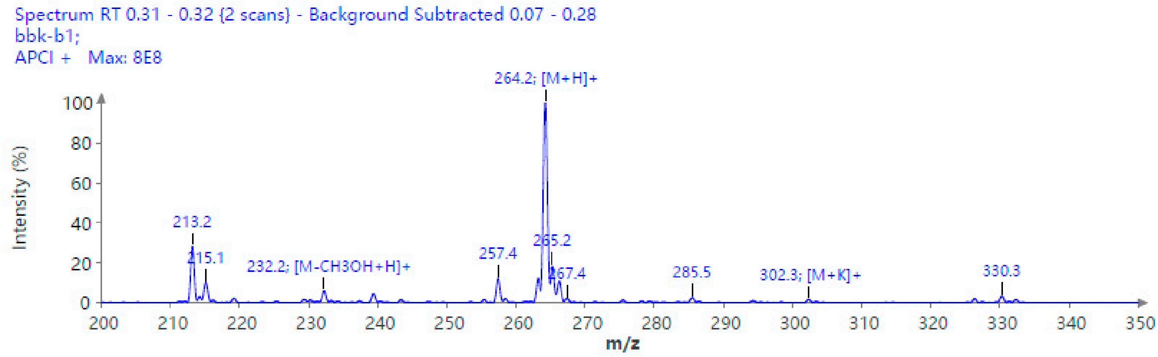

Figure S33: APCI-MS spectra of the compound **2g**

Data File: C:\LabSolutions\Data\Analiz\derya\BBK-B1_2.lcd

Elmt	Val.	Min	Max	Elmt	Val.	Min	Max	Elmt	Val.	Min	Max	Elmt	Val.	Min	Max	Use Adduct
H	1	0	40	O	2	0	4	S	2	1	2	Ru	2	0	0	H
C	4	0	40	F	1	0	0	Cl	1	0	1	Pd	2	0	0	
N	3	2	5	P	3	0	0	Br	1	0	0	I	3	0	0	

Error Margin (ppm): 10
HC Ratio: unlimited
Max Isotopes: 3
MSn Iso RI (%): 10.00

DBE Range: 6.0 - 12.0
Apply N Rule: yes
Isotope RI (%): 1.00
MSn Logic Mode: AND

Electron Ions: both
Use MSn Info: yes
Isotope Res: 9000
Max Results: 150

Event#: 1 MS(E+) Ret. Time : 3.120 Scan# : 469

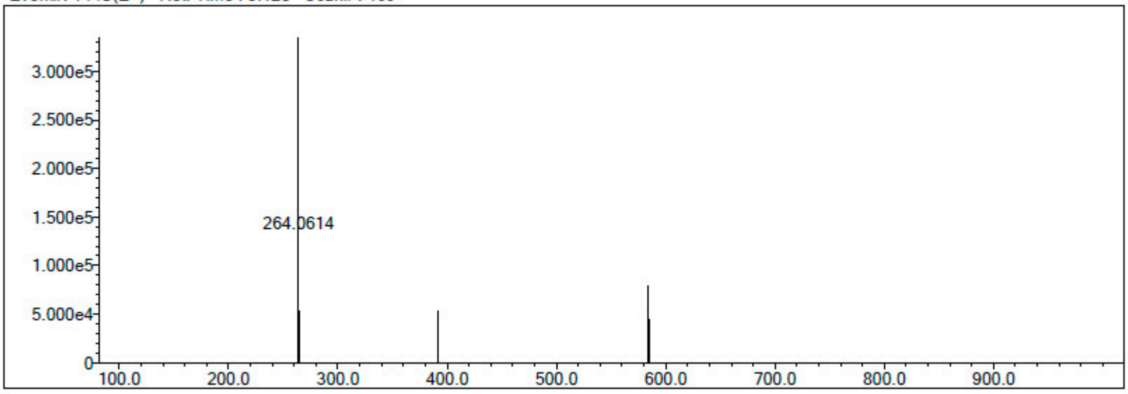

Measured region for 264.0614 m/z

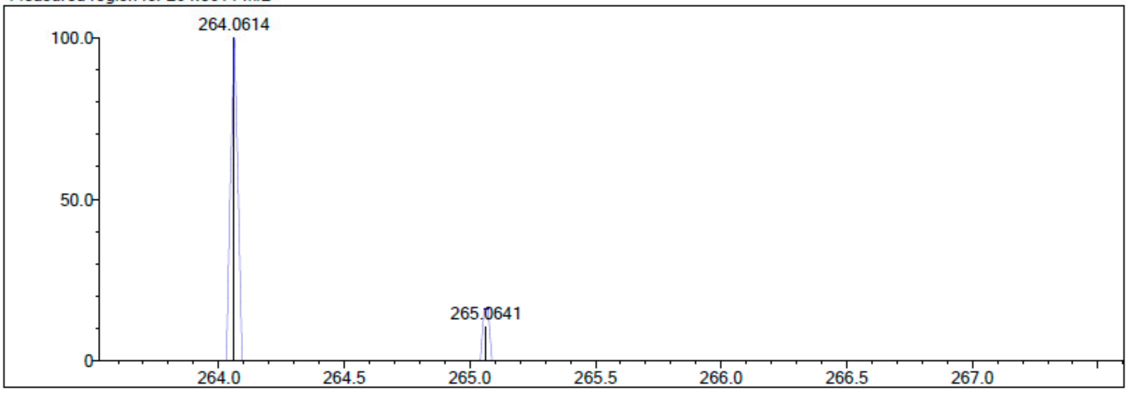

C12 H13 N3 S2 [M+H]+ : Predicted region for 264.0624 m/z

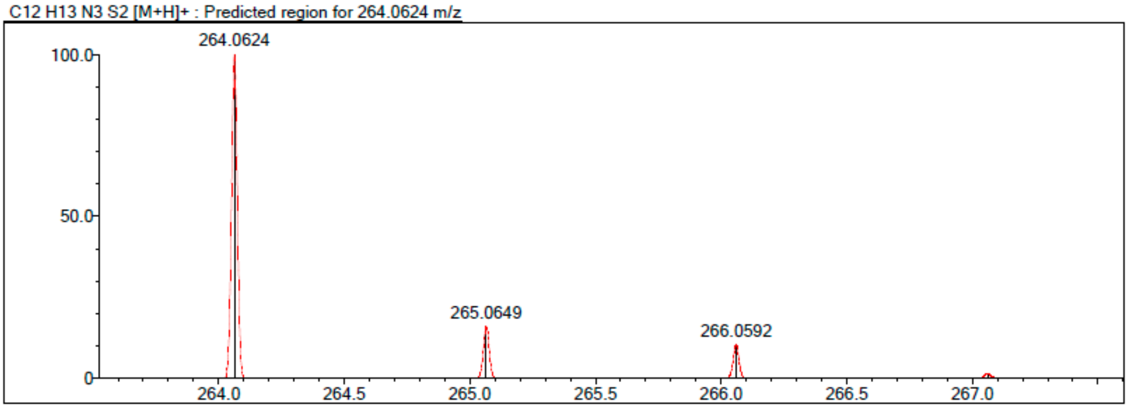

Rank	Score	Formula (M)	Ion	Meas. m/z	Pred. m/z	Df. (mDa)	Df. (ppm)	Iso	DBE
1	0.00	C12 H13 N3 S2	[M+H]+	264.0614	264.0624	-1.0	-3.79	0.00	8.0

Figure S34: HRMS spectra of the compound **2g**

==== Shimadzu LCMSsolution Analysis Report ====

Acquired by : Admin
Sample Name : BBK-B1
Sample ID :
Vail # : 67
Injection Volume : 0.3 uL
Data File Name : BBK-B1_59.lcd
Method File Name : isocratic_serkan.lcm
Batch File Name : batch.lcb
Report File Name : DefaultLCMS.lcr
Data Acquired : 10.08.2021 16:13:22
Data Processed : 12.08.2021 14:14:24

<Chromatogram>

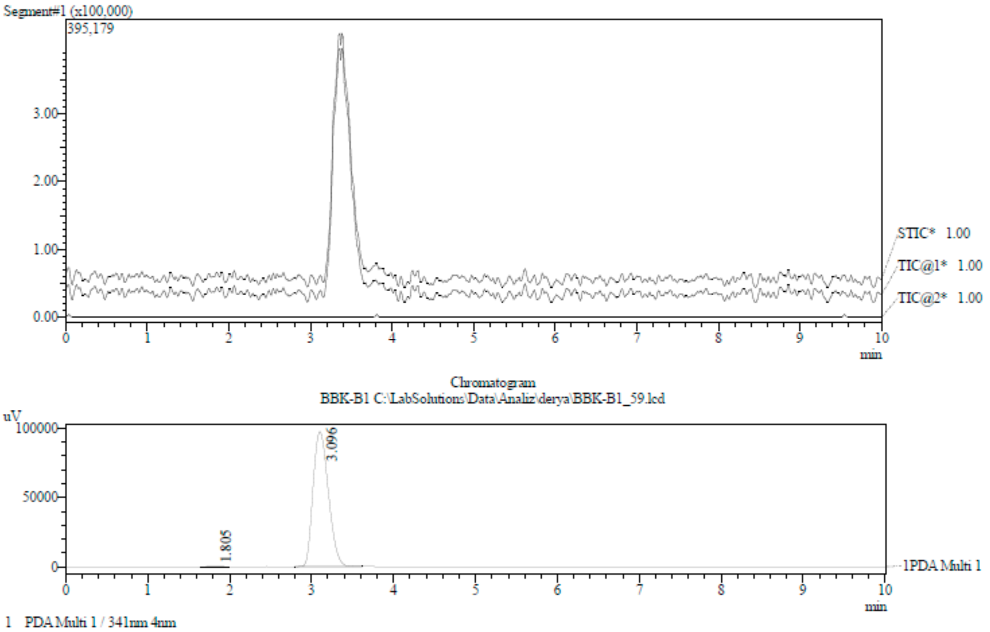

1 PDA Multi 1 / 341nm 4nm

PeakTable

PDA Ch1 341nm 4nm

Peak#	Ret. Time	Area	Height	Area %	Height %
1	1.805	2309	206	0.182	0.211
2	3.096	1269972	97566	99.818	99.789
Total		1272282	97772	100.000	100.000

Figure S35: LCMS spectra of the compound **2g**

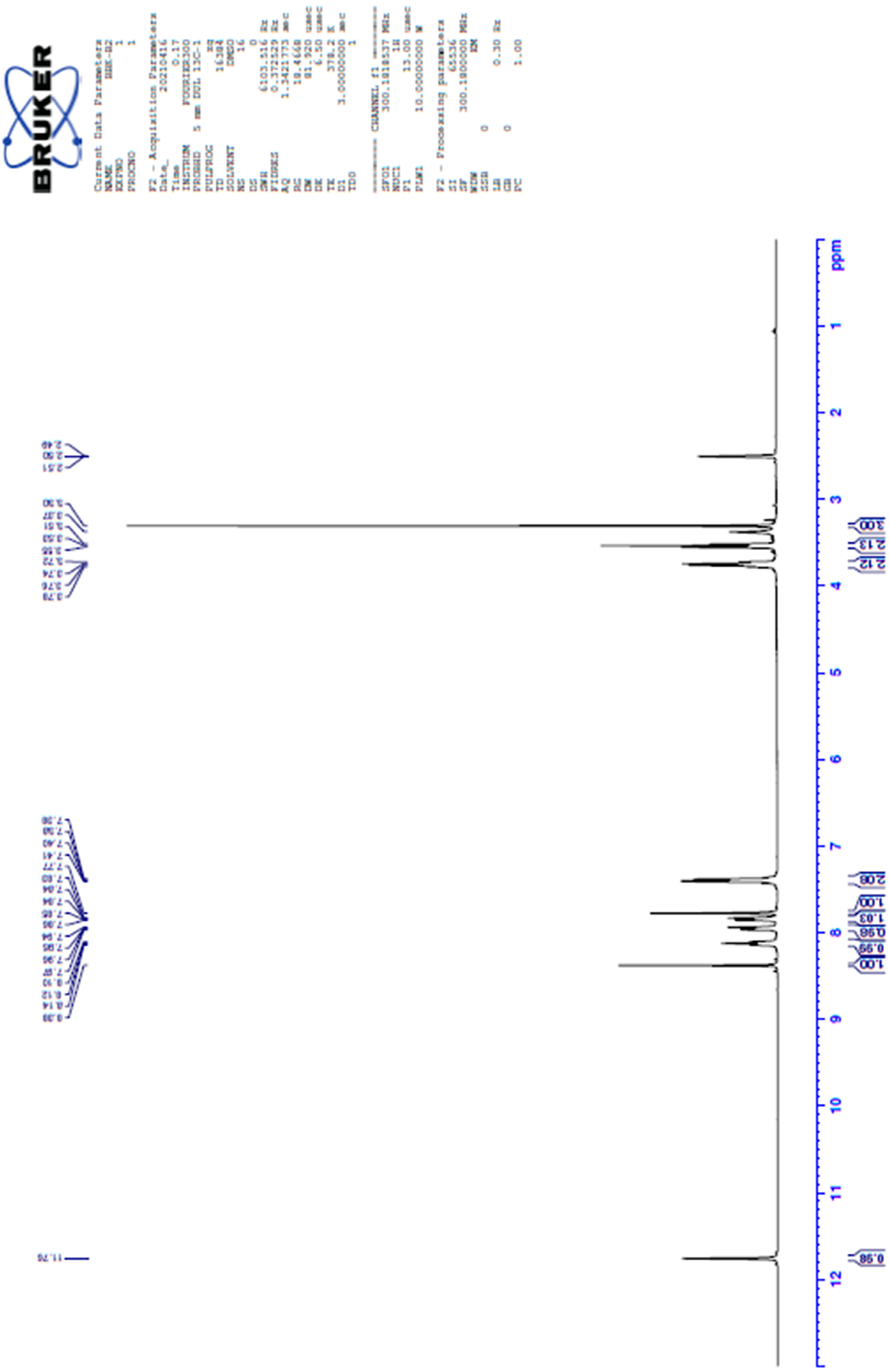

Figure S36: ^{1}H-NMR spectra of the compound **2h**

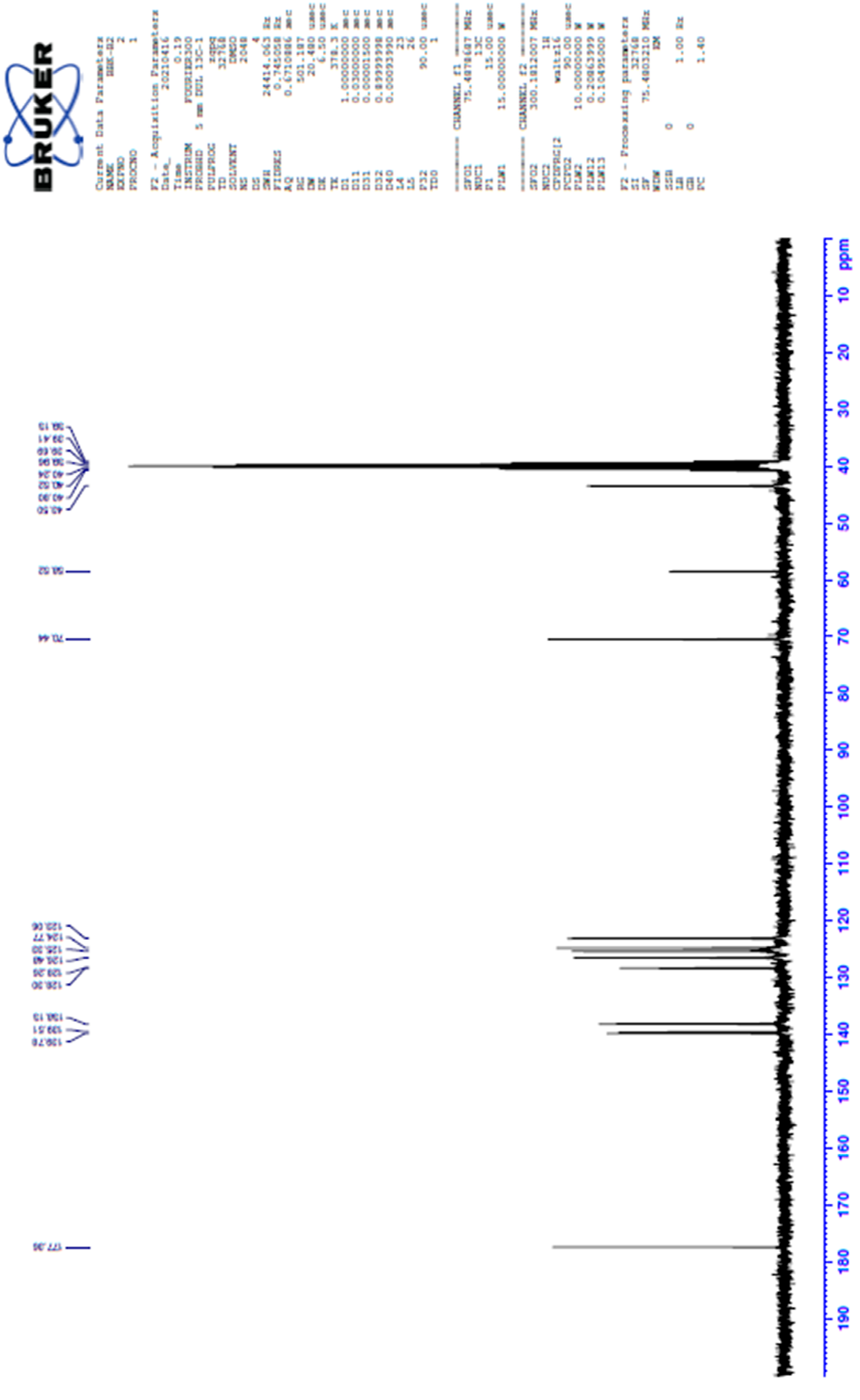

Figure S37: ^{13}C-NMR spectra of the compound **2h**

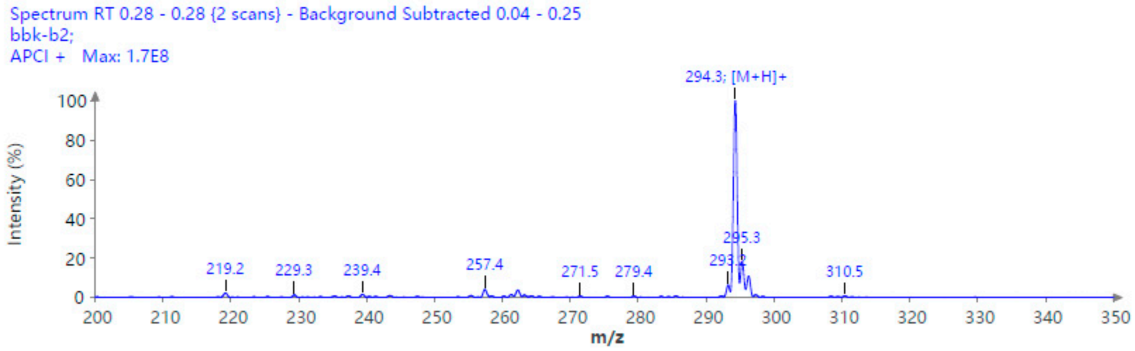

Figure S38: APCI-MS spectra of the compound **2h**

Data File: C:\LabSolutions\Data\Analiz\derya\BBK-B2_3.lcd

Elmt	Val.	Min	Max	Elmt	Val.	Min	Max	Elmt	Val.	Min	Max	Elmt	Val.	Min	Max	Use Adduct
H	1	0	40	O	2	0	4	S	2	1	2	Ru	2	0	0	H
C	4	0	40	F	1	0	0	Cl	1	0	1	Pd	2	0	0	
N	3	2	5	P	3	0	0	Br	1	0	0	I	3	0	0	

Error Margin (ppm): 10
HC Ratio: unlimited
Max Isotopes: 3
MSn Iso RI (%): 10.00

DBE Range: 6.0 - 12.0
Apply N Rule: yes
Isotope RI (%): 1.00
MSn Logic Mode: AND

Electron Ions: both
Use MSn Info: yes
Isotope Res: 9000
Max Results: 150

Event#: 1 MS(E+) Ret. Time : 3.120 Scan# : 469

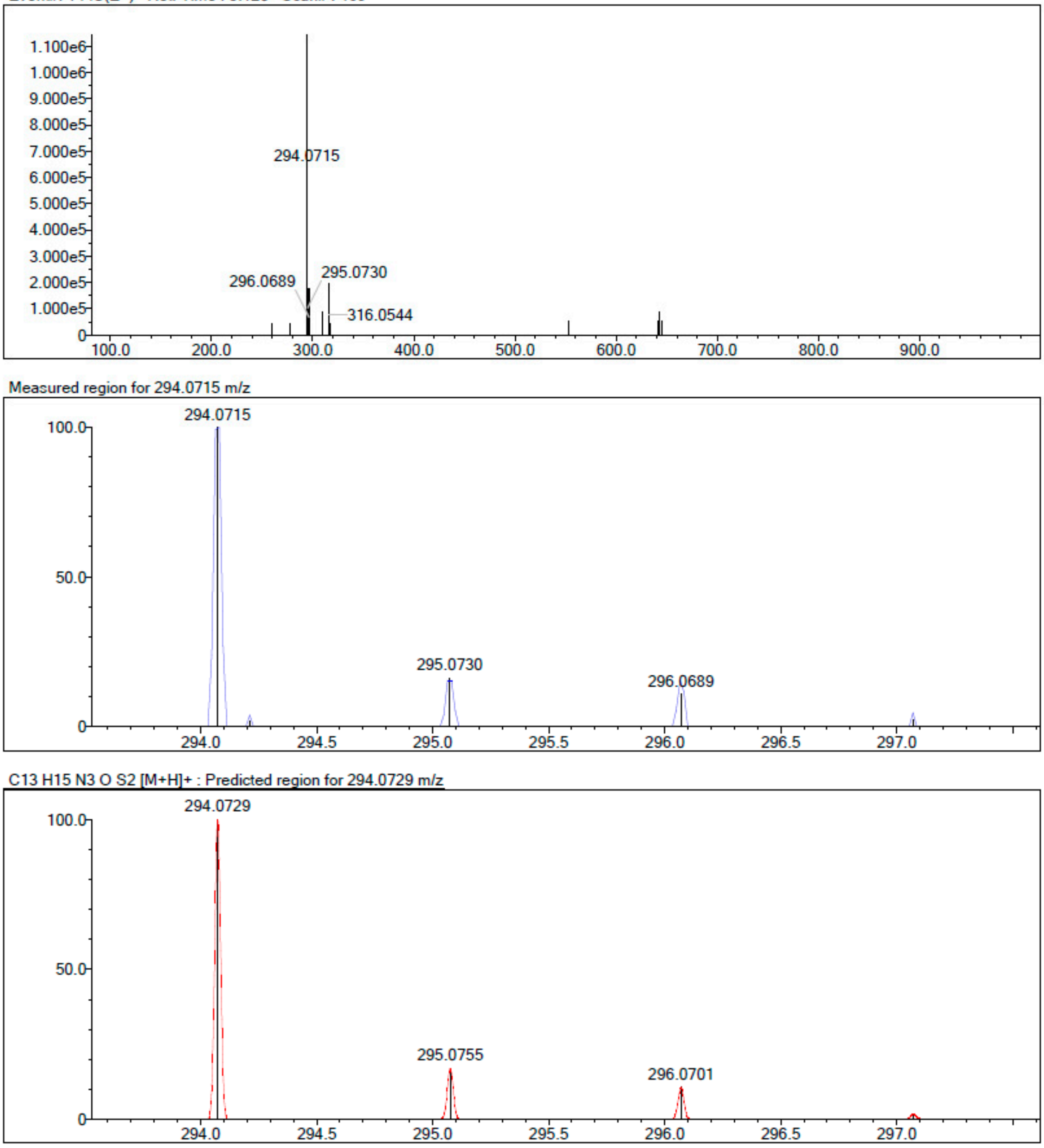

Rank	Score	Formula (M)	Ion	Meas. m/z	Pred. m/z	Df. (mDa)	Df. (ppm)	Iso	DBE
1	76.75	C13 H15 N3 O S2	[M+H]+	294.0715	294.0729	-1.4	-4.76	84.71	8.0

Figure S39: HRMS spectra of the compound **2h**

==== Shimadzu LCMSsolution Analysis Report ====

Acquired by	: Admin
Sample Name	: BBK-B2
Sample ID	:
Vail #	: 68
Injection Volume	: 0.3 uL
Data File Name	: BBK-B2_60.lcd
Method File Name	: isocratic_serkan.lcm
Batch File Name	: batch.lcb
Report File Name	: DefaultLCMS.lcr
Data Acquired	: 10.08.2021 16:23:55
Data Processed	: 12.08.2021 14:14:03

<Chromatogram>

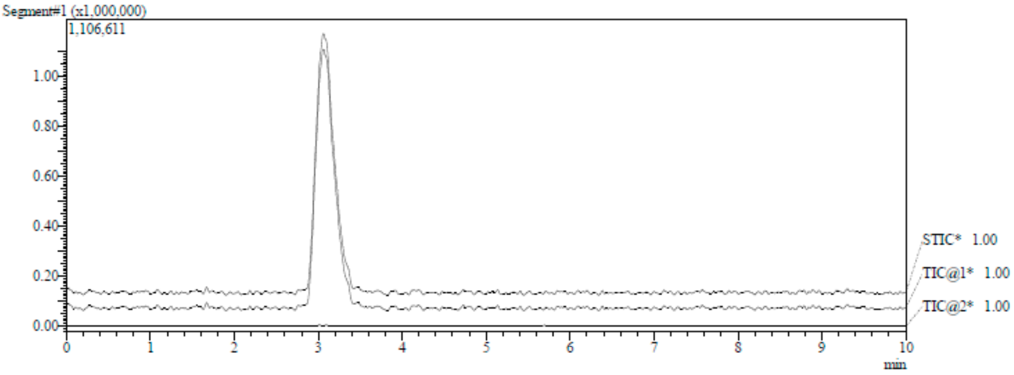

Chromatogram
BBK-B2 C:\LabSolutions\Data\Analiz\derya\BBK-B2_60.lcd

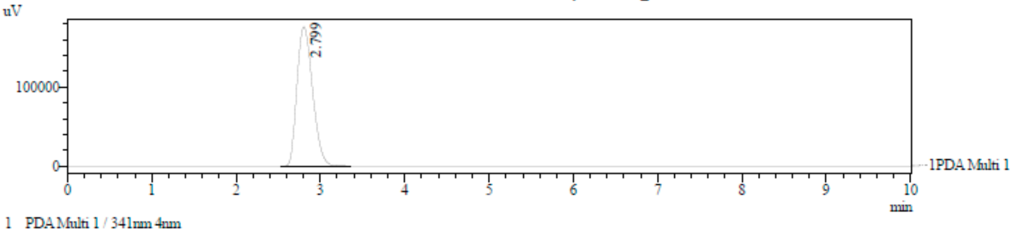

1 PDA Multi 1 / 341nm 4nm

PeakTable

PDA Ch1 341nm 4nm

Peak#	Ret. Time	Area	Height	Area %	Height %
1	2.799	2267910	176620	100.000	100.000
Total		2267910	176620	100.000	100.000

Figure S40: LCMS spectra of the compound **2h**

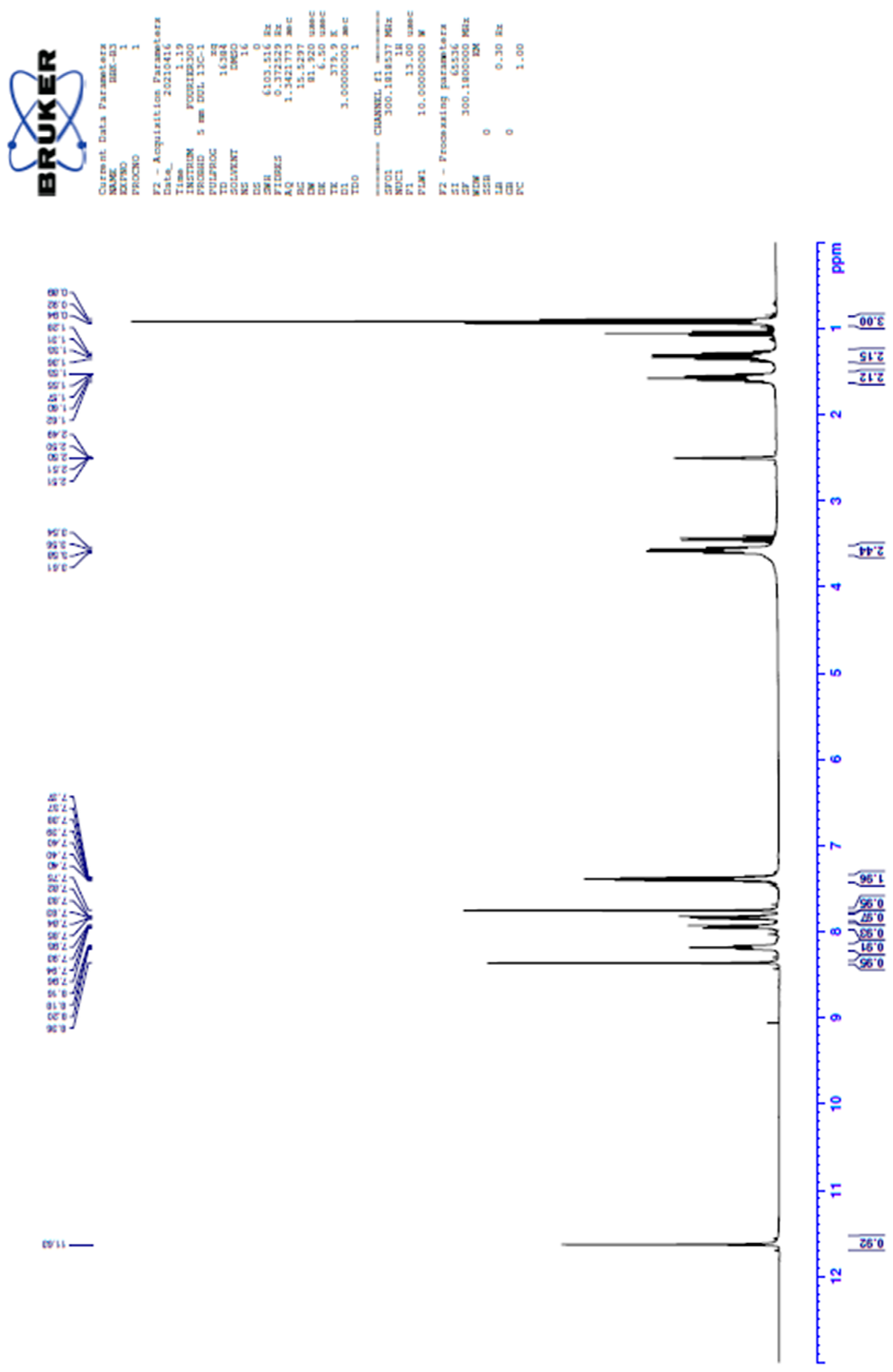

Figure S41: ^{1}H-NMR spectra of the compound **2i**

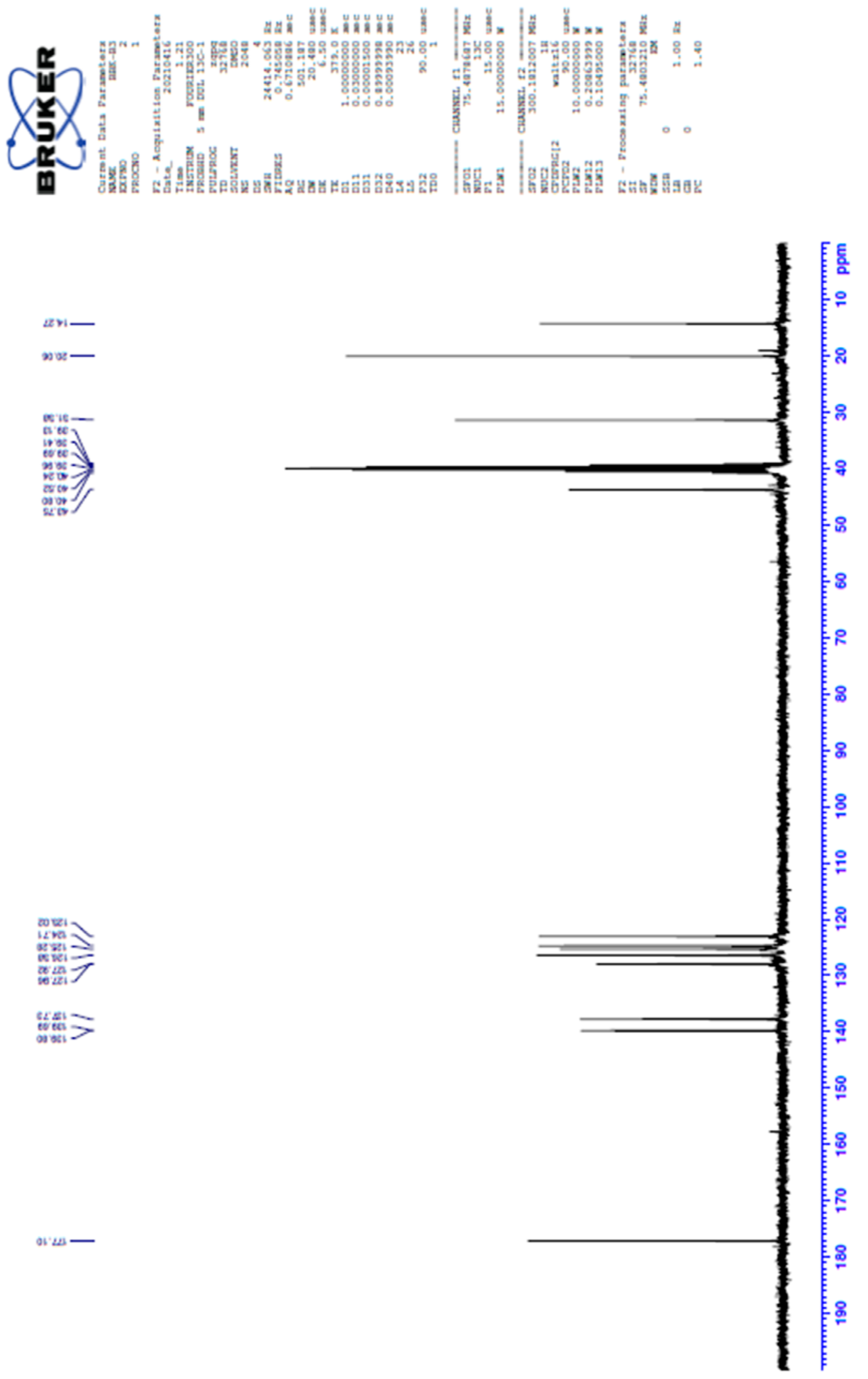

Figure S42: ^{13}C-NMR spectra of the compound **2i**

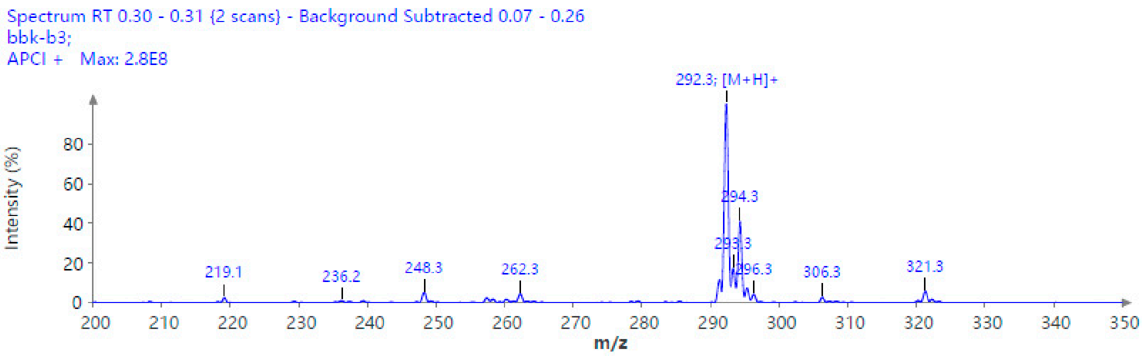

Figure S43: APCI-MS spectra of the compound **2i**

Data File: C:\LabSolutions\Data\Analiz\derya\BBK-B3_4.lcd

Elmt	Val.	Min	Max	Elmt	Val.	Min	Max	Elmt	Val.	Min	Max	Elmt	Val.	Min	Max	Use Adduct
H	1	0	40	O	2	0	4	S	2	1	2	Ru	2	0	0	H
C	4	0	40	F	1	0	0	Cl	1	0	1	Pd	2	0	0	
N	3	2	5	P	3	0	0	Br	1	0	0	I	3	0	0	

Error Margin (ppm):	10	DBE Range:	6.0 - 12.0	Electron Ions:	both
HC Ratio:	unlimited	Apply N Rule:	yes	Use MSn Info:	yes
Max Isotopes:	3	Isotope RI (%):	1.00	Isotope Res:	9000
MSn Iso RI (%):	10.00	MSn Logic Mode:	AND	Max Results:	150

Event#: 1 MS(E+) Ret. Time : 5.293 Scan# : 795

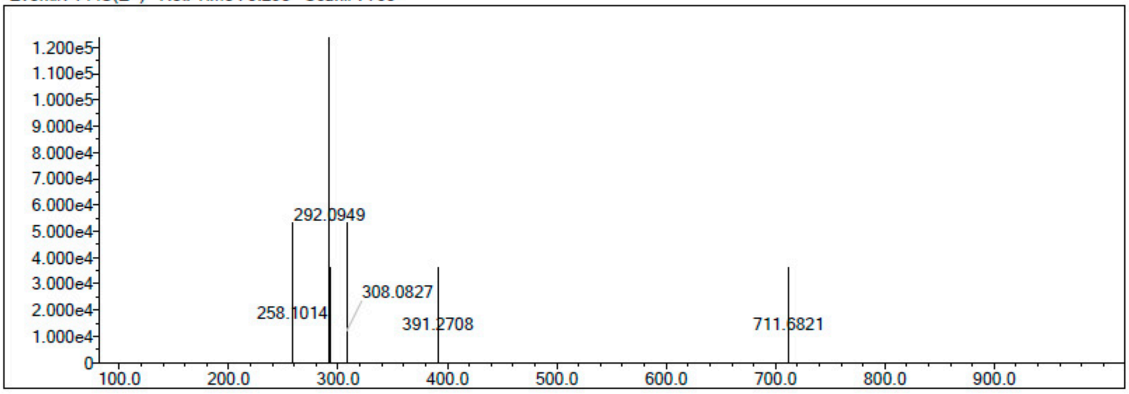

Measured region for 292.0949 m/z

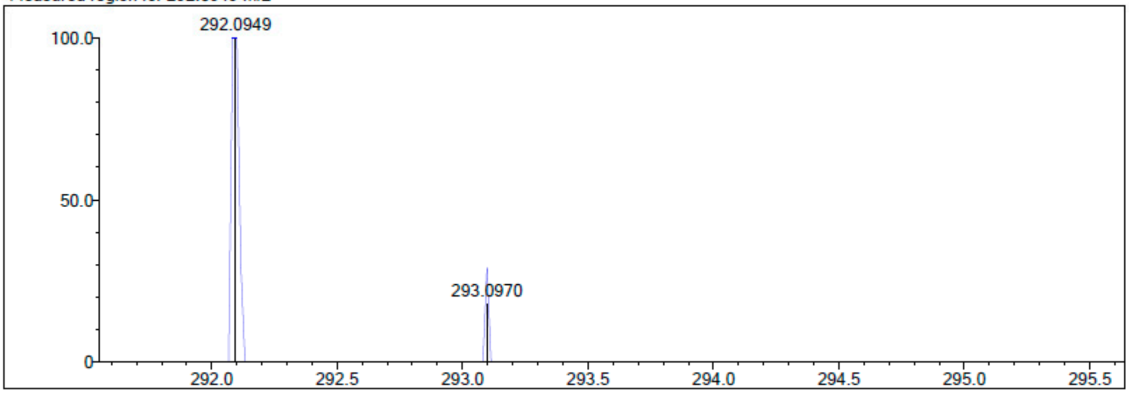

C14 H17 N3 S2 [M+H]+ : Predicted region for 292.0937 m/z

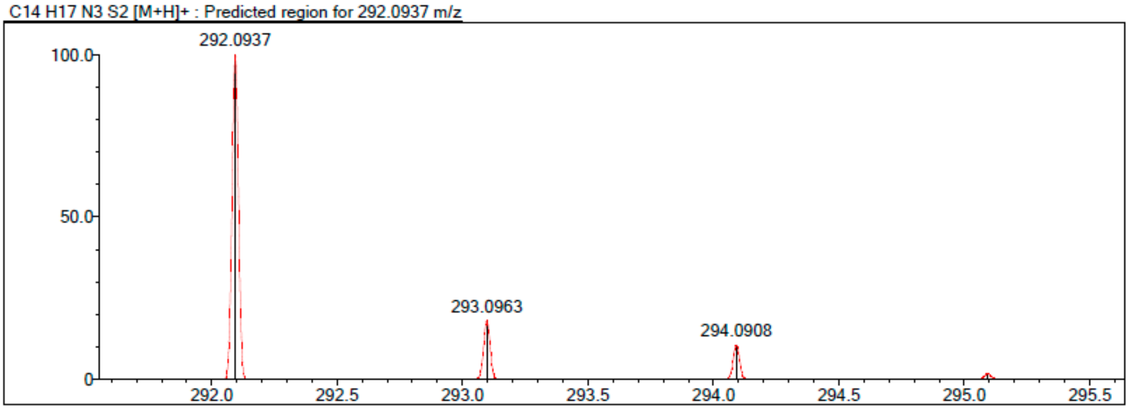

Rank	Score	Formula (M)	Ion	Meas. m/z	Pred. m/z	Df. (mDa)	Df. (ppm)	Iso	DBE
1	0.00	C14 H17 N3 S2	[M+H]+	292.0949	292.0937	1.2	4.11	0.00	8.0

Figure S44: HRMS spectra of the compound **2i**

==== Shimadzu LCMSsolution Analysis Report ====

Acquired by	: Admin
Sample Name	: BBK-B3
Sample ID	:
Vail #	: 69
Injection Volume	: 0.3 uL
Data File Name	: BBK-B3_61.lcd
Method File Name	: isocratic_serkan.lcm
Batch File Name	: batch.lcb
Report File Name	: DefaultLCMS.lcr
Data Acquired	: 10.08.2021 16:34:29
Data Processed	: 12.08.2021 14:13:26

<Chromatogram>

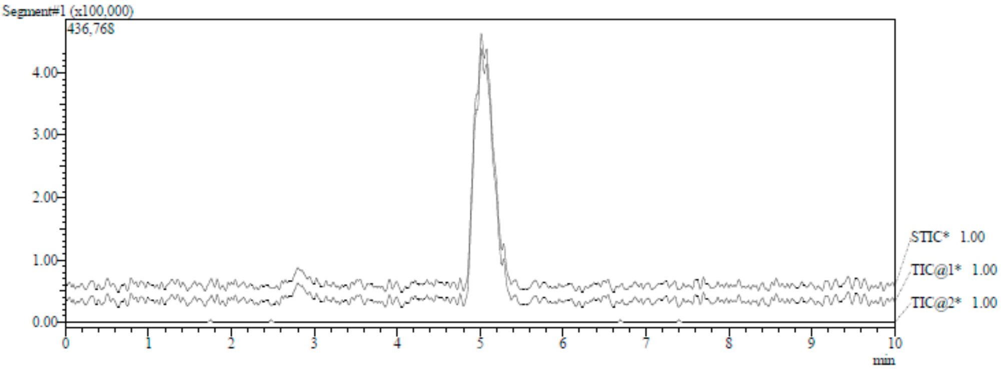

Chromatogram
BBK-B3 C:\LabSolutions\Data\Analiz\derya\BBK-B3_61.lcd

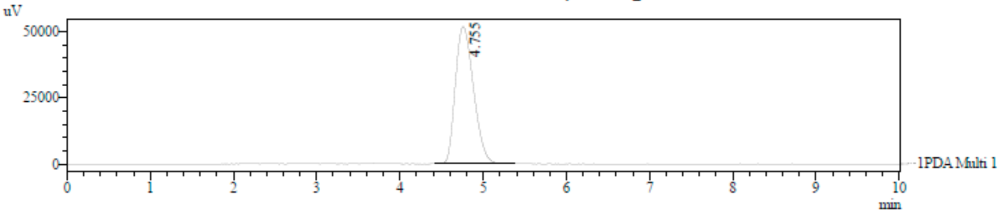

1 PDA Multi 1 / 341nm 4nm

PeakTable

PDA Ch1 341nm 4nm

Peak#	Ret. Time	Area	Height	Area %	Height %
1	4.755	790811	51986	100.000	100.000
Total		790811	51986	100.000	100.000

Figure S45: LCMS spectra of the compound **2i**

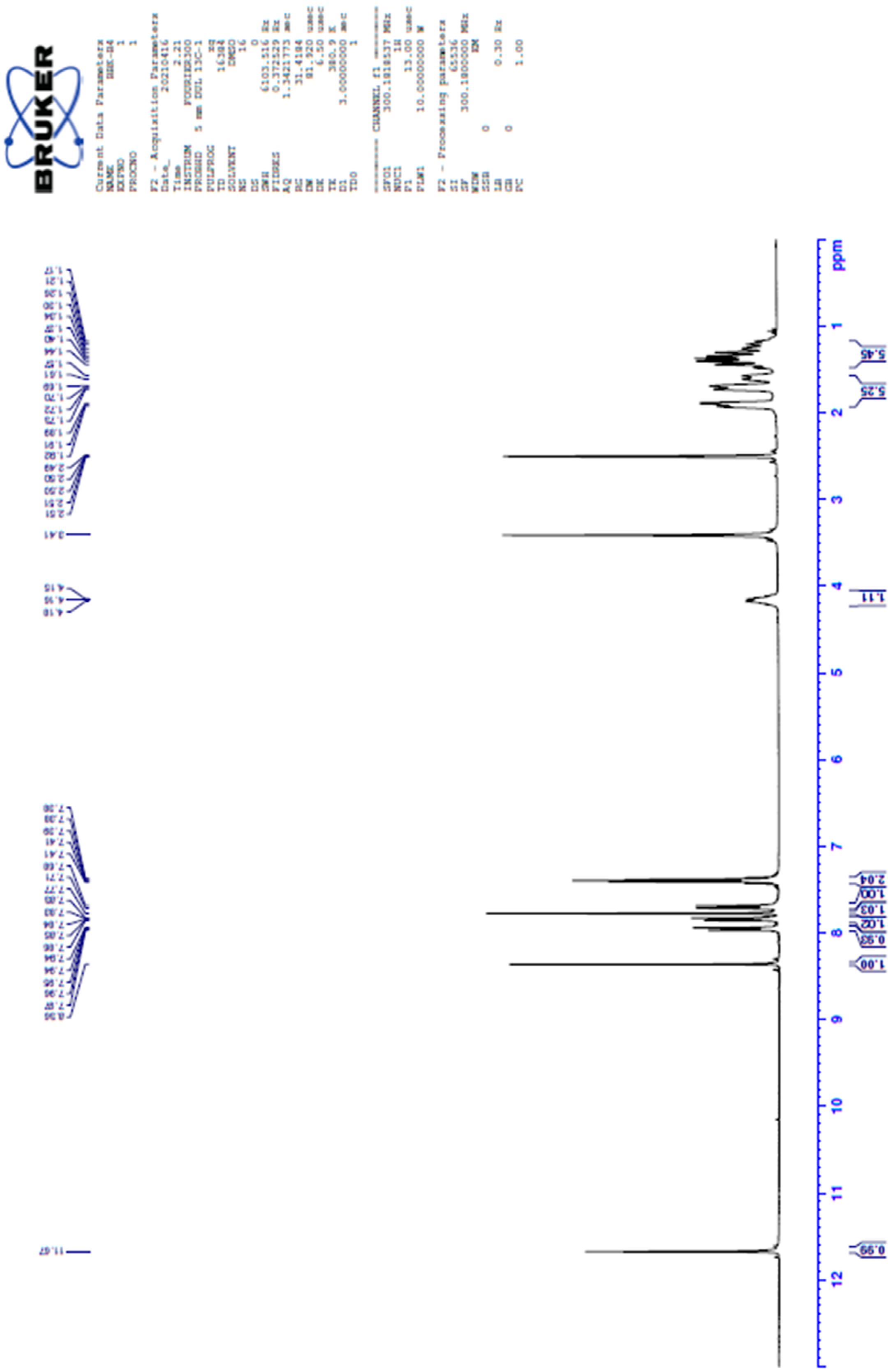

Figure S46: ^{1}H-NMR spectra of the compound **2j**

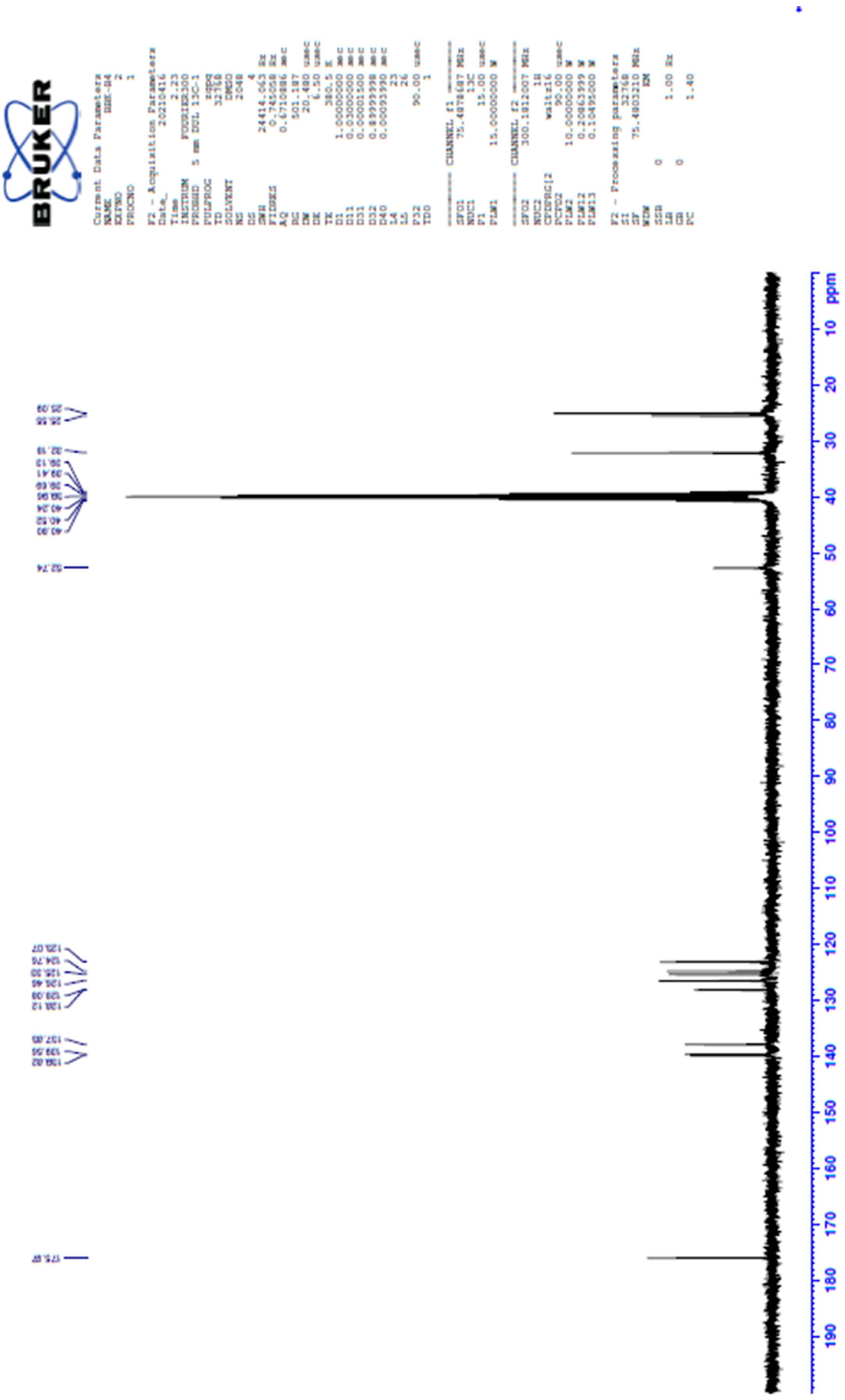

Figure S47: ^{13}C-NMR spectra of the compound **2j**

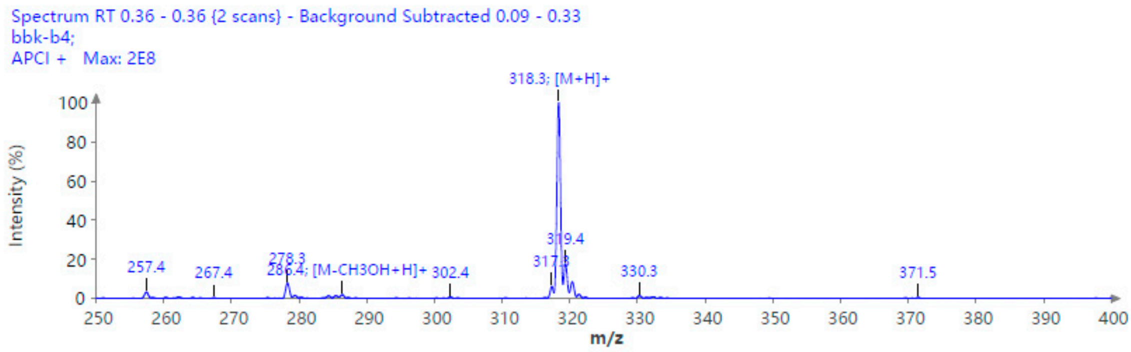

Figure S48: APCI-MS spectra of the compound **2j**

Data File: C:\LabSolutions\Data\Analiz\derya\BBK-B4_62.lcd

Elmt	Val.	Min	Max	Elmt	Val.	Min	Max	Elmt	Val.	Min	Max	Elmt	Val.	Min	Max	Use Adduct
H	1	0	40	O	2	0	4	S	2	1	2	Ru	2	0	0	H
C	4	0	40	F	1	0	0	Cl	1	0	0	Pd	2	0	0	
N	3	2	5	P	3	0	0	Br	1	0	0	I	3	0	0	

Error Margin (ppm): 10
HC Ratio: unlimited
Max Isotopes: 3
MSn Iso RI (%): 10.00

DBE Range: 0.0 - 20.0
Apply N Rule: yes
Isotope RI (%): 1.00
MSn Logic Mode: AND

Electron Ions: both
Use MSn Info: yes
Isotope Res: 9000
Max Results: 150

Event#: 1 MS(E+) Ret. Time : 7.213 Scan# : 1083

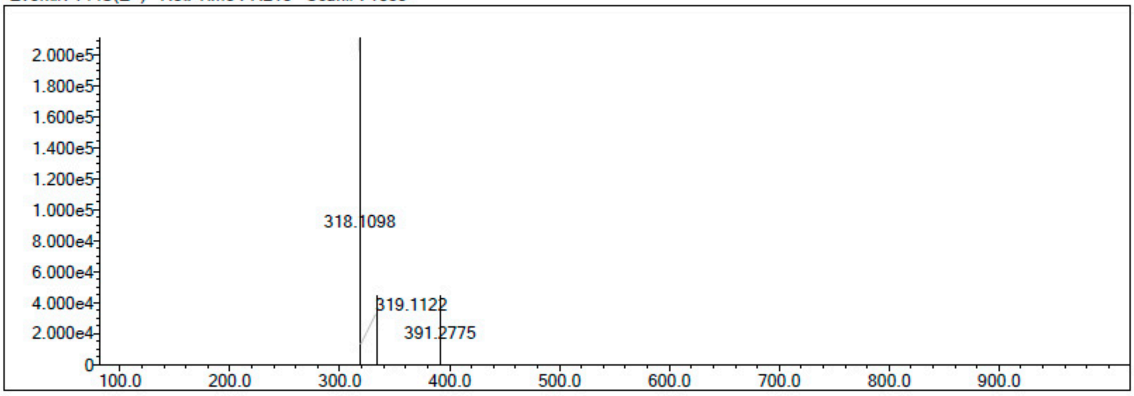

Measured region for 318.1098 m/z

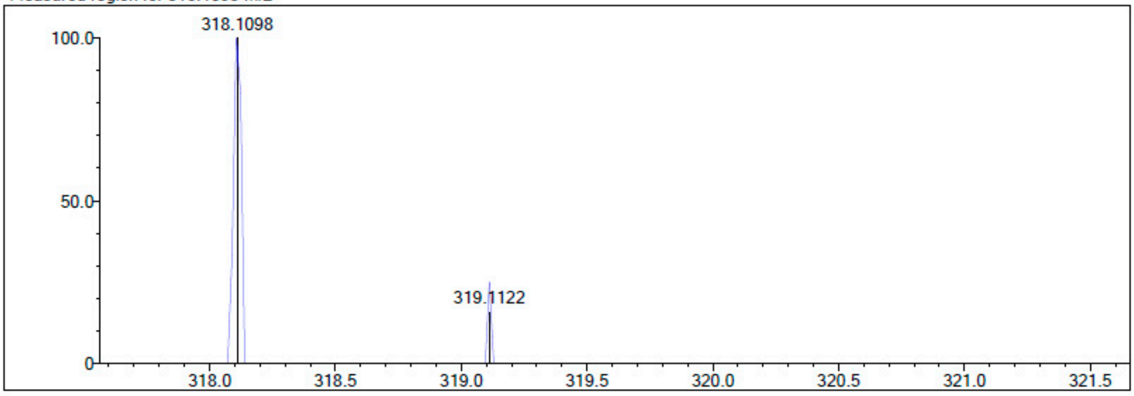

C16 H19 N3 S2 [M+H]+ : Predicted region for 318.1093 m/z

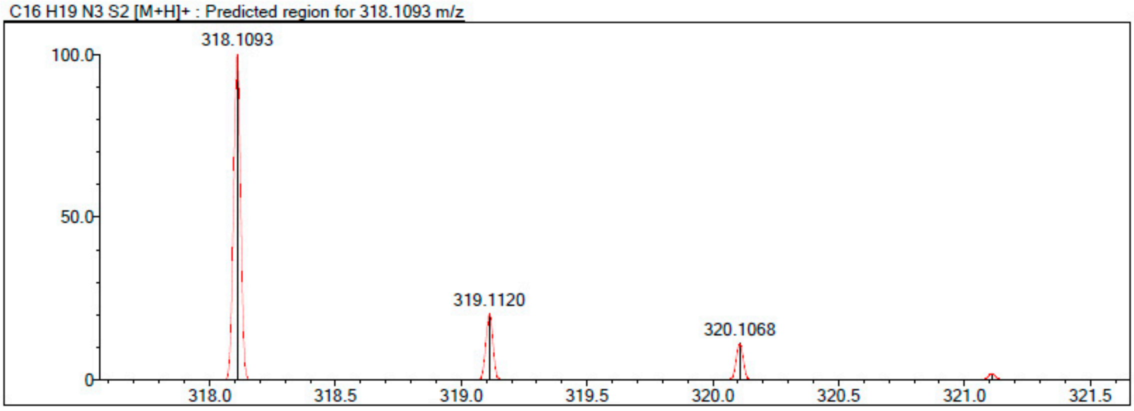

Rank	Score	Formula (M)	Ion	Meas. m/z	Pred. m/z	Df. (mDa)	Df. (ppm)	Iso	DBE
1	0.00	C16 H19 N3 S2	[M+H]+	318.1098	318.1093	0.5	1.57	0.00	9.0

Figure S49: HRMS spectra of the compound **2j**

==== Shimadzu LCMSsolution Analysis Report ====

Acquired by	: Admin
Sample Name	: BBK-B4
Sample ID	:
Vail #	: 25
Injection Volume	: 0.3 uL
Data File Name	: BBK-B4_59.lcd
Method File Name	: isocratic_serkan.lcm
Batch File Name	: batch.lcb
Report File Name	: DefaultLCMS.lcr
Data Acquired	: 13.08.2021 11:11:21
Data Processed	: 13.08.2021 12:24:04

<Chromatogram>

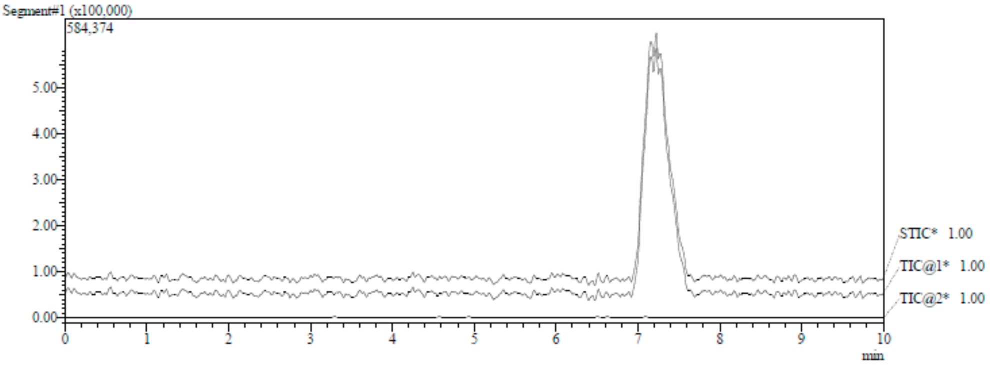

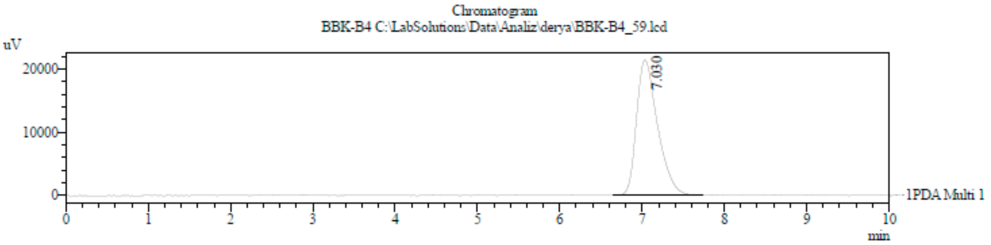

1 PDA Multi 1 / 341nm 4nm

PeakTable

PDA Ch1 341nm 4nm

Peak#	Ret. Time	Area	Height	Area %	Height %
1	7.030	382973	21430	100.000	100.000
Total		382973	21430	100.000	100.000

Figure S50: LCMS spectra of the compound **2j**

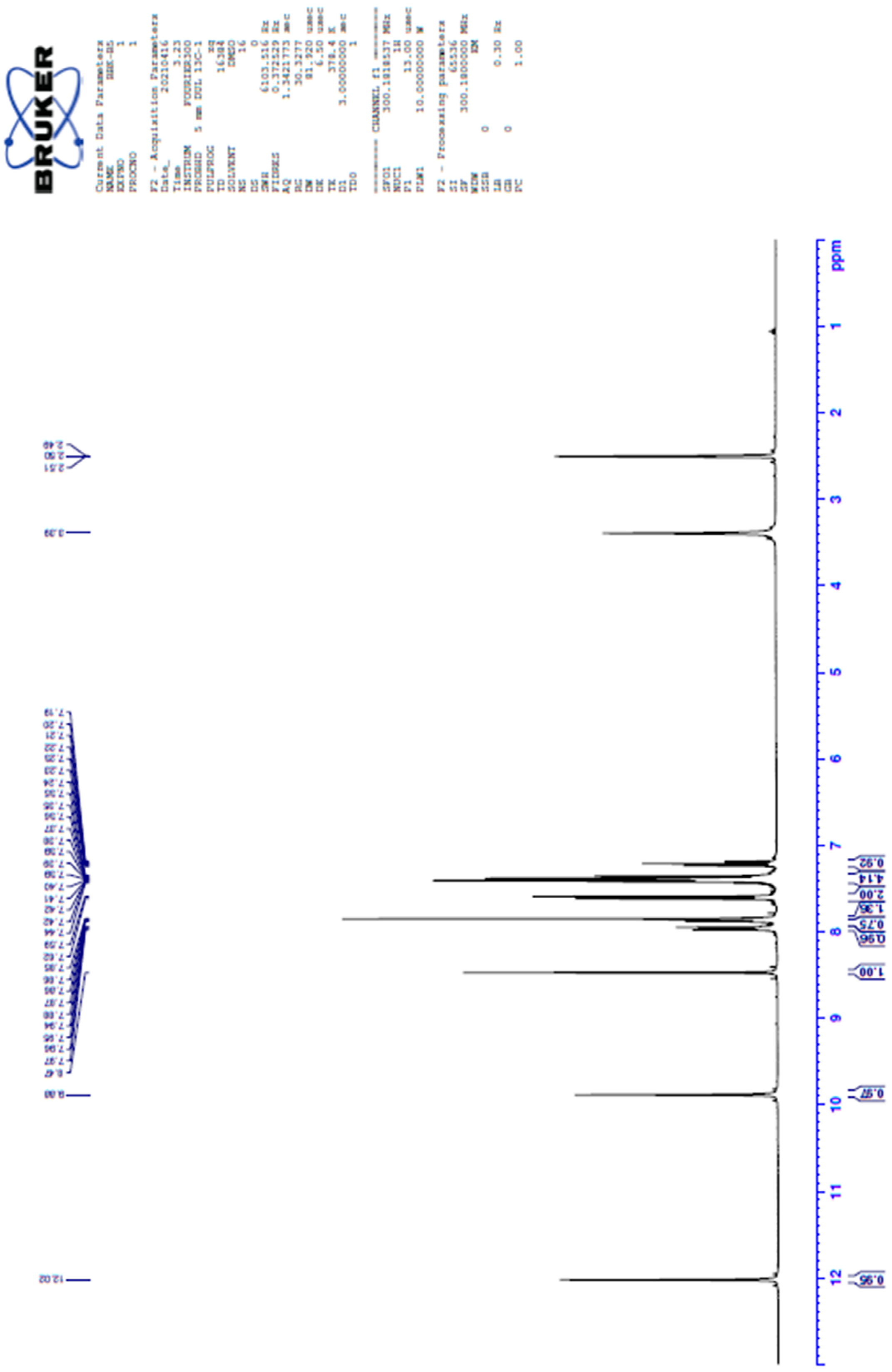

Figure S51: ^{1}H-NMR spectra of the compound **2k**

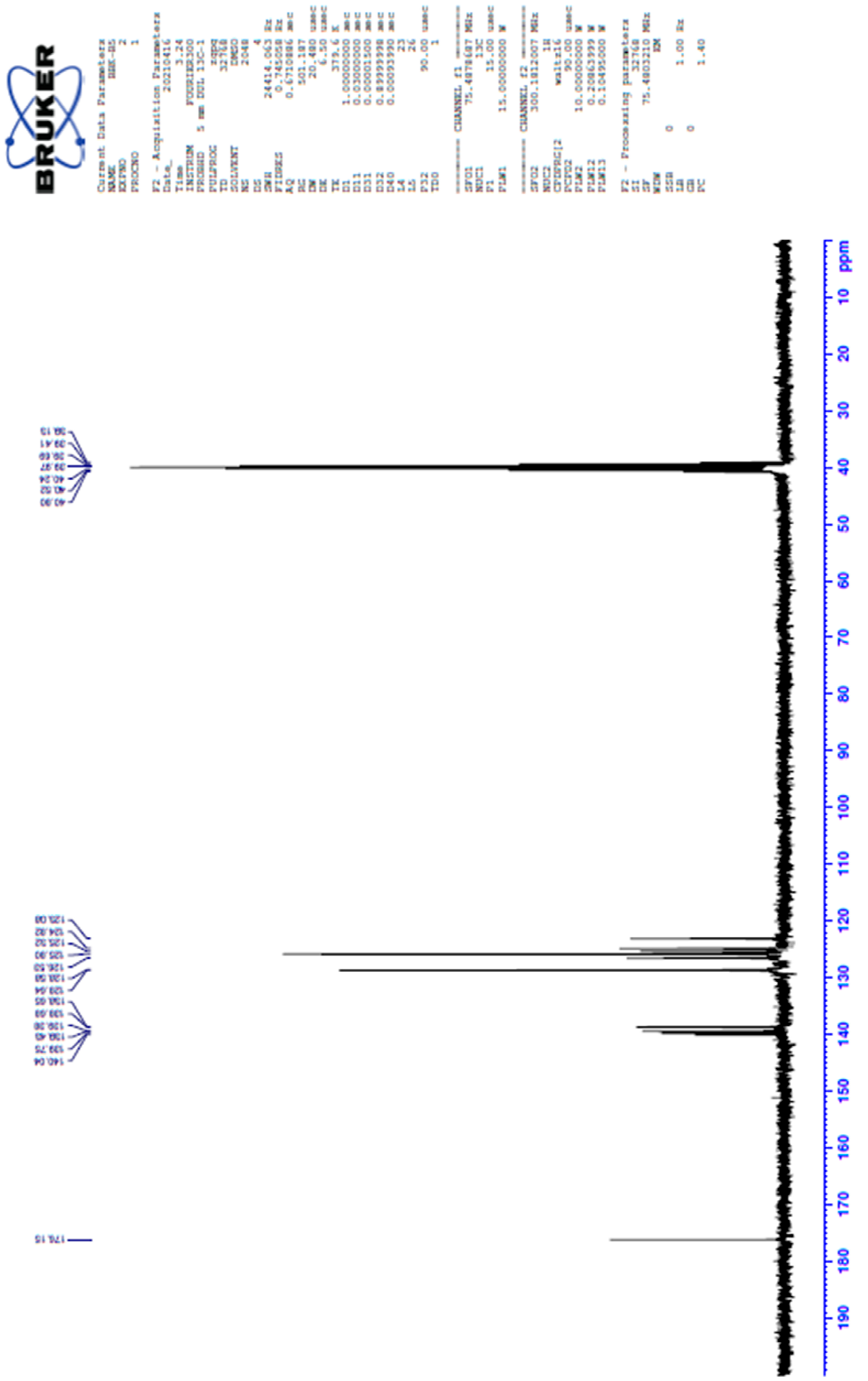

Figure S52: ^{13}C-NMR spectra of the compound **2k**

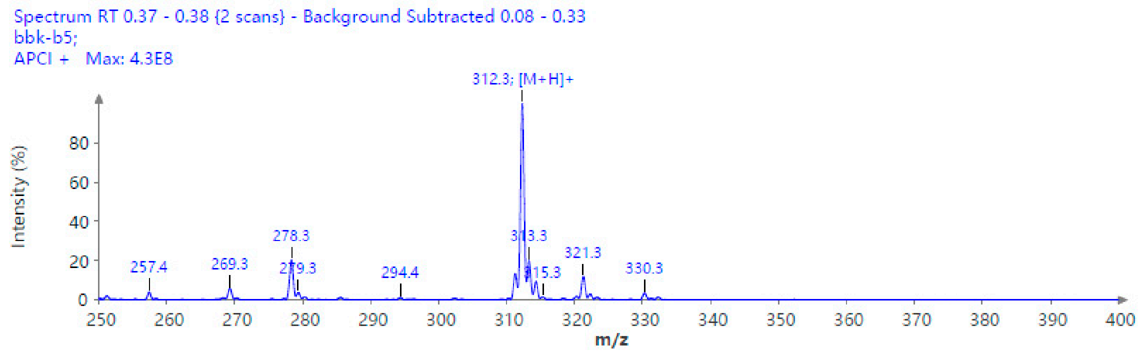

Figure S53: APCI-MS spectra of the compound **2k**

Formula Predictor Report - BBK-B5_5.lcd

Data File: C:\LabSolutions\Data\Analiz\derya\BBK-B5_5.lcd

Elmt	Val.	Min	Max	Elmt	Val.	Min	Max	Elmt	Val.	Min	Max	Elmt	Val.	Min	Max	Use Adduct
H	1	0	40	O	2	0	4	S	2	1	2	Ru	2	0	0	H
C	4	0	40	F	1	0	0	Cl	1	0	1	Pd	2	0	0	
N	3	2	5	P	3	0	0	Br	1	0	0	I	3	0	0	

Error Margin (ppm): 5
HC Ratio: unlimited
Max Isotopes: 3
MSn Iso RI (%): 10.00

DBE Range: 6.0 - 12.0
Apply N Rule: yes
Isotope RI (%): 1.00
MSn Logic Mode: AND

Electron Ions: both
Use MSn Info: yes
Isotope Res: 9000
Max Results: 150

Event#: 1 MS(E+) Ret. Time : 4.613 Scan# : 693

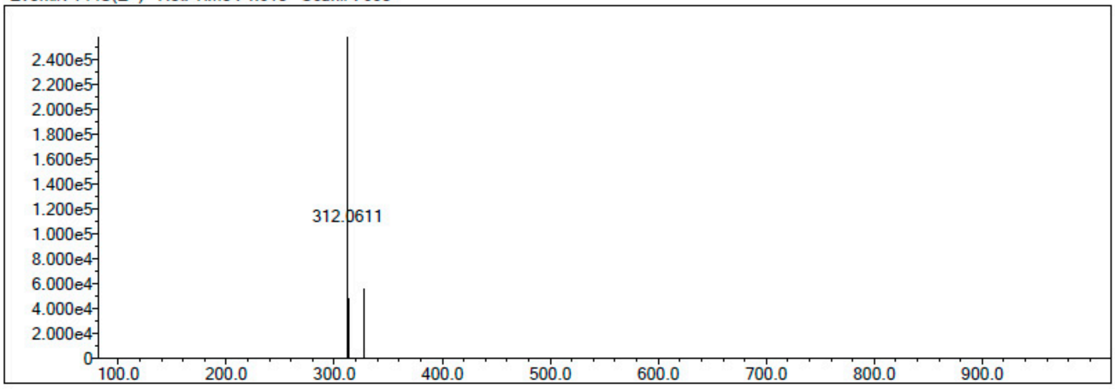

Measured region for 312.0611 m/z

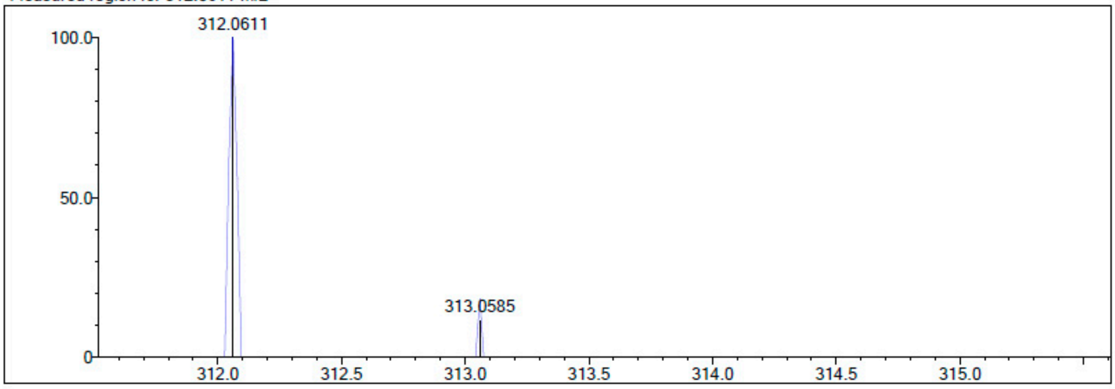

C16 H13 N3 S2 [M+H]+ : Predicted region for 312.0624 m/z

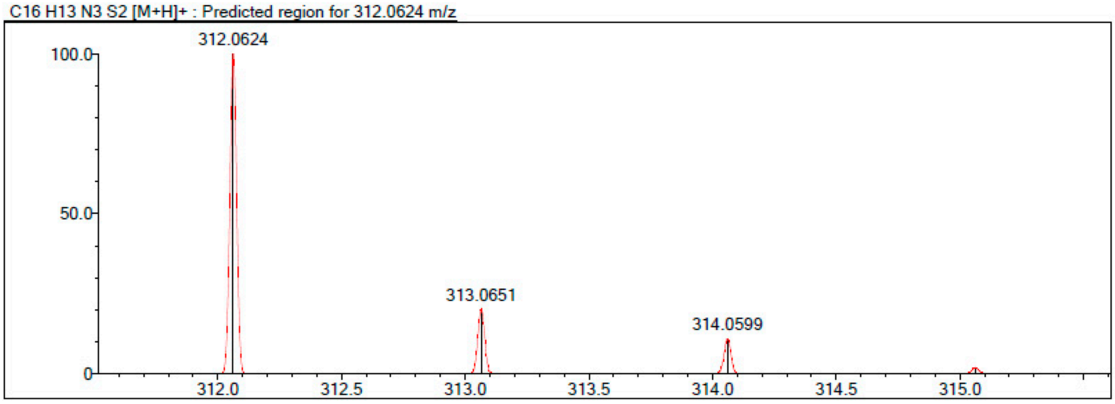

Rank	Score	Formula (M)	Ion	Meas. m/z	Pred. m/z	Df. (mDa)	Df. (ppm)	Iso	DBE
1	0.00	C16 H13 N3 S2	[M+H]+	312.0611	312.0624	-1.3	-4.17	0.00	12.0

Figure S54: HRMS spectra of the compound **2k**

==== Shimadzu LCMSsolution Analysis Report ====

Acquired by	: Admin
Sample Name	: BBK-B5
Sample ID	:
Vail #	: 71
Injection Volume	: 0.3 uL
Data File Name	: BBK-B5_63.lcd
Method File Name	: isocratic_serkan.lcm
Batch File Name	: batch.lcb
Report File Name	: DefaultLCMS.lcr
Data Acquired	: 10.08.2021 16:55:34
Data Processed	: 12.08.2021 14:11:26

<Chromatogram>

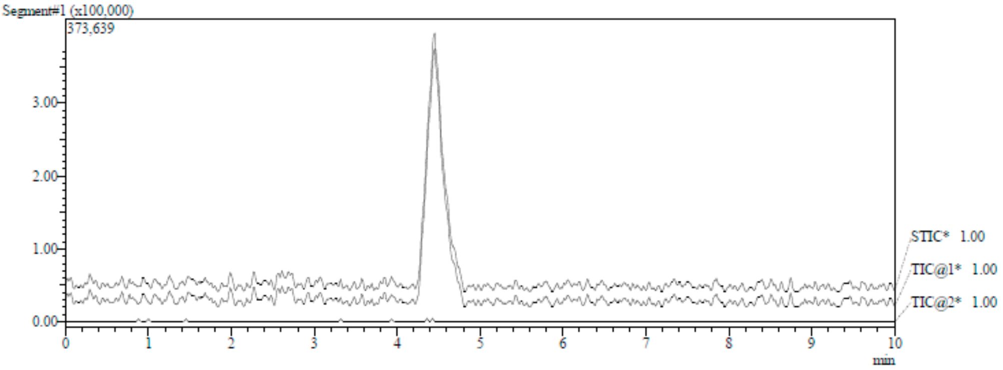

Chromatogram
BBK-B5 C:\LabSolutions\Data\Analiz\derya\BBK-B5_63.lcd

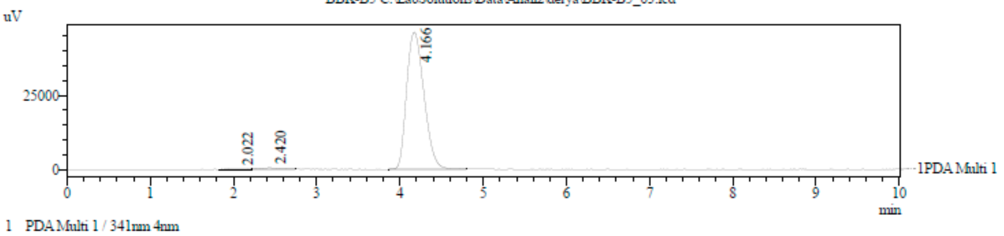

1 PDA Multi 1 / 341nm 4nm

PeakTable

PDA Ch1 341nm 4nm

Peak#	Ret. Time	Area	Height	Area %	Height %
1	2.022	2996	229	0.439	0.485
2	2.420	7546	527	1.105	1.118
3	4.166	672230	46367	98.456	98.397
Total		682771	47122	100.000	100.000

Figure S55: LCMS spectra of the compound **2k**

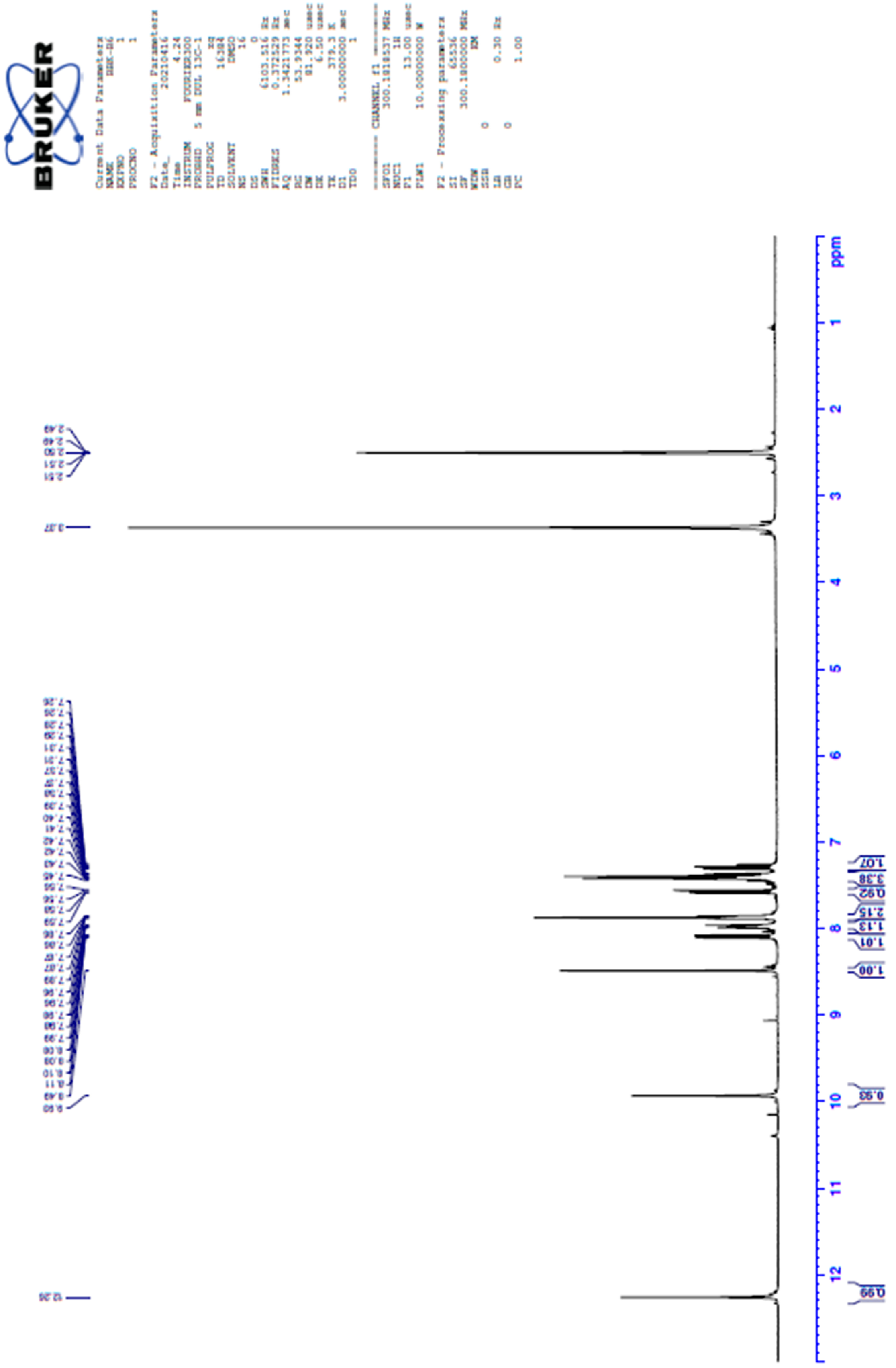

Figure S56: ^{1}H-NMR spectra of the compound **2l**

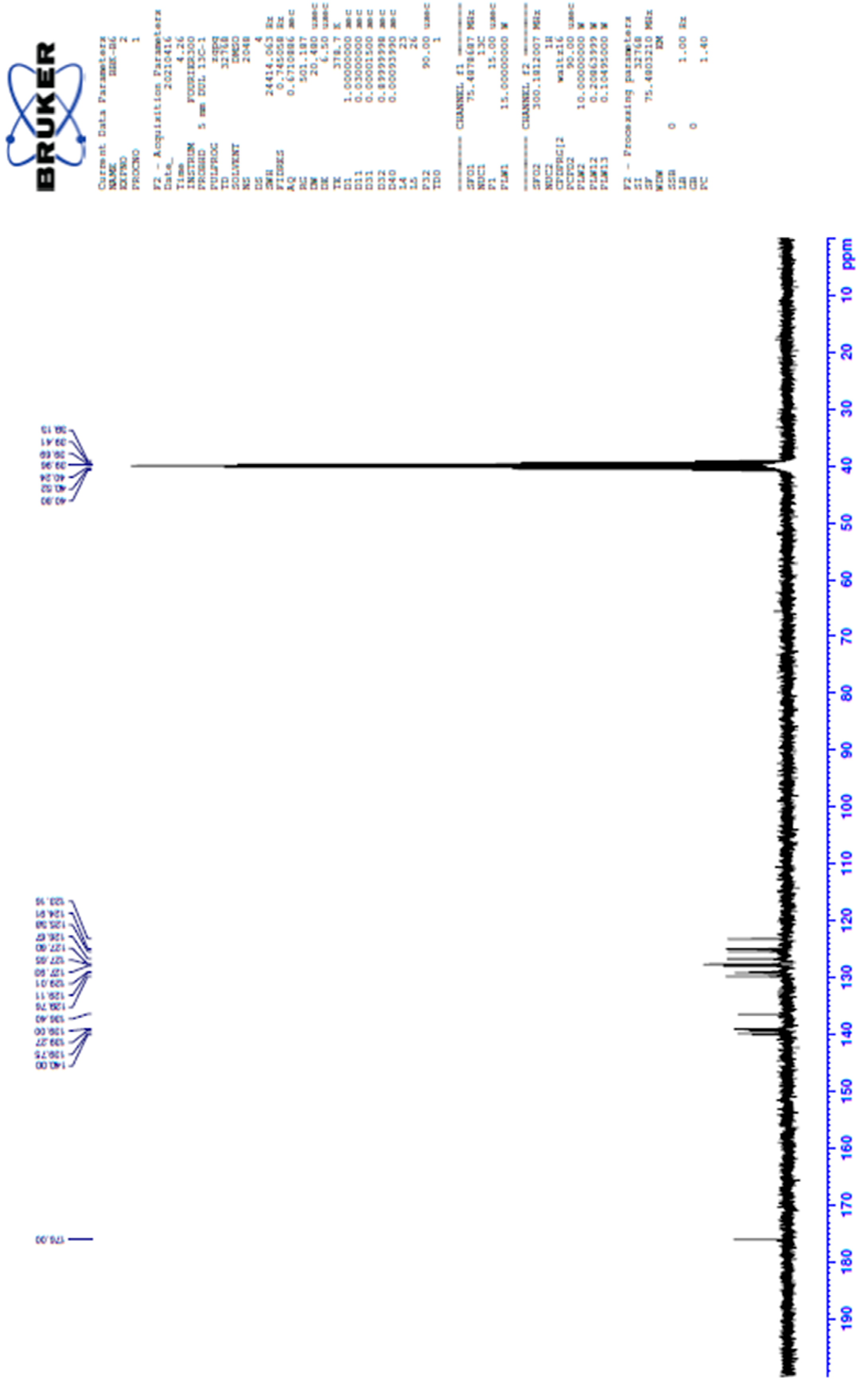

Figure S57: ^{13}C-NMR spectra of the compound **2l**

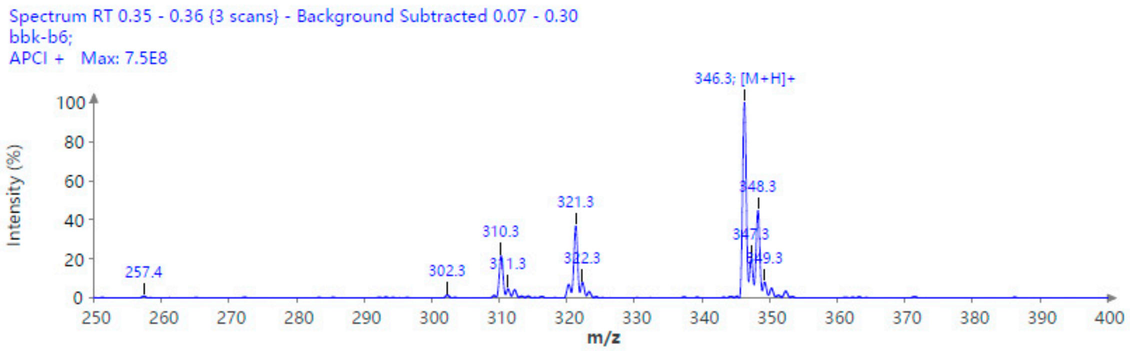

Figure S58: APCI-MS spectra of the compound **2l**

Data File: C:\LabSolutions\Data\Analiz\derya\BBK-B6_64.lcd

Elmt	Val.	Min	Max	Elmt	Val.	Min	Max	Elmt	Val.	Min	Max	Elmt	Val.	Min	Max	Use Adduct
H	1	0	40	O	2	0	4	S	2	1	2	Ru	2	0	0	H
C	4	0	40	F	1	0	0	Cl	1	1	1	Pd	2	0	0	
N	3	2	5	P	3	0	0	Br	1	0	0	I	3	0	0	

Error Margin (ppm): 5
HC Ratio: unlimited
Max Isotopes: 3
MSn Iso RI (%): 10.00

DBE Range: 0.0 - 20.0
Apply N Rule: yes
Isotope RI (%): 1.00
MSn Logic Mode: AND

Electron Ions: both
Use MSn Info: yes
Isotope Res: 9000
Max Results: 150

Event#: 1 MS(E+) Ret. Time : 6.467 Scan# : 971

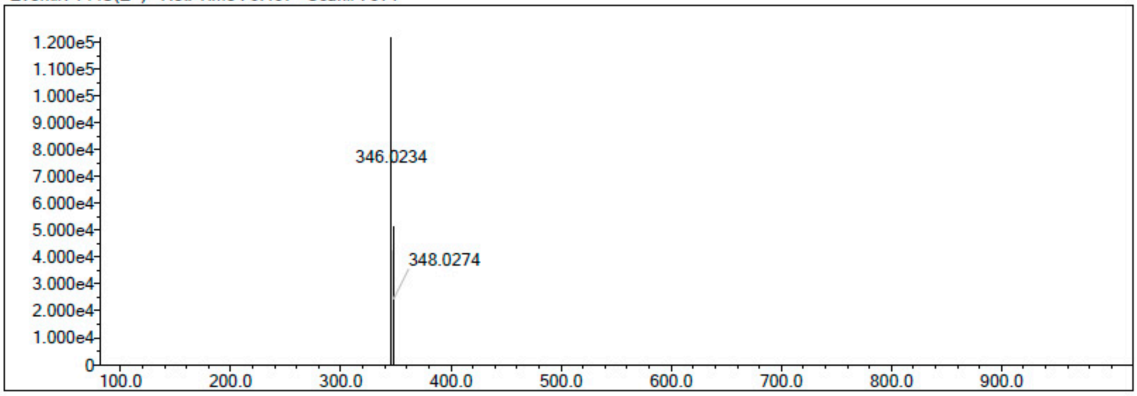

Measured region for 346.0234 m/z

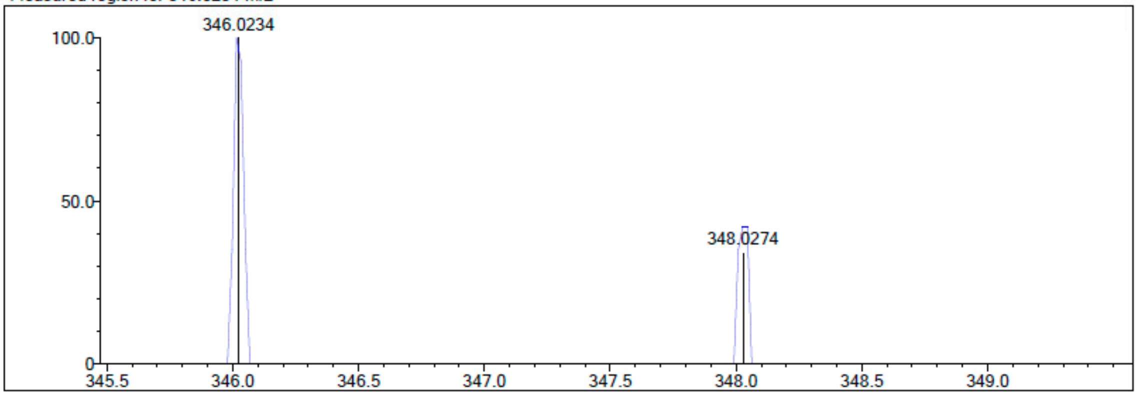

C16 H12 N3 S2 Cl [M+H]+ : Predicted region for 346.0234 m/z

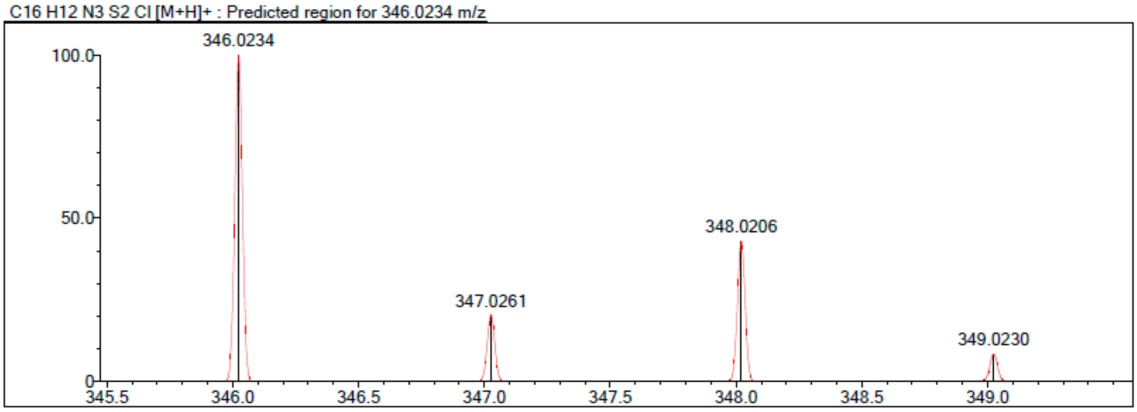

Rank	Score	Formula (M)	Ion	Meas. m/z	Pred. m/z	Df. (mDa)	Df. (ppm)	Iso	DBE
1	59.22	C16 H12 N3 S2 Cl	[M+H]+	346.0234	346.0234	0.0	0.00	59.22	12.0

Figure S59: HRMS spectra of the compound **2l**

==== Shimadzu LCMSsolution Analysis Report ====

Acquired by	: Admin
Sample Name	: BBK-B6
Sample ID	:
Vail #	: 26
Injection Volume	: 0.3 uL
Data File Name	: BBK-B6_60.lcd
Method File Name	: isocratic_serkan.lcm
Batch File Name	: batch.lcb
Report File Name	: DefaultLCMS.lcr
Data Acquired	: 13.08.2021 11:21:55
Data Processed	: 13.08.2021 12:27:01

<Chromatogram>

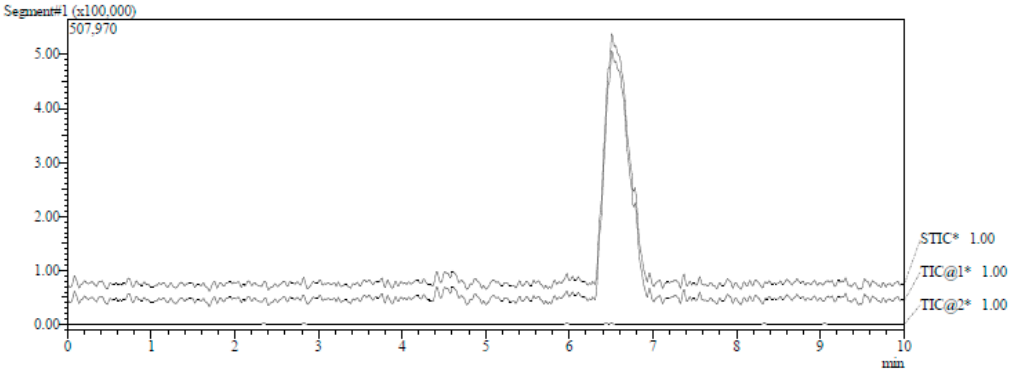

Chromatogram
BBK-B6 C:\LabSolutions\Data\Analiz\derya\BBK-B6_60.lcd

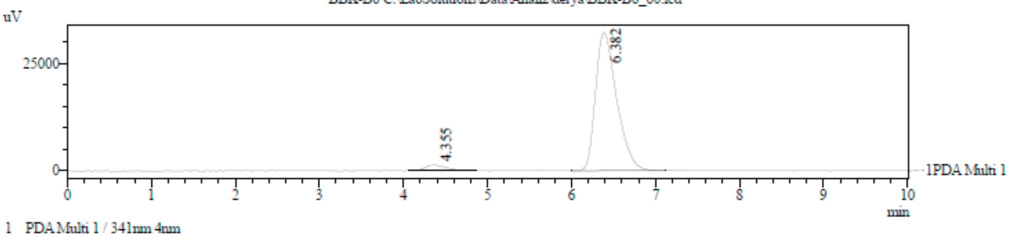

1 PDA Multi 1 / 341nm 4nm

PeakTable

PDA Ch1 341nm 4nm

Peak#	Ret. Time	Area	Height	Area %	Height %
1	4.355	24337	1330	4.135	3.971
2	6.382	564259	32152	95.865	96.029
Total		588596	33482	100.000	100.000

Figure S60: LCMS spectra of the compound **2l**
